# Supplementary material for: Synovial matrix turnover controls immune cell spatial patterning in inflammation resolution
Source: Mol Syst Biol. 2025 Sep 22;21(11):1638–65. doi: 10.1038/s44320-025-00149-7 (PMC12583461; doi:10.1038/s44320-025-00149-7)
Supplement: Supplementary file 1 — Appendix [file 44320_2025_149_MOESM1_ESM.pdf]

Appendix for Synovial matrix turnover controls immune cell spatial patterning in inflammation resolution

Table of Contents 1

Appendix Figure S1. Pipeline for matrisome-based niche mapping. 2

Appendix Figure S2. Dot plot of the core matrisome gene expression levels in each of the total synovial clusters annotated in the Alivernini dataset. 3

Appendix Figure S3. Dot plot of the matrisome-associated gene expression levels in each of the total synovial clusters annotated in the Alivernini dataset. 4

Appendix Figure S4. *De novo* clustering of Alivernini dataset fibroblasts identifies AMP1-like clusters. 5

Appendix Figure S5. Differential expression of matrisome genes in the AMP1 fibroblasts across pseudotime. 7

Appendix Figure S6. Deeper matrix-focused clustering reveals more specific matrix expression niches. 8

Appendix Figure S7. Expression of remission regulated collagen genes across datasets. 9

Appendix Figure S8. Abundance of COL1 and COL6 protein in OA and RA patient samples. 11

Appendix Figure S9. Dot plot of the COL6 and COL6 regulators in fibroblast clusters annotated in the Alivernini (left panel) and Zhang (right panel) dataset. 12

Appendix Figure S10. Network analysis of the COL6 interactome. 13

Appendix Figure S11. Dot plot of COL6 cell surface binding partners in RA vs OA synovial tissue. 14

Appendix Figure S12. Dot plot of COL6 cell surface binding partners active RA vs RA in remission. 15

Appendix Figure S13. Subsynovial niche characterisation across OA and RA sections. 16

Appendix Figure S14. Correlations between COL6A1 niche positivity and cellular abundances. 17

Appendix Figure S15. Collagen VI dark zones are present in IBD and head and neck cancer patients. 19

Appendix Figure S16. COL1A1 and COL6A1 display distinct deposition patterns in RA and OA. 20

Appendix Table S1. 21

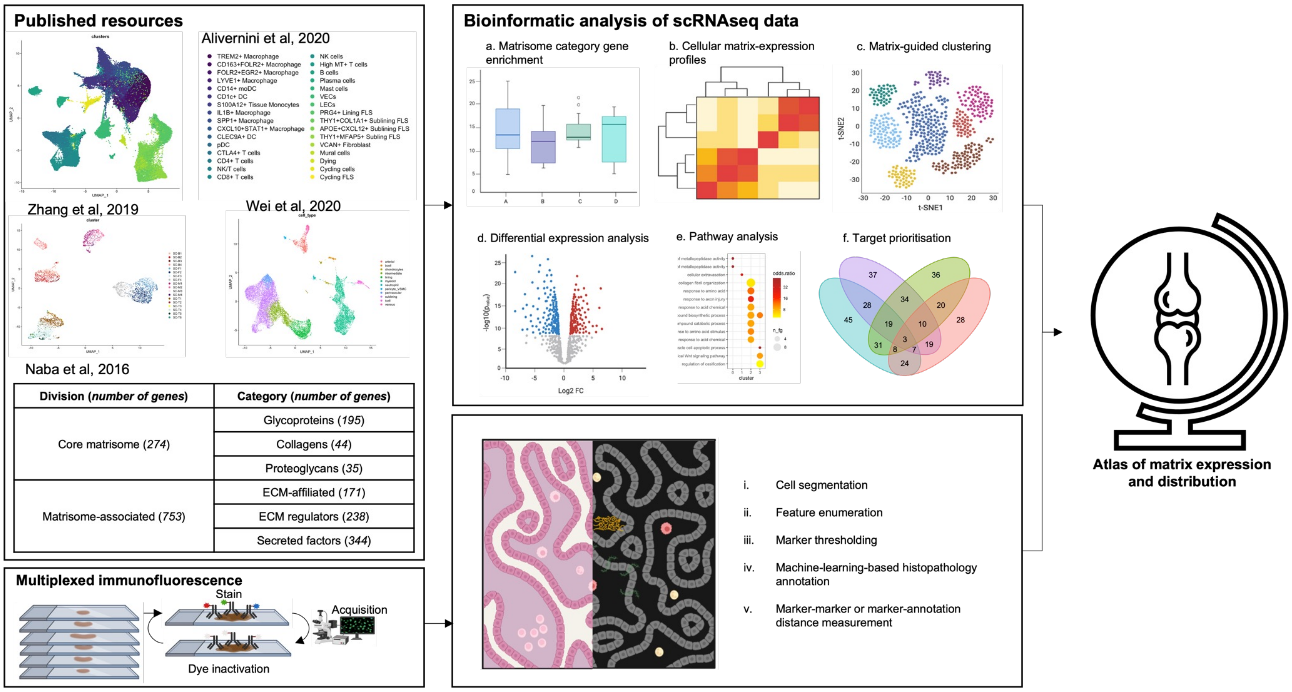

Appendix Figure S1. Pipeline for matrisome-based niche mapping. Briefly, the Alivernini, AMP1, and Wei datasets were imported and their fibroblast subsets reanalysed for subset-specific and condition-specific differential expression of genes forming part of the matrisome. Key genes of interest were then prioritised and their associated proteins were mapped in human RA and OA synovial tissue using multiplexed immunofluorescence. Novel QuPath and SCIMAP pipelines were then used to characterise the spatial relationships between tissue niches, immune cell subsets, and matrix proteins of interest.

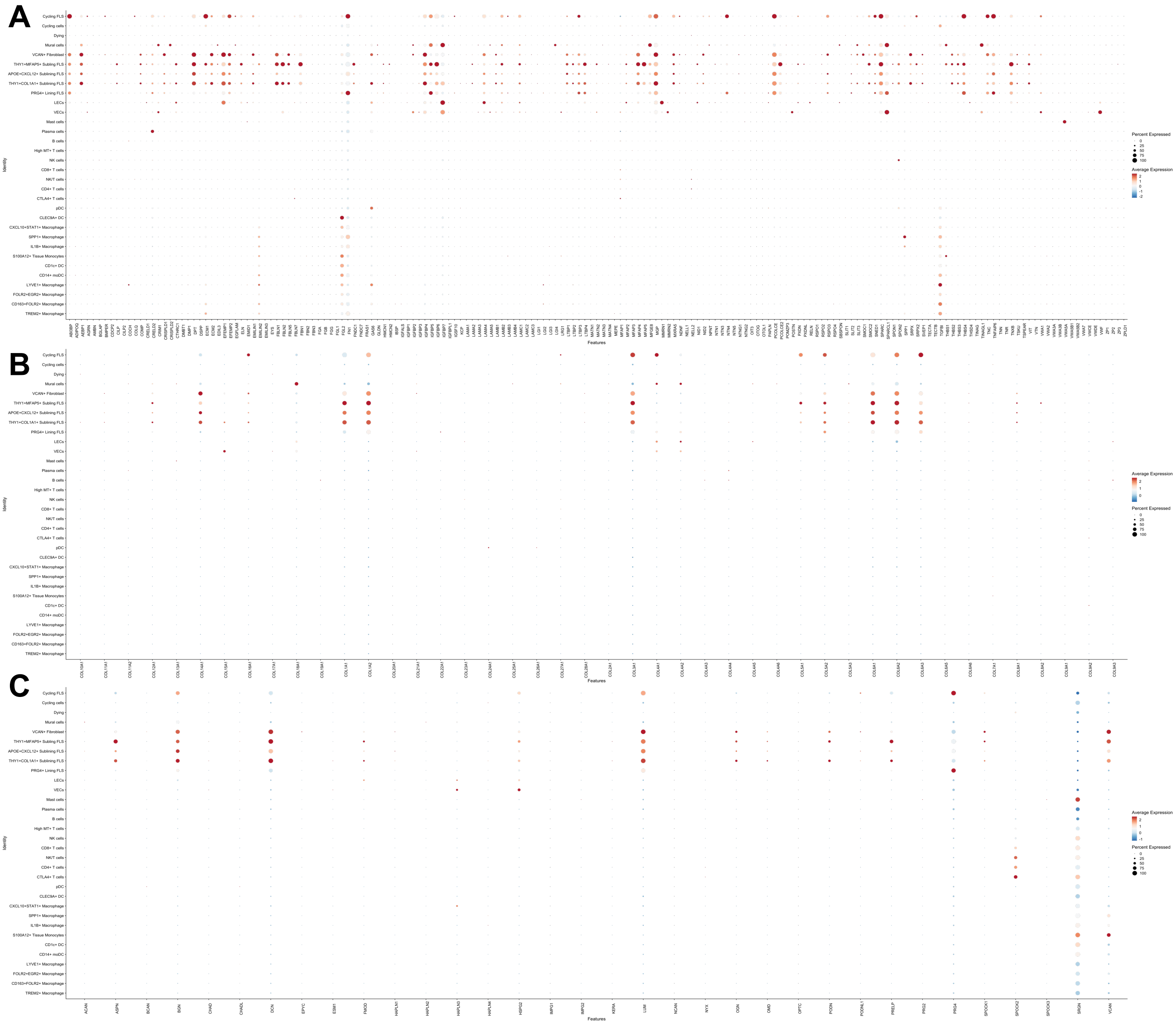

Appendix Figure S2. Dot plot of the core matrisome gene expression levels in each of the total synovial clusters annotated in the Alivernini dataset. Dot size represents the percentage of cells in the cluster expressing the gene of interest, and dot colour represents its level of expression. **A.** Dot plot of matrisome glycoproteins. **B.** Dot plot of matrisome collagens. **C.** Dot plot of matrisome proteoglycans.

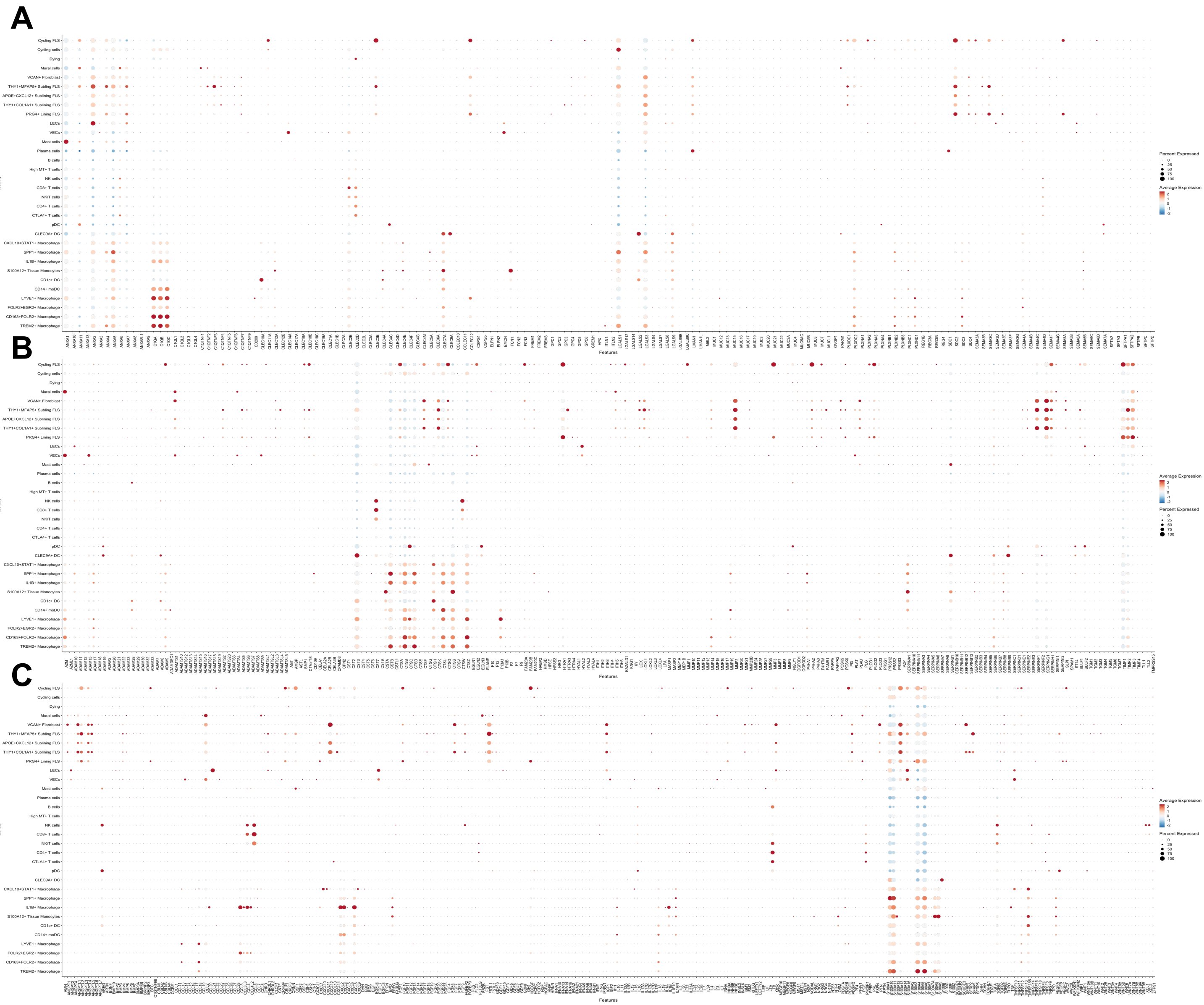

Appendix Figure S3. Dot plot of the matrisome-associated gene expression levels in each of the total synovial clusters annotated in the Alivernini dataset. Dot size represents the percentage of cells in the cluster expressing the gene of interest, and dot colour represents its level of expression. **A.** Dot plot of matrisome ECM-affiliated genes. **B.** Dot plot of matrisome ECM regulators. **C.** Dot plot of matrisome secreted factor genes.

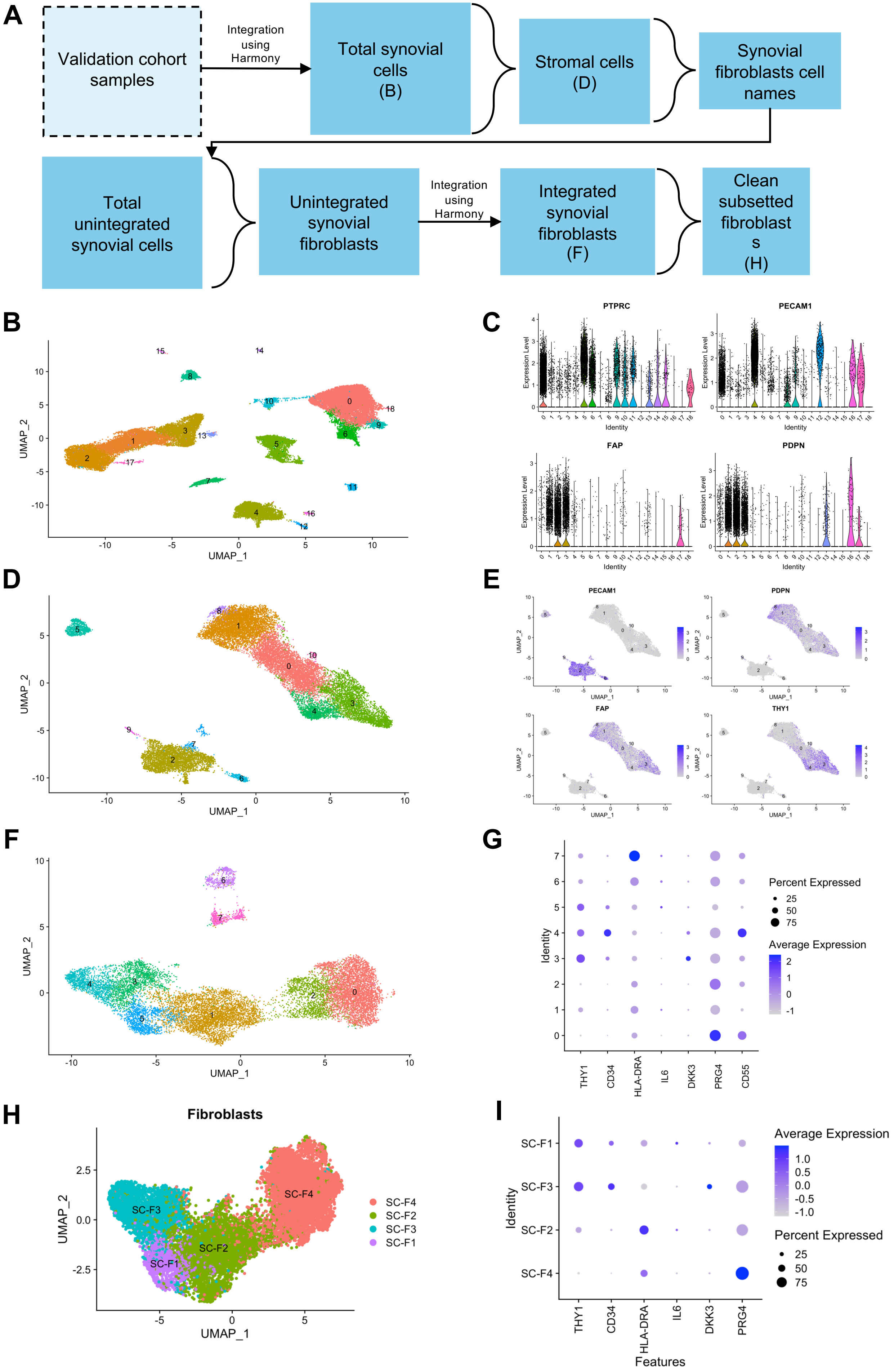
A
Validation cohort samples
Integration using Harmony
Total synovial cells (B)
Stromal cells (D)
Synovial fibroblasts cell names
Total unintegrated synovial cells
Unintegrated synovial fibroblasts
Integration using Harmony
Integrated synovial fibroblasts (F)
Clean subsetted fibroblast s (H)
B
UMAP_1
UMAP_2
C
PTPRC
PECAM1
FAP
PDPN
Expression Level
Identity
D
UMAP_1
UMAP_2
E
PECAM1
PDPN
FAP
THY1
F
UMAP_1
UMAP_2
G
Percent Expressed
25
50
75
Average Expression
Identity
THY1
CD34
HLA-DRA
IL6
DKK3
PRG4
CD55
H
Fibroblasts
SC-F4
SC-F2
SC-F3
SC-F1
UMAP_1
UMAP_2
I
Average Expression
Percent Expressed
Identity
SC-F1
SC-F3
SC-F2
SC-F4
THY1
CD34
HLA-DRA
IL6
DKK3
PRG4
Features

Appendix Figure S4. *De novo* clustering of Alivernini dataset fibroblasts identifies AMP1-like clusters. Total synovial cells from the raw Alivernini dataset were downloaded from the EMBL-EBI under accession number *E-MTAB-8322*. The cells were first integrated using *Harmony*, and computationally subsetted into stromal cells by selecting *PTPRC-* and *PECAM1/FAP/PDPN+* cells. These were then computationally subsetted further into fibroblasts by selecting *PECAM1-* and *PDPN/FAP+* cells. Fibroblast cell identifiers were then collected and used to select only fibroblasts from the raw dataset. These were then integrated from scratch using *Harmony*, and clustered. Clusters were then merged based on the expression of canonical AMP1 fibroblast markers *THY1, CD34, HLA-DRA, IL6, DKK3, PRG4,* and *CD55* to define AMP1-lie clusters. **A.** Schematic workflow of the *de novo* clustering strategy. **B.** UMAP of the total synovial cells from the Alivernini dataset, coloured by cluster. **C.** Violin plots of the expression of markers used to computationally subset stromal cells from total synovial cells in each cell cluster. **D.** UMAP of computationally subsetted stromal cells, coloured by cluster. **E.** Feature plot in UMAP space of the expression of markers used to computationally subset fibroblasts from stromal cells. Marker expression is represented by purple colour intensity. **F.** UMAP of computationally subsetted fibroblasts, coloured by cluster. **G.** Dot plot of the expression of markers used to define the AMP1 fibroblast clusters. **H.** UMAP of computationally subsetted, reintegrated, and clustered fibroblasts, coloured by AMP1-like cluster. **I.** Dot plot of the expression of markers used to define the AMP1 fibroblast clusters.

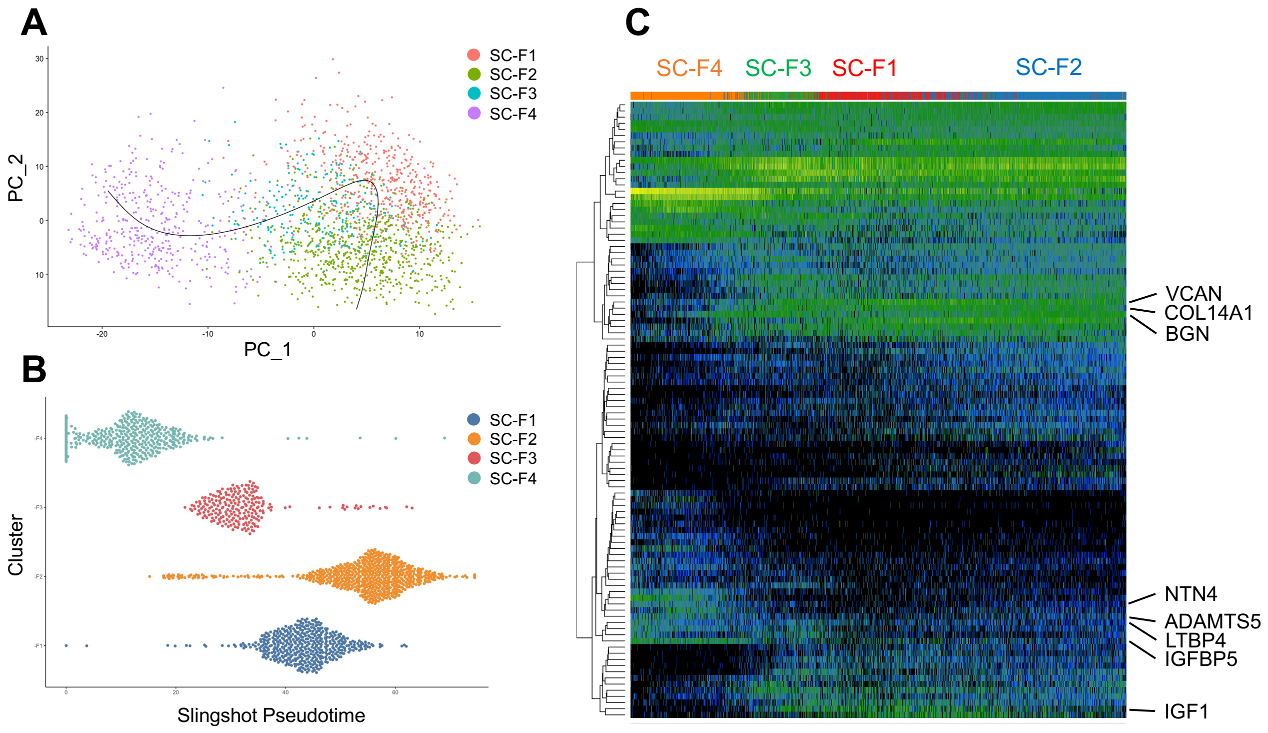

Appendix Figure S5. Differential expression of matrisome genes in the AMP1 fibroblasts across pseudotime. AMP1 fibroblasts were analysed using the *slingshot* package in R. Trajectory fit was performed in PC space, setting the SC-F4 LL fibroblasts as the starting cluster. Trajectory was found to run sequentially through the SC-F4, SC-F3, SC-F1, and SC-F2 fibroblasts. Temporally dynamic matrisome genes were then identfied by fitting a general additive model to test relationships between expression and pseudotime. **A.** Pseudotime trajectory overlayed on the AMP1 fibroblasts in PC space, coloured by cluster. **B.** Violin plot of the AMP1 fibroblasts, grouped and coloured by cluster, and ordered by their position along the pseudotime trajectory (X axis). **C.** Heatmap of the matrisome genes found to be differentially expressed across pseudotime (leftmost of the heatmap is minimum pseudotime, and rightmost is maximum pseudotime).

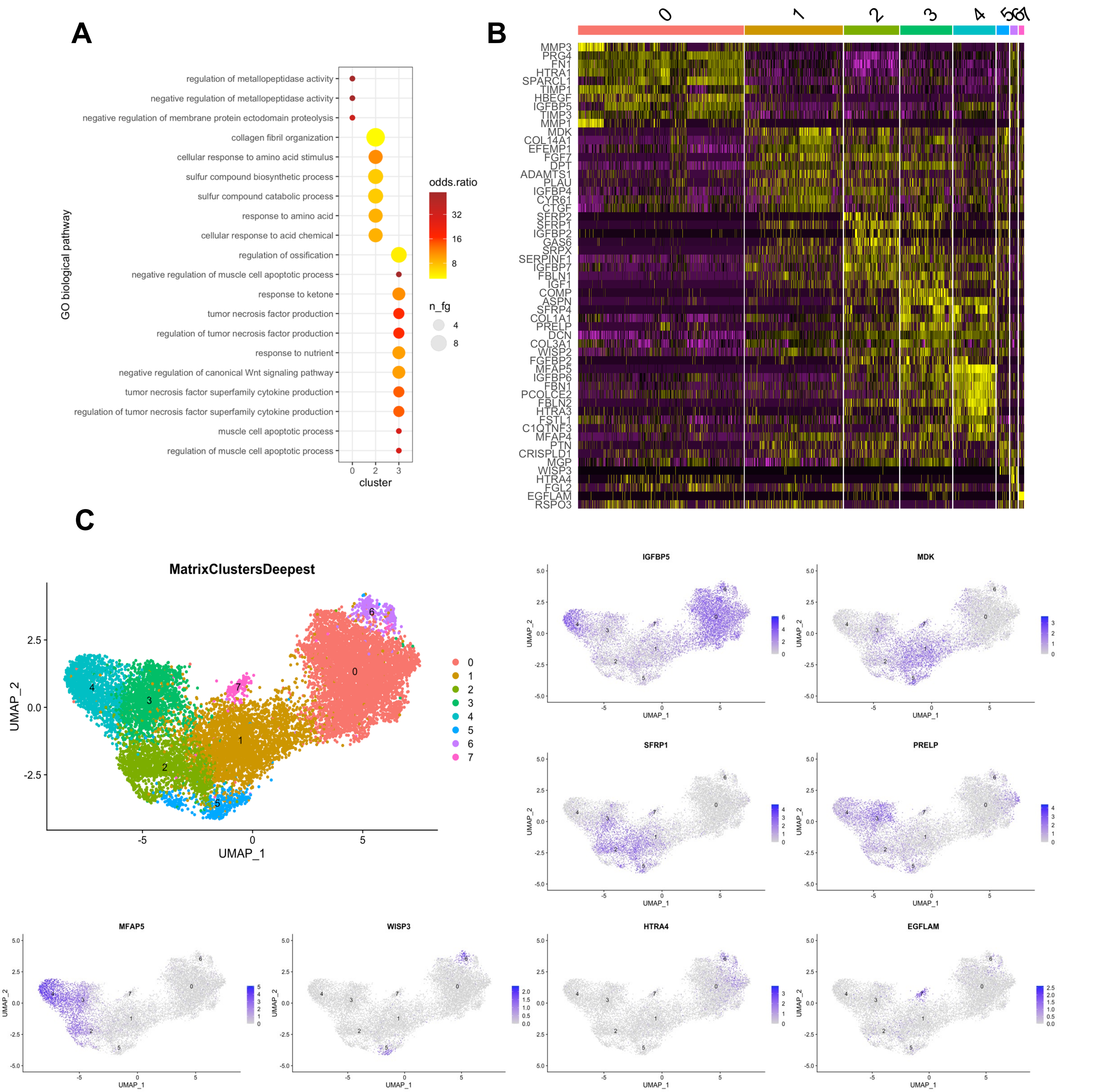

Appendix Figure S6. Deeper matrix-focused clustering reveals more specific matrix expression niches. A. Dot plot of the output pathway from gsfisher-based pathway analysis run on the matrix cluster-defining genes of each cluster presented in Fig. 2D. Dot size represents the number of genes driving the pathway association, while the colour scale represents the odds ratio of the pathway-cluster association. To push the matrix clustering presented in Figure 3.6 further, clusters were also defined using a higher resolution of 0.5. Seurat V4 DEA using a Wilcoxon Rank Sum test was then performed as previously described to define cluster-defining matrix markers. **C.** Heatmap of the top 10 cluster-defining matrix genes for each of the higher resolution (0.5 parameter) matrix-defined clusters. **D.** Feature plots of top cluster defining matrix markers, with gene expression levels represented by purple colour intensity, projected onto UMAP space.

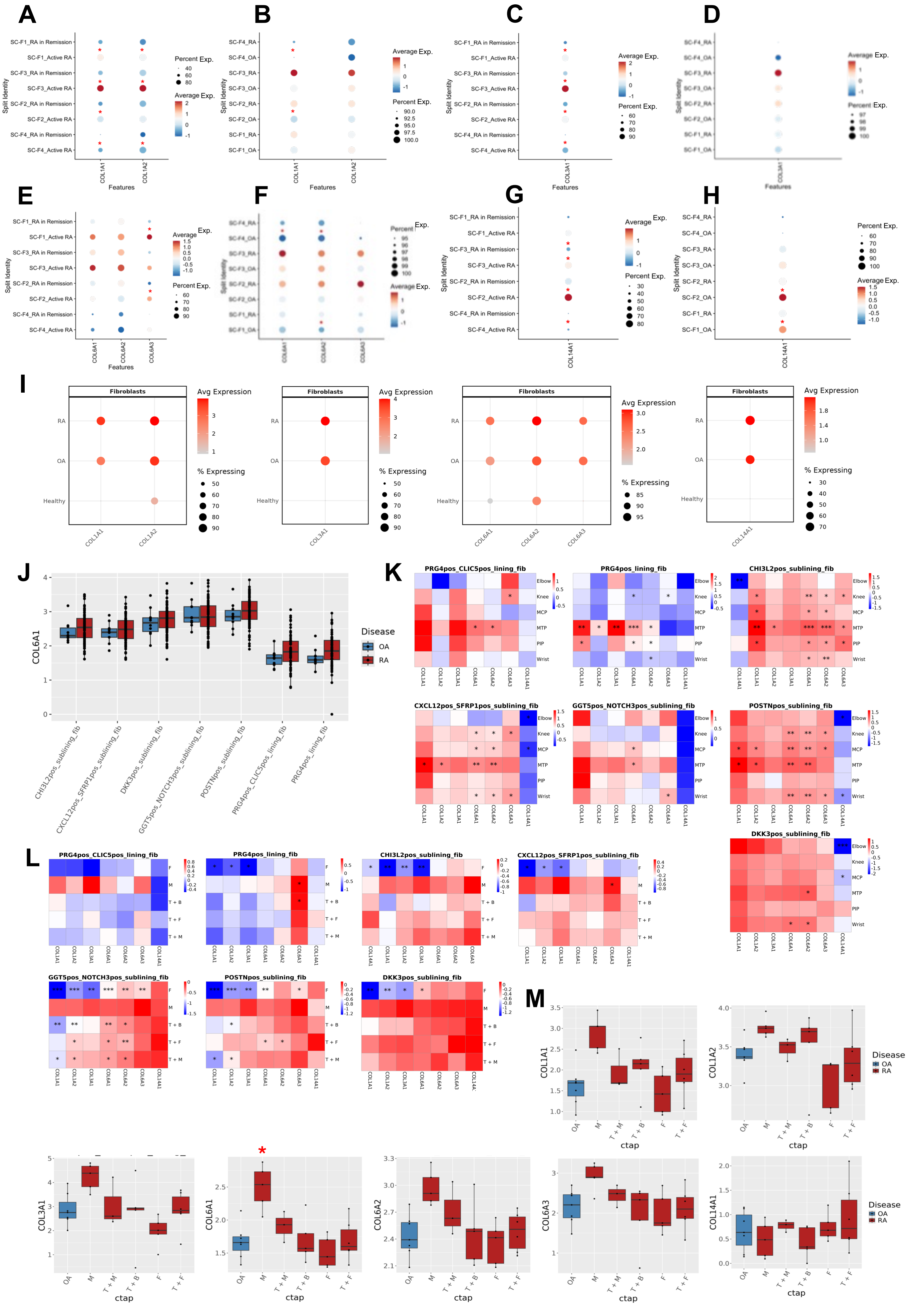

A
SC-F1_RA in Remission
SC-F1_Active RA
SC-F3_RA in Remission
SC-F3_Active RA
SC-F2_RA in Remission
SC-F2_Active RA
SC-F4_RA in Remission
SC-F4_Active RA
Split Identity
COL1A1
COL1A2
Features
Percent Exp.
Average Exp.
B
SC-F4_RA
SC-F4_OA
SC-F3_RA
SC-F3_OA
SC-F2_RA
SC-F2_OA
SC-F1_RA
SC-F1_OA
Average Exp.
Percent Exp.
C
COL3A1
D
E
COL6A1
COL6A2
COL6A3
F
G
COL14A1
H
I
Fibroblasts
RA
OA
Healthy
Avg Expression
% Expressing
J
COL6A1
Disease
OA
RA
CHI3L2pos_sublining_fib
CXCL12pos_SFRP1pos_sublining_fib
DKK3pos_sublining_fib
GGT5pos_NOTCH3pos_sublining_fib
POSTNpos_sublining_fib
PRG4pos_CLIC5pos_lining_fib
PRG4pos_lining_fib
K
PRG4pos_CLIC5pos_lining_fib
PRG4pos_lining_fib
CHI3L2pos_sublining_fib
CXCL12pos_SFRP1pos_sublining_fib
GGT5pos_NOTCH3pos_sublining_fib
POSTNpos_sublining_fib
DKK3pos_sublining_fib
Elbow
Knee
MCP
MTP
PIP
Wrist
L
PRG4pos_CLIC5pos_lining_fib
PRG4pos_lining_fib
CHI3L2pos_sublining_fib
CXCL12pos_SFRP1pos_sublining_fib
GGT5pos_NOTCH3pos_sublining_fib
POSTNpos_sublining_fib
DKK3pos_sublining_fib
F
M
T + B
T + F
T + M
M
COL1A1
COL1A2
COL3A1
COL6A1
COL6A2
COL6A3
COL14A1
ctap
OA
M
T + M
T + B
F
T + F
Disease
OA
RA

Appendix Figure S7. Expression of remission regulated collagen genes across datasets.

A-H. Dot plots showing expression of COL1A1, COL1A2, COL3A1, COL6A1, COL6A2, COL6A3 and COL14A1 in active RA vs remission **(A, C, E, G)** and RA vs OA **(B, D, F, H)**. Dot size represents the percentage of cells in the cluster expressing the gene of interest, and dot colour represents its level of expression. Red stars represent differential expression of the labelled comparison characterised by an adjusted p-value < 0.05 and a log2FC >0.58 or <-0.58. **I.** Dot plots display expression of collagen genes from healthy (Faust dataset), RA and OA (integrated disease dataset) across all annotated fibroblasts. Dots are coloured by log normalised expression, and size reflects the percentage of cells expressing that gene. **J.** Box plots compare expression of collagen genes, across fibroblast clusters, between OA and RA patients. The combined RA and OA (TAURUS, Alivernini and AMP2) data set was pseudo bulked by patient and cluster and the log normalised expression for each patient is displayed (dots). **K.** The combined Taurus, AMP2 and Alivernini single cell data set was filtered to include RA patients only. Data was pseudo-bulked by patient and cluster and a linear regression model was run for each fibroblast cluster to examine the effect of site on expression of collagens, relative to the reference site - ankle. Heatmaps display the effect size relative to knee, with positive values indicating increased expression and negative values indicating decreased expression. * p < 0.05, ** p < 0.01, ***p<0.001. **L.** The combined Taurus, AMP2 and Alivernini single cell data set was filtered to include RA patients only. Data was pseudo-bulked by patient and cluster and a linear regression model was run for each fibroblast cluster to examine the effect of CTAPs on expression of collagens, relative to the reference CTAP:EFM. Heatmaps display the effect size relative to CTAP:EFM, with positive values indicating increased expression and negative values indicating decreased expression. * p < 0.05, ** p < 0.01, ***p<0.001. CTAPS: M = myeloid, E+F +M = endothelial fibroblast myeloid, F=fibroblast, T + B = T-cell B-cell, T + M = T cell Myeloid. **M.** Box plots compare expression of collagen genes, within the PRG4posCLIC5pos_lining population, between OA and different RA CTAPs. The combined RA and OA (TAURUS, Alivernini and AMP2) data set was filtered to samples obtained from the knee only and of which we have CTAP information is available. The data was pseudo bulked by patient and cluster and the log normalised expression for each patient is displayed (dots).

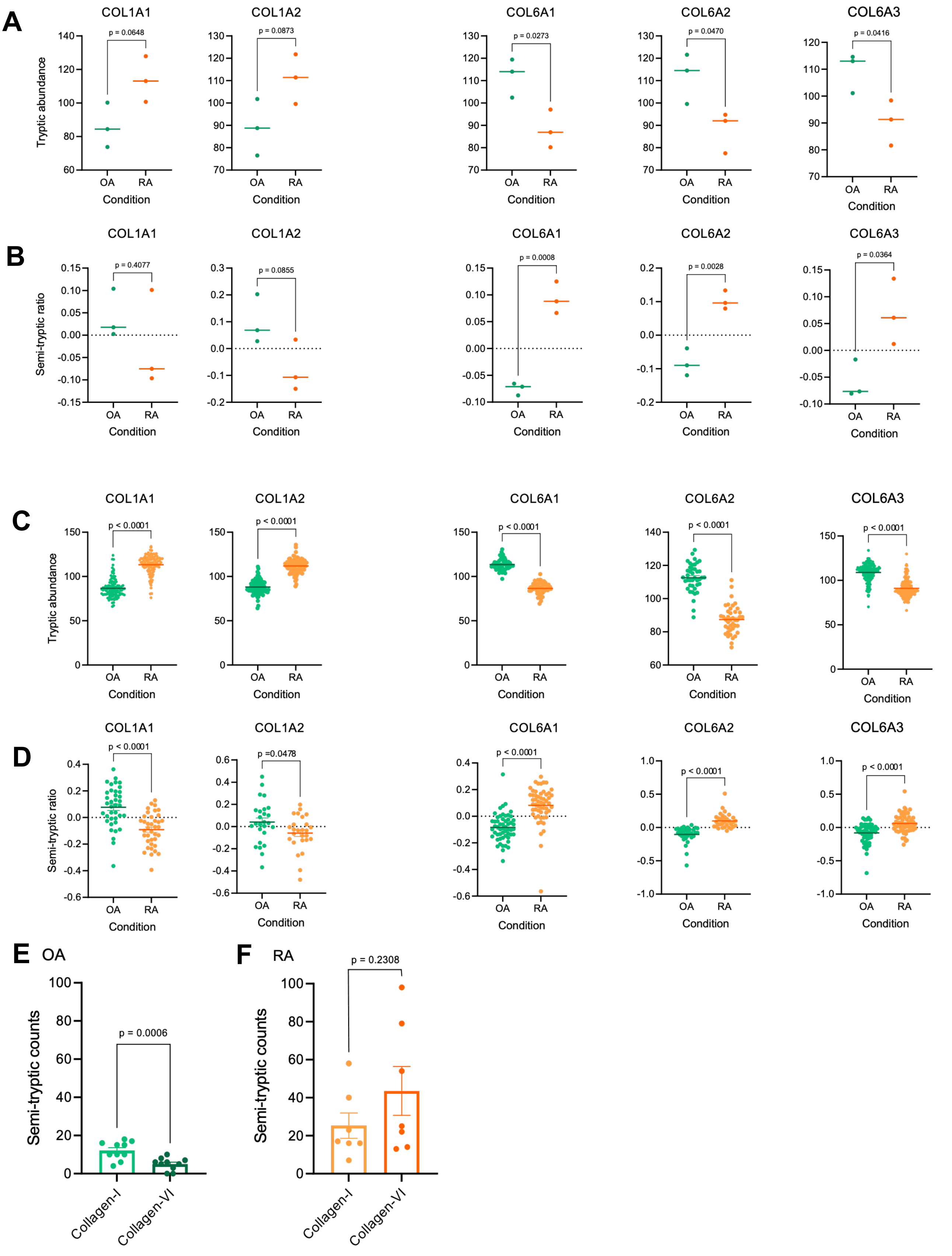

Appendix Figure S8. Abundance of COL1 and COL6 protein in OA and RA patient samples. A. Abundance of individual COL1 and COL6 chains in OA and RA patient samples, n=3. **B.** The corresponding ratios of semi-tryptic peptide abundance compared to tryptic peptide abundance as an indicator of degradation in OA and RA, 10/12 patients respectively, pooled into 3 MS samples. **C.** As in **(A)** but the abundance of each tryptic peptide has been plotted. **D.** As in **(B)** but the ratio for each semi-tryptic peptide has been plotted. **E-F.** Supporting data from **(E)** OA synovial fluid (N=10) and **(F)** RA synovial biopsy (N=8) datasets. Semi-tryptic counts for Collagen-I and –IV in the respective datasets.

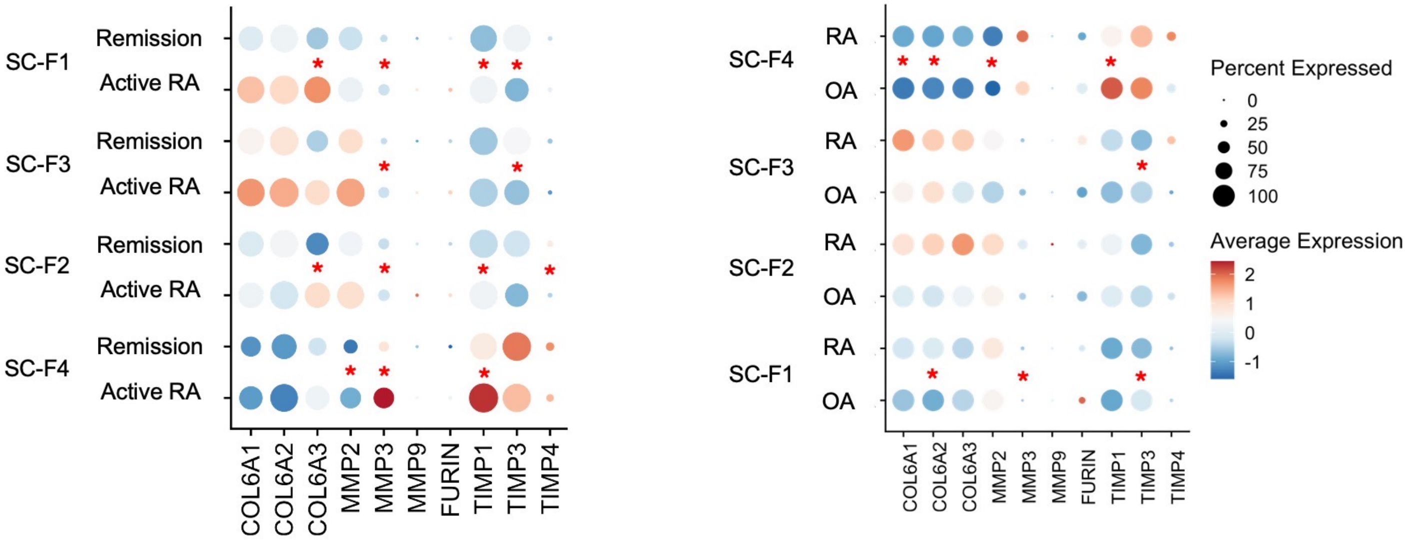

Appendix Figure S9. Dot plot of the COL6 and COL6 regulators in fibroblast clusters annotated in the Alivernini (left panel) and Zhang (right panel) dataset. Dot size represents the percentage of cells in the cluster expressing the gene of interest, and dot colour represents its level of expression.

A

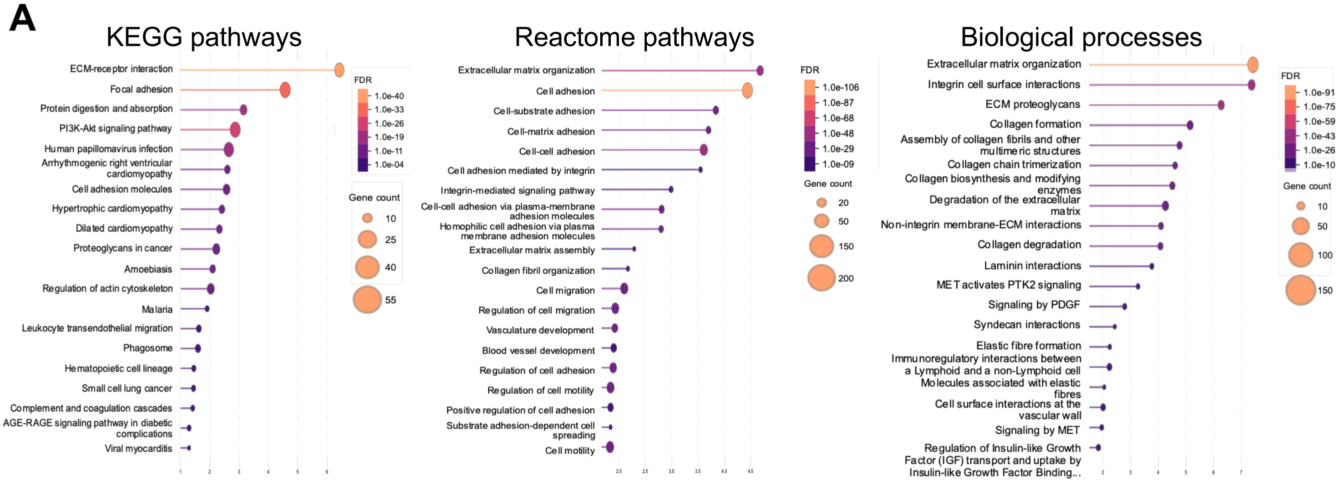

B

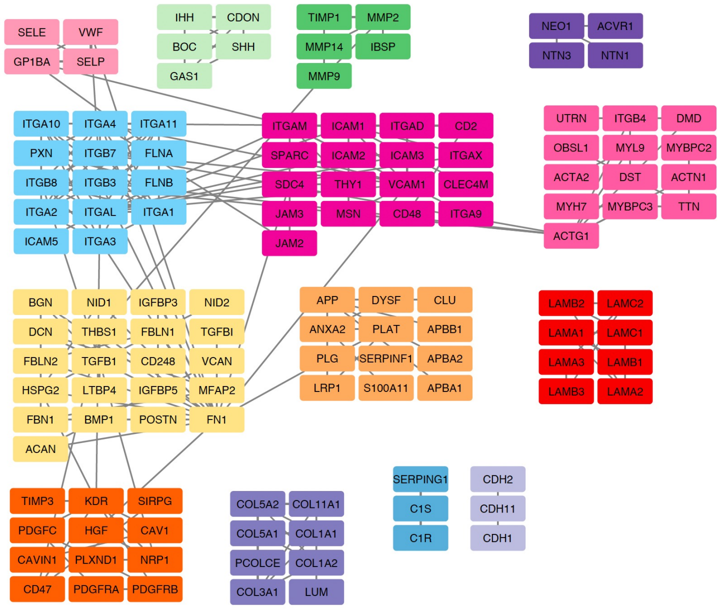

C

ACVR1	ITGA4
APP	ITGA9
BOC	ITGAD
CD2	ITGAL
CD47	ITGAM
CD48	ITGAX
CD248	ITGB3
CDH11	ITGB4
CDH1	ITGB7
CDH2	ITGB8
CDON	JAM2
CLEC4M	JAM3
GAS1	LRP1
GP1BA	MMP14
ICAM1	NEO1
ICAM2	NRP1
ICAM3	PDGFRA
ICAM5	PDGFRB
ITGA1	PLXND1
ITGA10	SDC4
ITGA11	SELE
ITGA2	SELP
ITGA3	THY1
	VCAM1

Appendix Figure S10. Network analysis of the COL6 interactome. A. Pathway analysis of COL6A1, A2 and A3 interacting genes. **B.** Network analysis of the Collagen-VI interactome. Proteins (nodes) are coloured by their cluster, connections (edges) are defined by STRING identified interactions. Clusters were determined from community structure as shown previously. **C.** List of high confidence COL6 binders that locate to the plasma membrane.

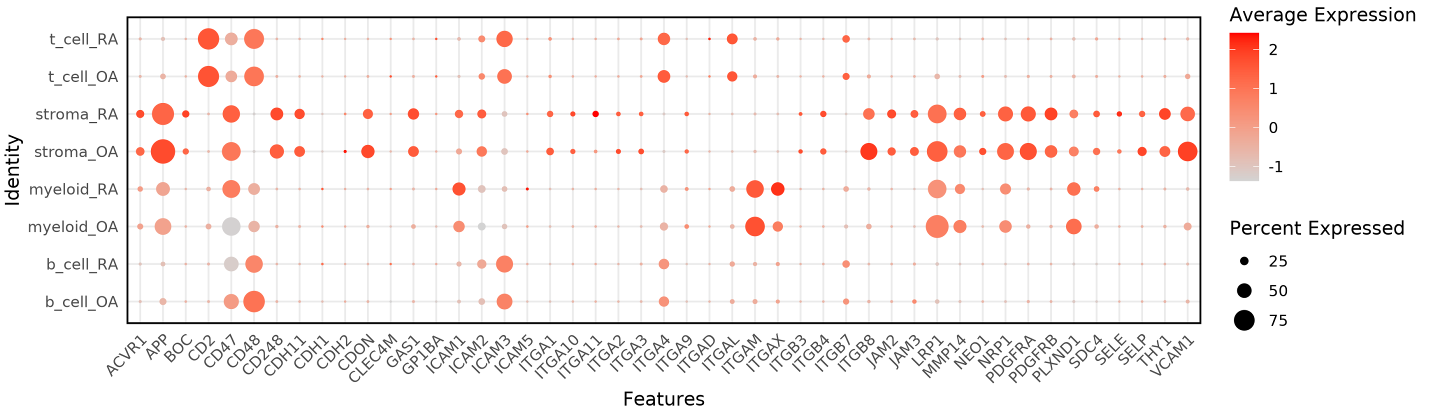

Appendix Figure S11. Dot plot of COL6 cell surface binding partners in RA vs OA synovial tissue. Dot plots display expression of genes from the combined RA and OA (TAURUS, Alivernini and AMP2) data set, across different cell types and disease. Dots are coloured by scaled log normalised expression, and size reflects the percentage of cells expressing that gene.

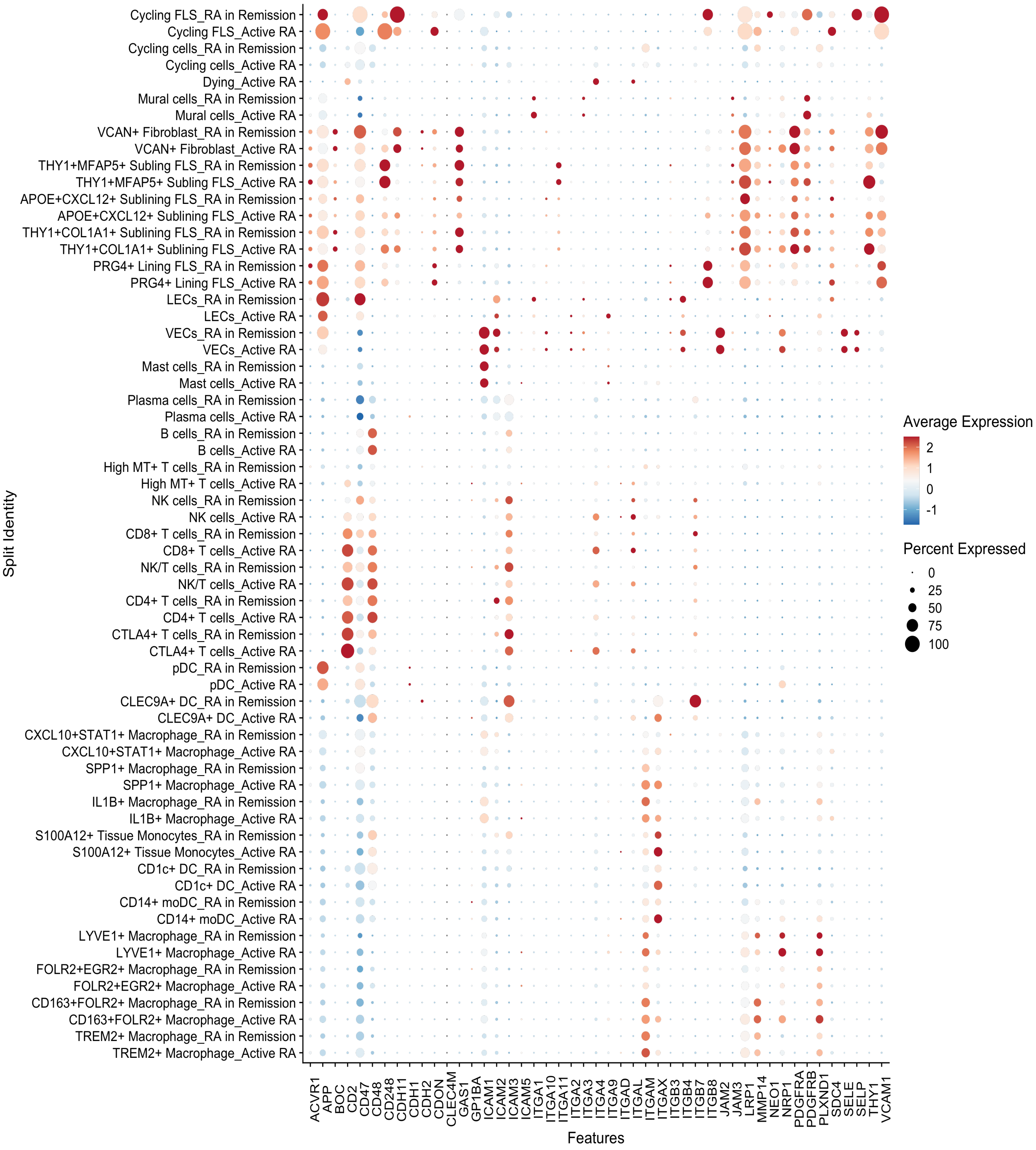

Appendix Figure S12. Dot plot of COL6 cell surface binding partners active RA vs RA in remission. Binding partners from COL6 interactome analysis. Dot size represents the percentage of cells in the cluster expressing the gene of interest, and dot colour represents its level of expression.

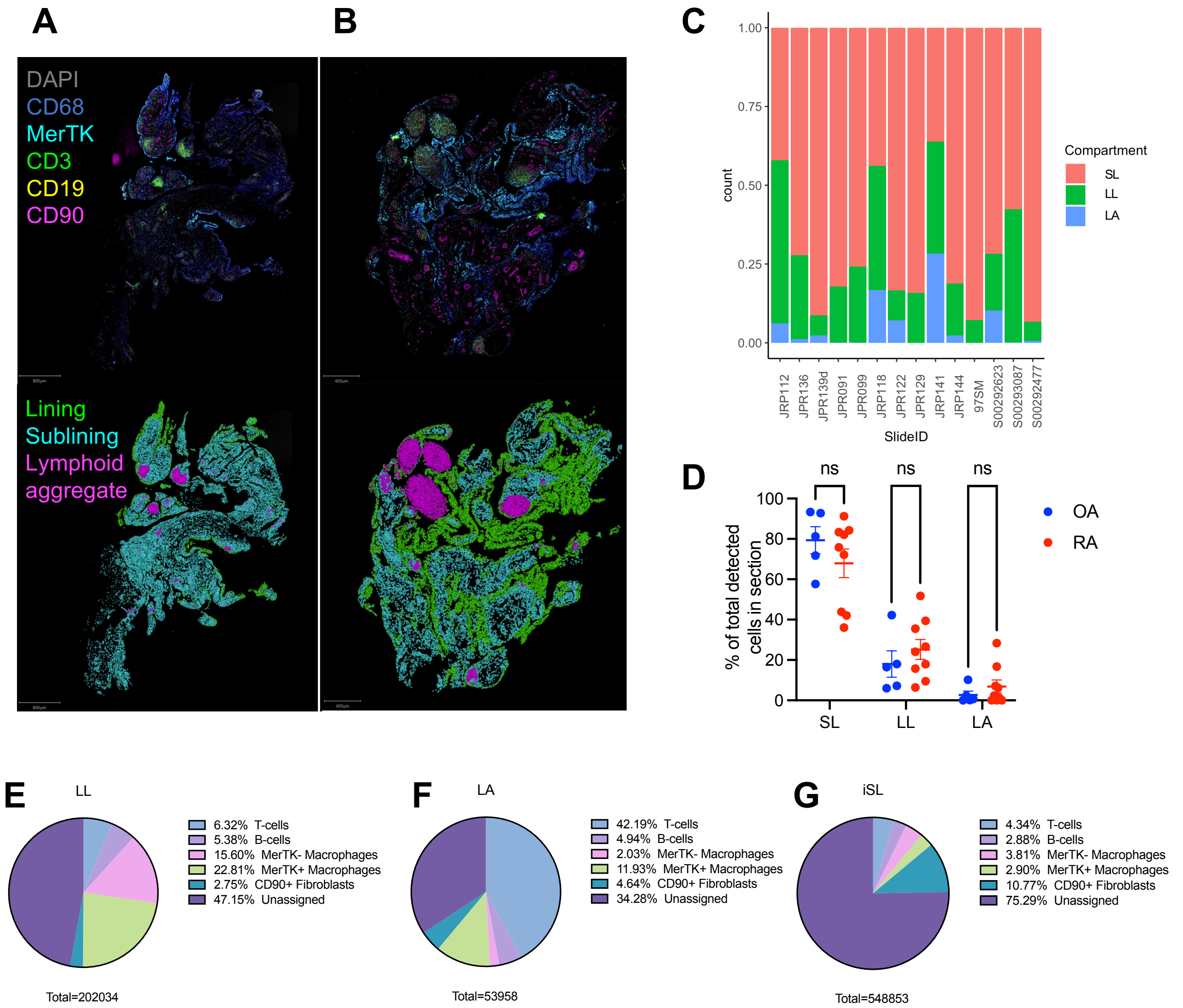

Appendix Figure S13. Subsynovial niche characterisation across OA and RA sections. 804,845 detected cells across the 14 stained and analysed sections were classified by manually training an automated cell classifier in QuPath, using the Random Trees algorithm. Detected cells were then classified into Lining Layer (LL), Lymphoid Aggregates (LA), and Sublining cells (SL), based on the expression of all markers in the panel. **A.** Visualisation in QuPath of section JRP122 immunofluorescent signal for DAPI, MerTK, CD3, CD19, and CD90 (top), and detected cell subsynovial niche classifications (bottom). **B.** Visualisation in QuPath of section JRP118 immunofluorescent signal for DAPI, MerTK, CD3, CD19, and CD90 (top), and detected cell subsynovial niche classifications (bottom). **C.** Bar chart of cell subsynovial niche proportions for each of the sections. **D.** Grouped dot plot of cell subsynovial niche abundances in each section as a percentage of total section detected cells, split between RA (red) and OA (blue). Each point represents one section. Error bars are standard error of the mean. The statistical test is a two-way ANOVA with multiple comparisons. ns = not significant. **E-G.** Pie chart of the cell type abundances in the pooled LL cells (**E**), LA cells (**F**), and SL cells (**G**).

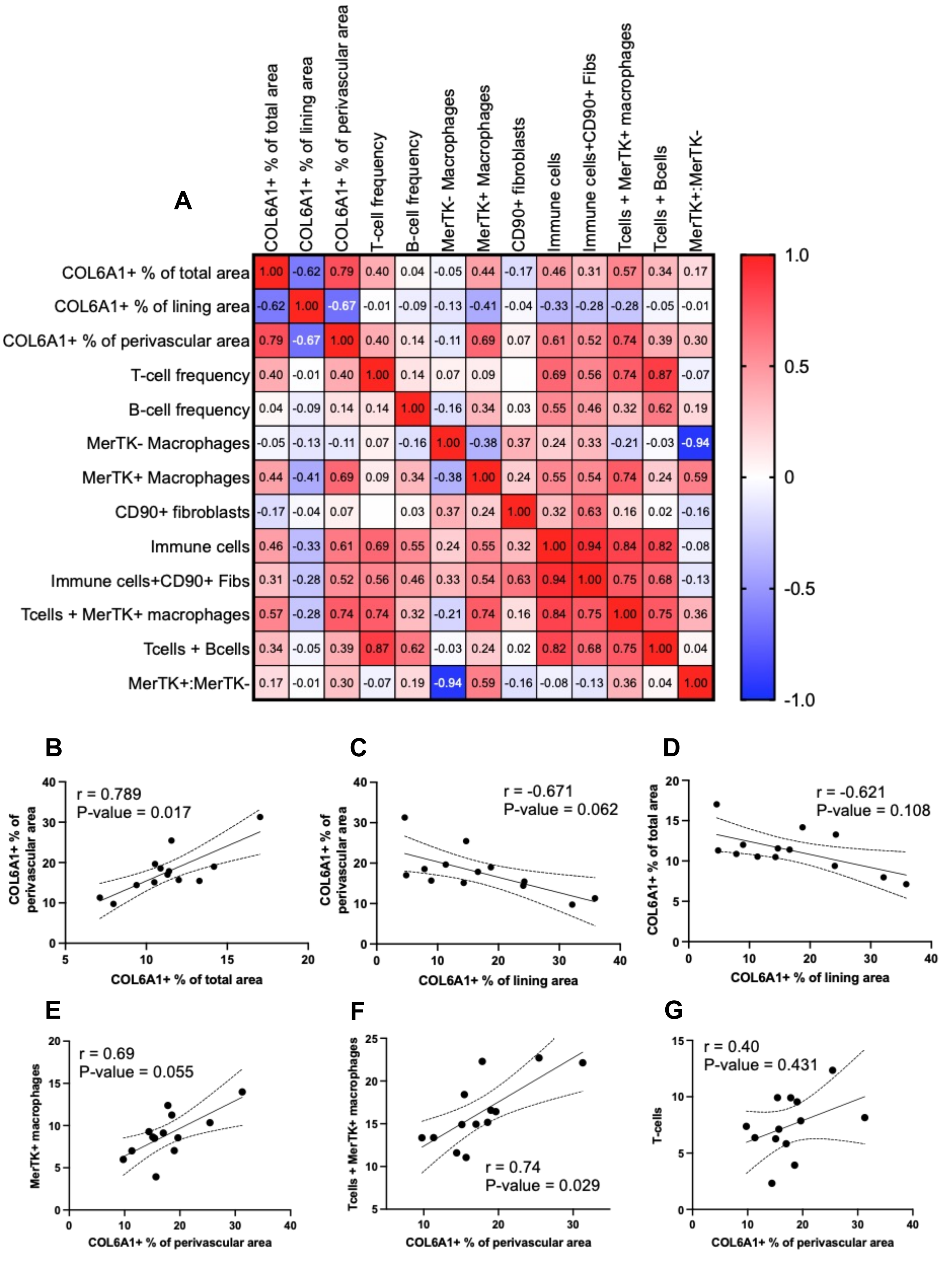

Appendix Figure S14. Correlations between COL6A1 niche positivity and cellular abundances.

Appendix Figure S14. Correlations between COL6A1 niche positivity and cellular abundances. **A.** Pearson's correlation coefficient matrix for all compared variables. **B.** Correlation between COL6A1-positive perivascular area and COL6A1-positive total tissue area. Each point represents one section. Line represents simple linear regression and dotted lines represent the 95% confidence interval. **C.** Correlation between COL6A1-positive perivascular area and COL6A1-positive lining layer area. **D.** Correlation between COL6A1-positive total tissue area and COL6A1-positive lining layer area. **E.** Correlation between total section MerTK+ macrophage frequency and COL6A1-positive perivascular area. **F.** Correlation between total section T-cell and MerTK+ macrophage frequency and COL6A1-positive perivascular area. **F.** Correlation between total section T-cell frequency and COL6A1-positive perivascular area, Rsq=Pearson correlation coefficient, P=P-value from two-tailed test, Benjamini-Hochberg adjusted.

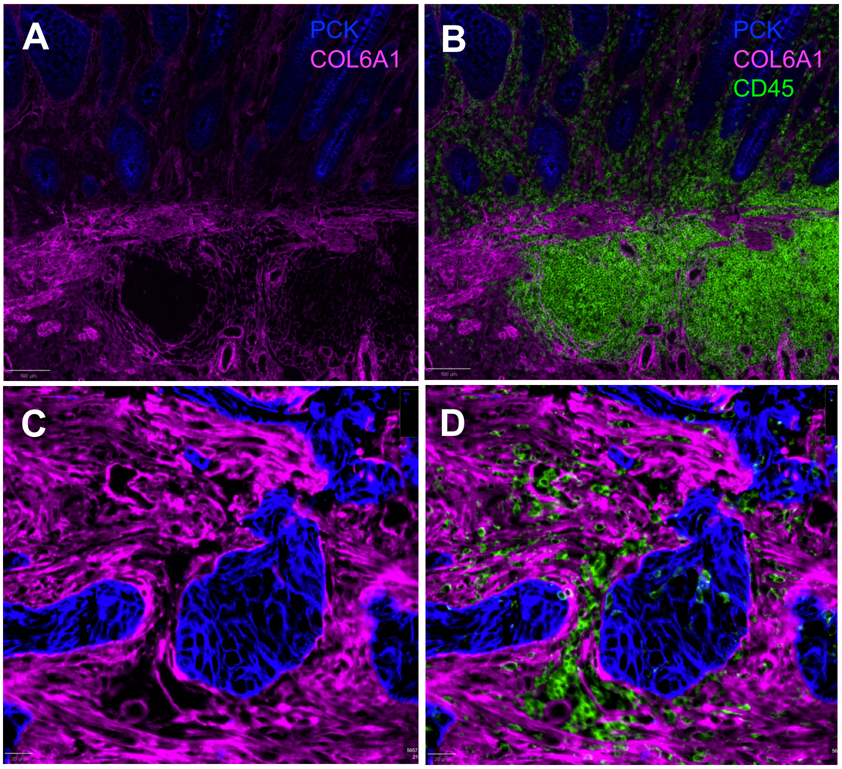

Appendix Figure S15. Collagen VI dark zones are present in IBD and head and neck cancer patients. Immunofluorescent staining was used to characterise the spatial distribution of immune cells in relation to COL6A1.QuPath was used to visualise immunofluorescent imaging of COL6A1 (magenta), PCK (blue), and CD45 (green) in colon section from Crohn's Disease patient (representative of n=5 sections), and head and neck cancer section (representative of n=6 sections). **A-B.** Visualisations of COL6A1, PCK+ epithelium, and CD45+ immune cells in Crohn's Disease colon section (representative of n=5 sections). **C-D.** Visualisations of COL6A1, PCK+ cancer cells, and CD45+ immune cells in head and neck squamous cell carcinoma sections (representative of n=8 sections).

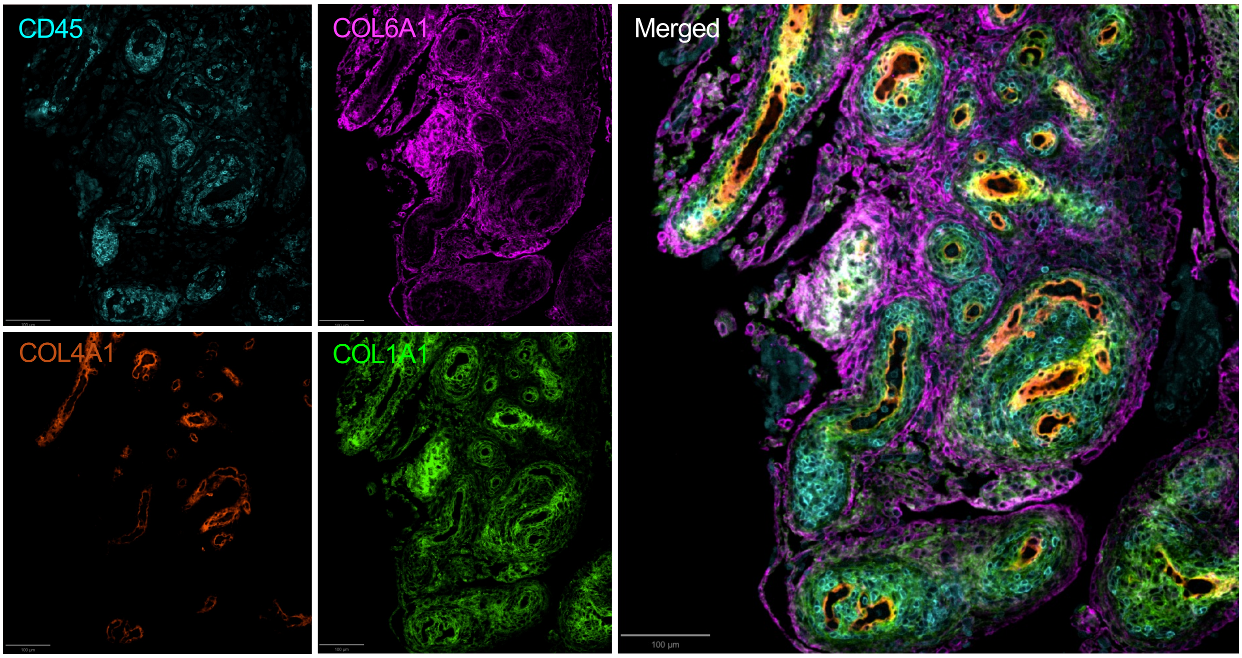

Appendix Figure S16. COL1A1 and COL6A1 display distinct deposition patterns in RA and OA. Immunofluorescent staining was used to characterise the spatial distribution of COL1A1 and COL6A1.QuPath was used to visualise immunofluorescent imaging of COL6A1 (magenta), CD45 (cyan), COL4A1 (orange), and COL1A1 (green) in section JRP141 (representative of 3 OA and 3 RA sections).

Appendix Table S1. Decision matrix for cell phenotyping as part of validation panel. Pos = positive, neg = negative.

		CD66b	MMP9	CD14	CD3	CD20	CD4	COL4A1	CD45	CD68	CD8	CD11c	MZBI	CD11b
**all**	**Other Immune cells**	anypos		anypos	anypos	anypos	anypos		anypos	anypos	anypos	anypos	anypos	anypos
**all**	**Non-immune cells**	allneg		allneg	allneg	allneg	allneg		allneg	allneg	allneg	allneg	allneg	allneg
**Non-immune cells**	**Vascular cells**							pos						
**Other Immune cells**	**Lymphoid cells**				anypos	anypos	anypos				anypos		anypos	
**Other Immune cells**	**Myeloid cells**	anypos	anypos	anypos						anypos		anypos		anypos
**Lymphoid cells**	**T cells**				anypos		anypos				anypos			
**Lymphoid cells**	**B cells**					pos								
**Lymphoid cells**	**Plasma cells**												pos	
**T cells**	**CD8 T cells**										anypos			
**T cells**	**CD4 T cells**						anypos							
**Myeloid cells**	**Neutrophils**	anypos	anypos	neg										
**Myeloid cells**	**Monocytes**			anypos						neg				
**Myeloid cells**	**Macrophages**			neg						anypos		neg		
**Myeloid cells**	**Dendritic cells**			allneg						allneg		anypos		
