# Supplementary material for: Synovial matrix turnover controls immune cell spatial patterning in inflammation resolution
Source: Mol Syst Biol. 2025 Sep 22;21(11):1638–65. doi: 10.1038/s44320-025-00149-7 (PMC12583461; doi:10.1038/s44320-025-00149-7)
Supplement: Supplementary file 8 — Source data Fig. 1 [file 44320_2025_149_MOESM8_ESM.zip › Figure 1/1B-G/README.rtf]

EG = ECM GlycoproteinC = Collagen enrichmentPG= ProteoglycanEAP = ECM-associated proteinER = ECM RegulatorSF = Secreted Factor
