# Supplementary figures and images for: Synovial matrix turnover controls immune cell spatial patterning in inflammation resolution
Source: Mol Syst Biol. 2025 Sep 22;21(11):1638–65. doi: 10.1038/s44320-025-00149-7 (PMC12583461; doi:10.1038/s44320-025-00149-7)

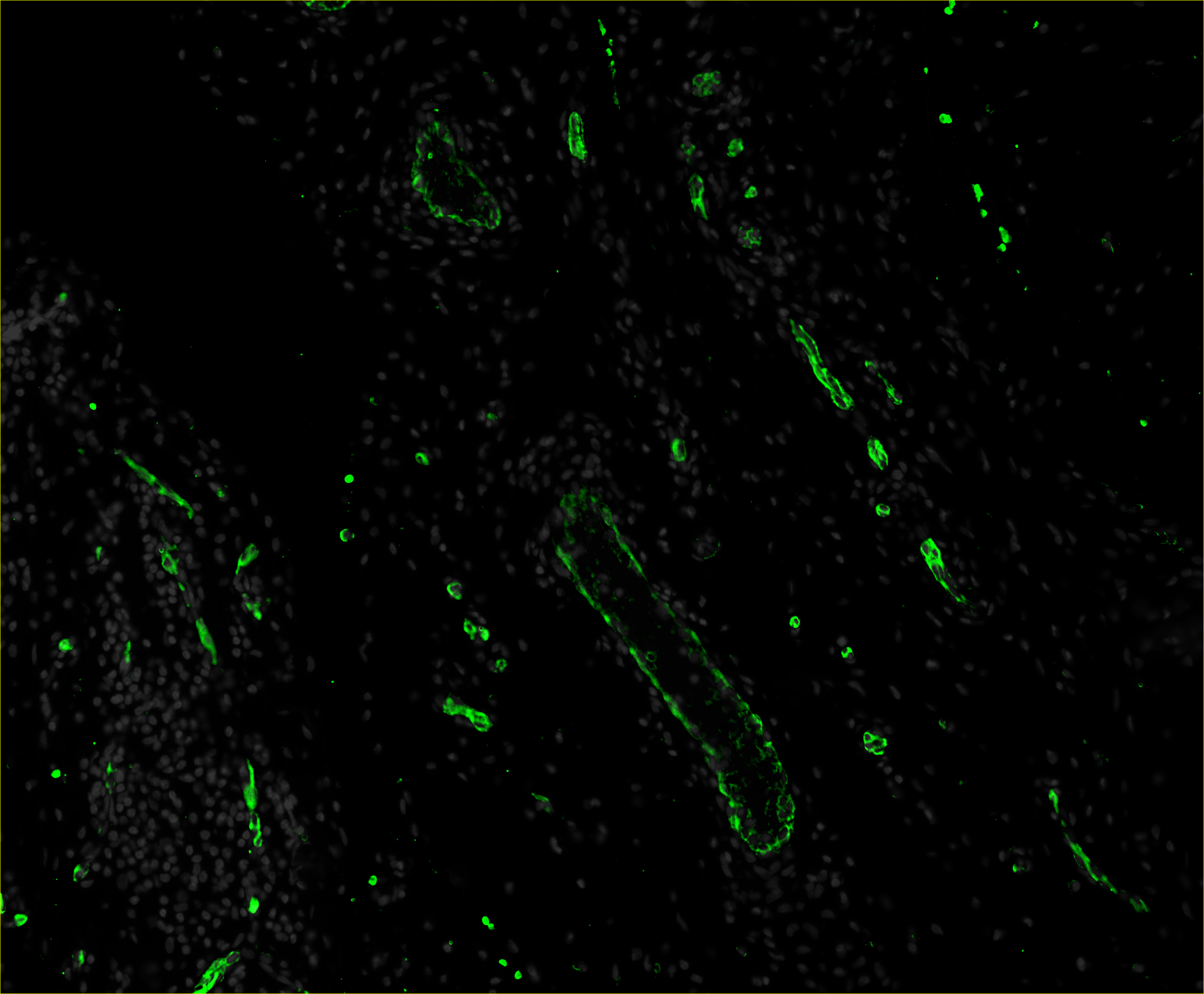

Supplement: Supplementary file 9 — Source data Fig. 2 [file 44320_2025_149_MOESM9_ESM.zip › Figure 2/2E/JPR139d_IGFBP7_Fig2 (1, x=24065, y=30587, w=2721, h=2247).tif]

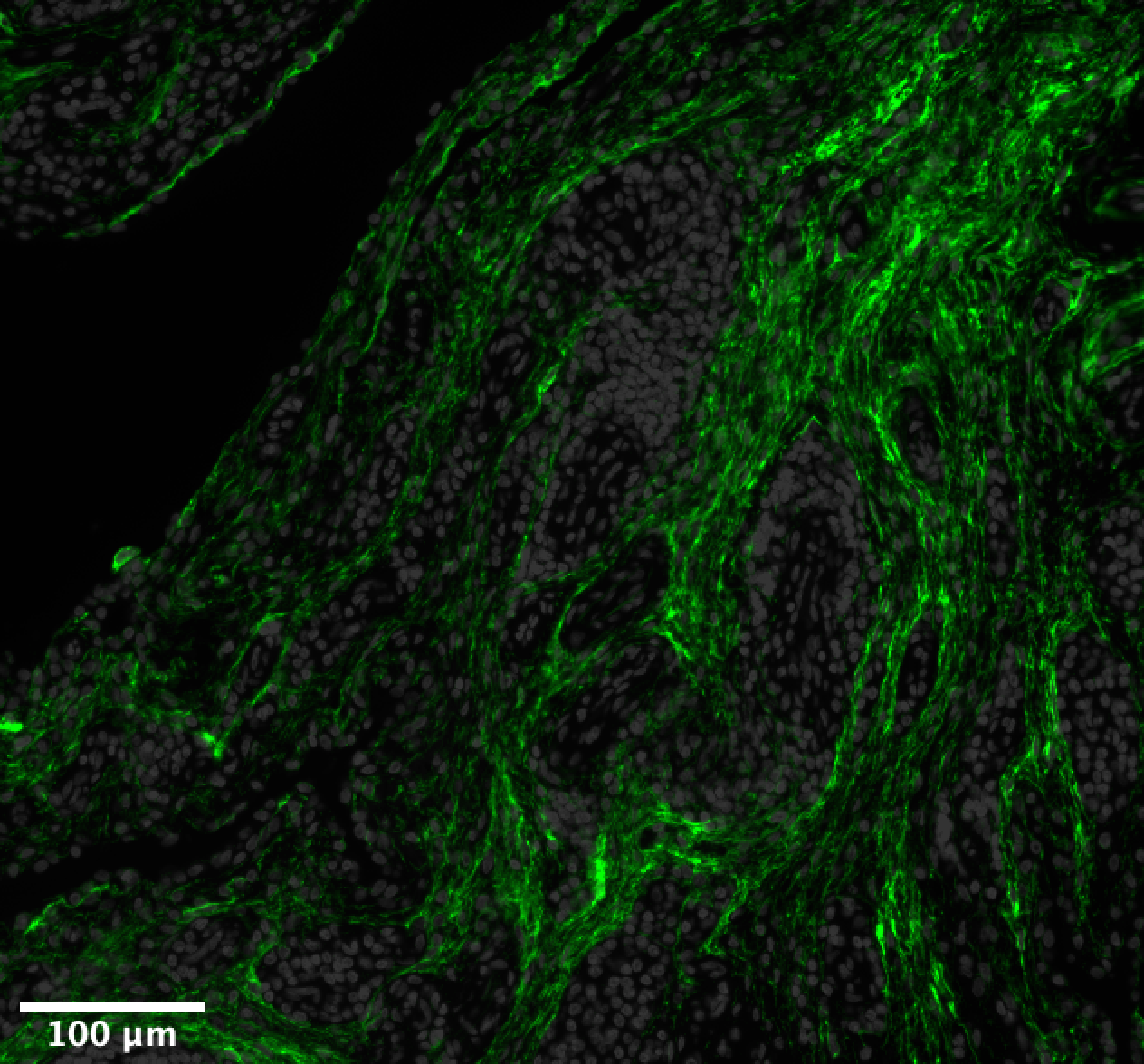

Supplement: Supplementary file 9 — Source data Fig. 2 [file 44320_2025_149_MOESM9_ESM.zip › Figure 2/2E/JRP122_MFAP5_Fig2 (1, x=3731, y=8887, w=1902, h=1775).png]

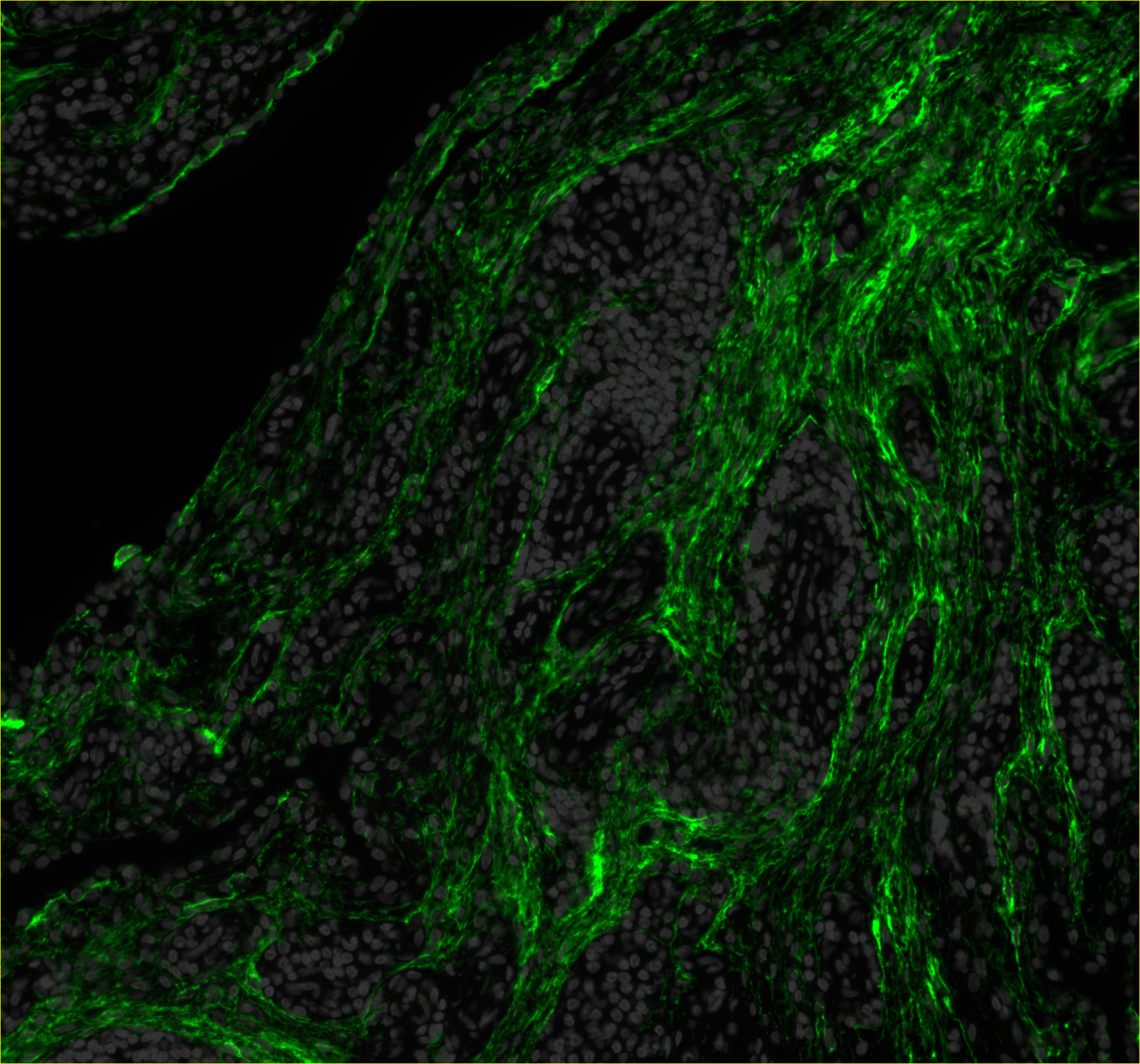

Supplement: Supplementary file 9 — Source data Fig. 2 [file 44320_2025_149_MOESM9_ESM.zip › Figure 2/2E/JRP122_MFAP5_Fig2 (1, x=3731, y=8887, w=1902, h=1775).tif]

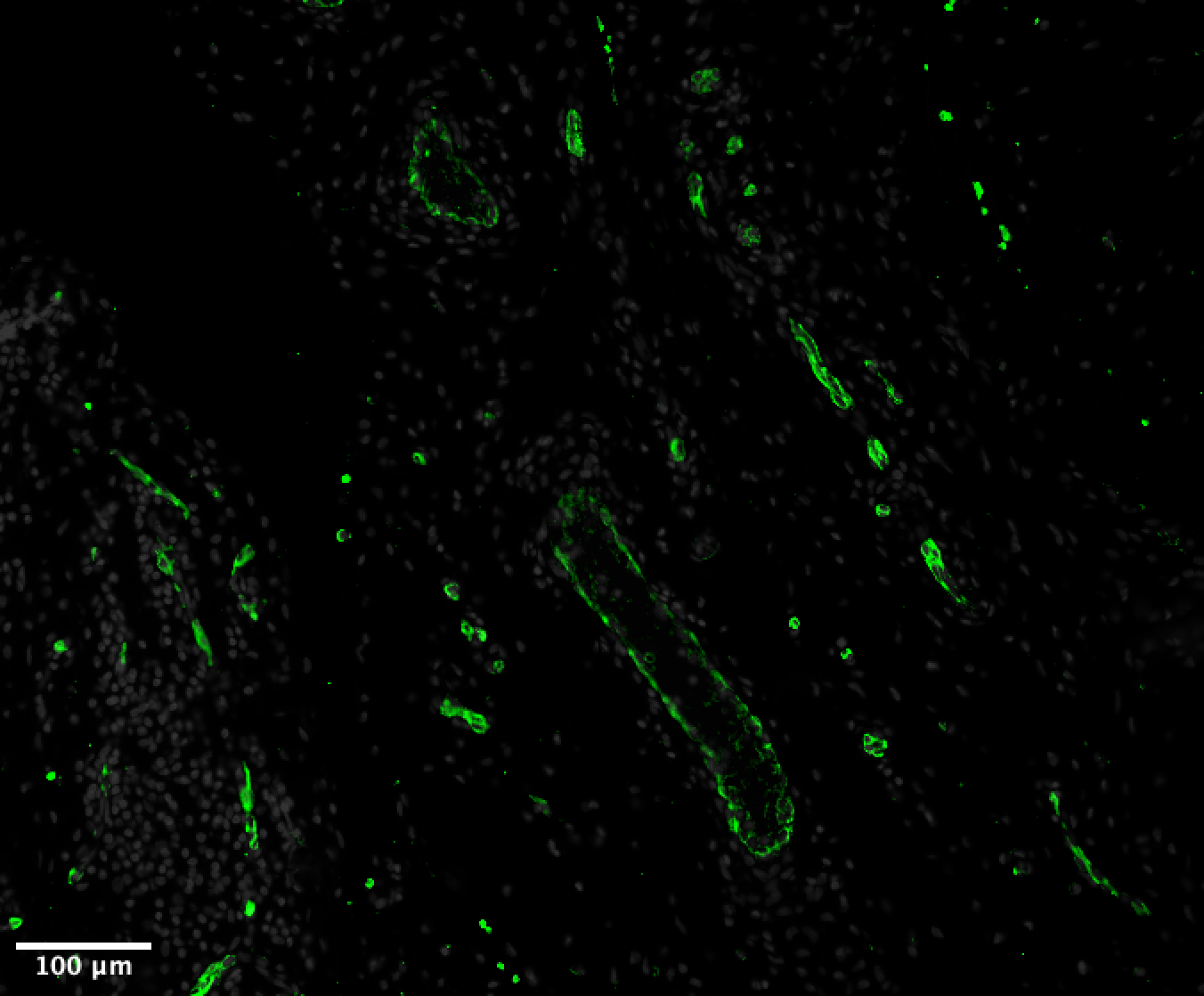

Supplement: Supplementary file 9 — Source data Fig. 2 [file 44320_2025_149_MOESM9_ESM.zip › Figure 2/2E/JPR139d_IGFBP7_Fig2 (1, x=24065, y=30587, w=2721, h=2247).png]

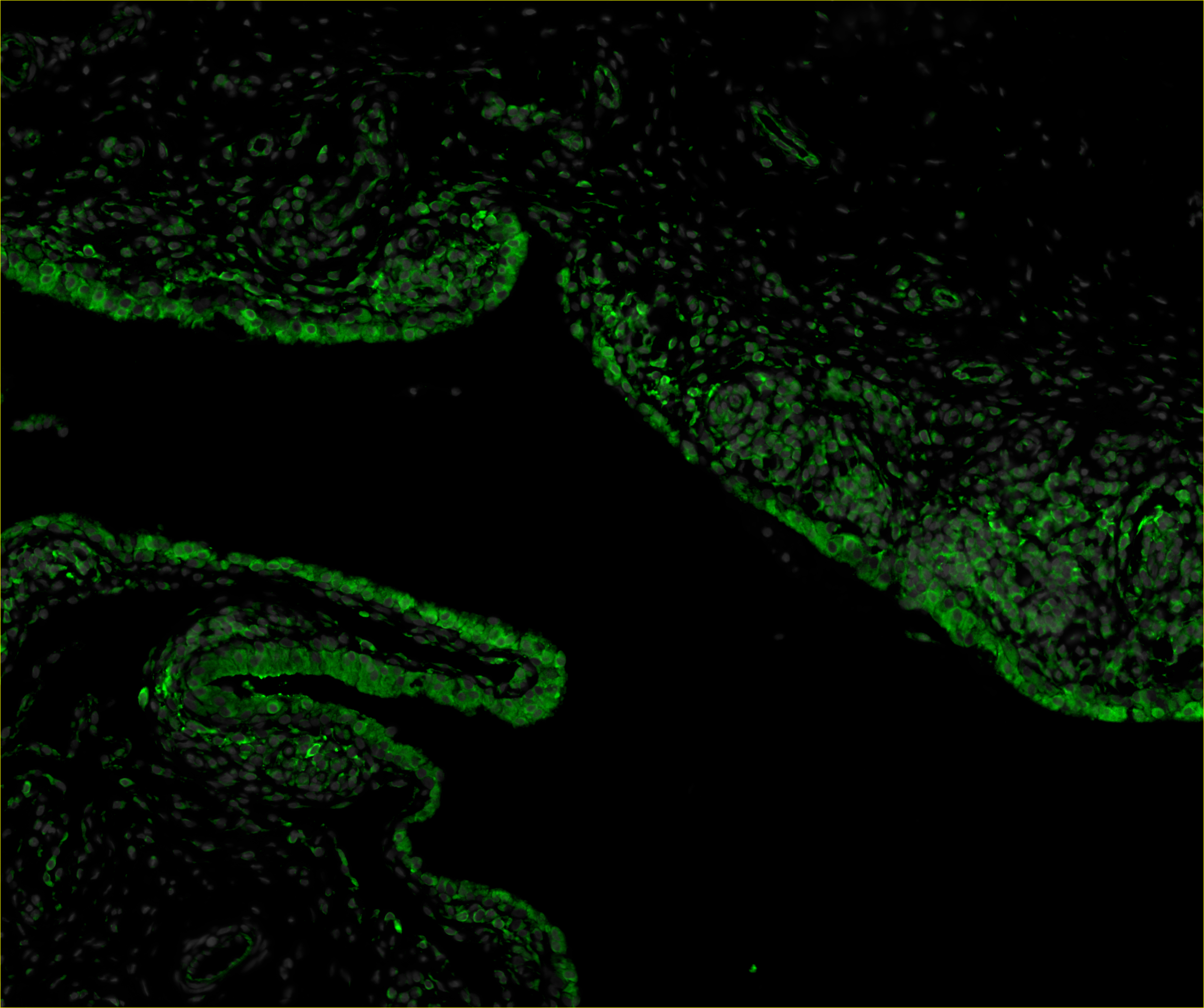

Supplement: Supplementary file 9 — Source data Fig. 2 [file 44320_2025_149_MOESM9_ESM.zip › Figure 2/2E/JPR122_IGFBP5_Fig2 (1, x=14448, y=9039, w=2567, h=2149).tif]

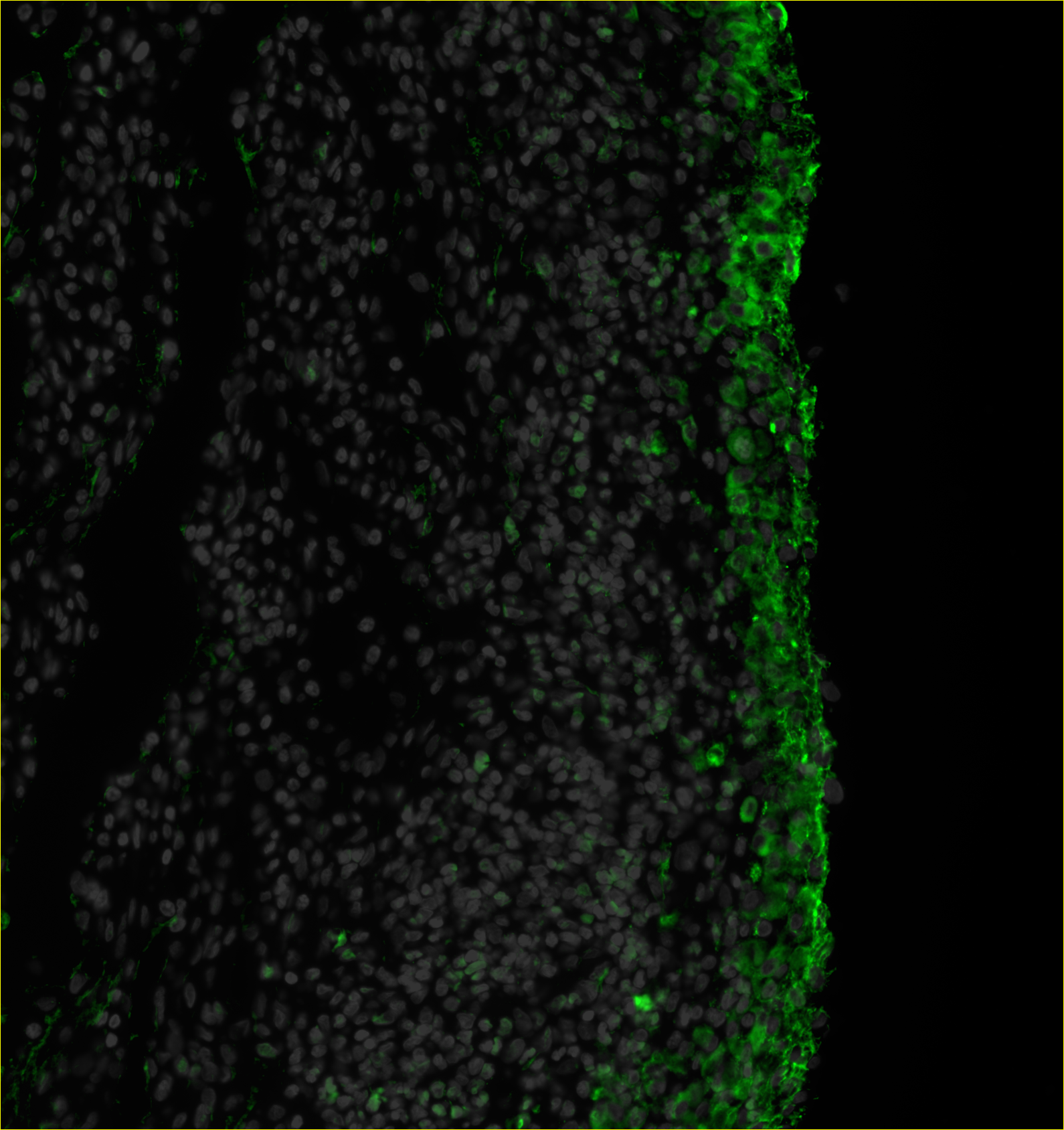

Supplement: Supplementary file 9 — Source data Fig. 2 [file 44320_2025_149_MOESM9_ESM.zip › Figure 2/2E/JRP112_FN_Fig2 (1, x=7109, y=9882, w=1590, h=1688).tif]

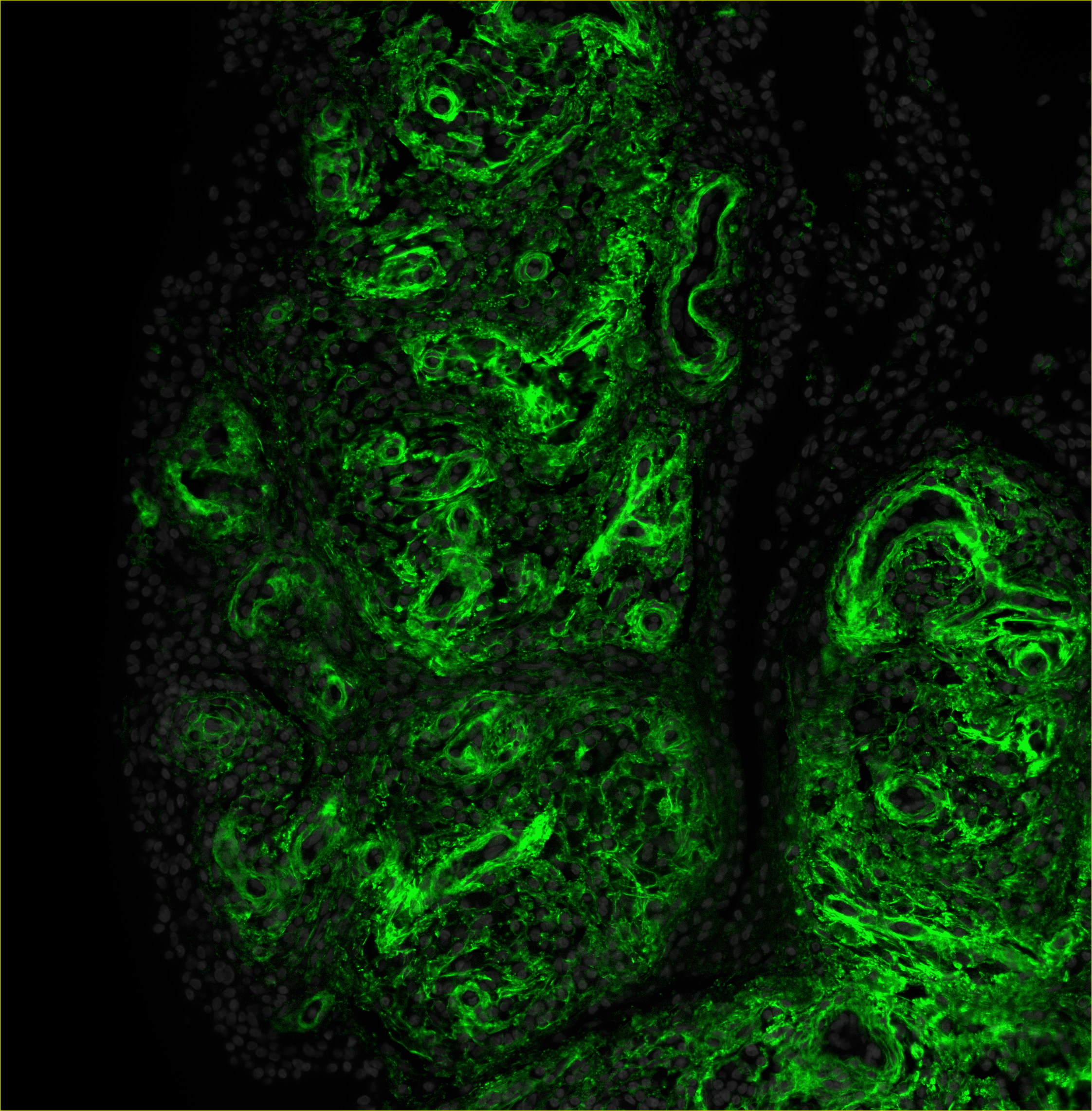

Supplement: Supplementary file 9 — Source data Fig. 2 [file 44320_2025_149_MOESM9_ESM.zip › Figure 2/2E/JRP122_COL1A1_Fig2 (1, x=1638, y=3464, w=2244, h=2283).tif]

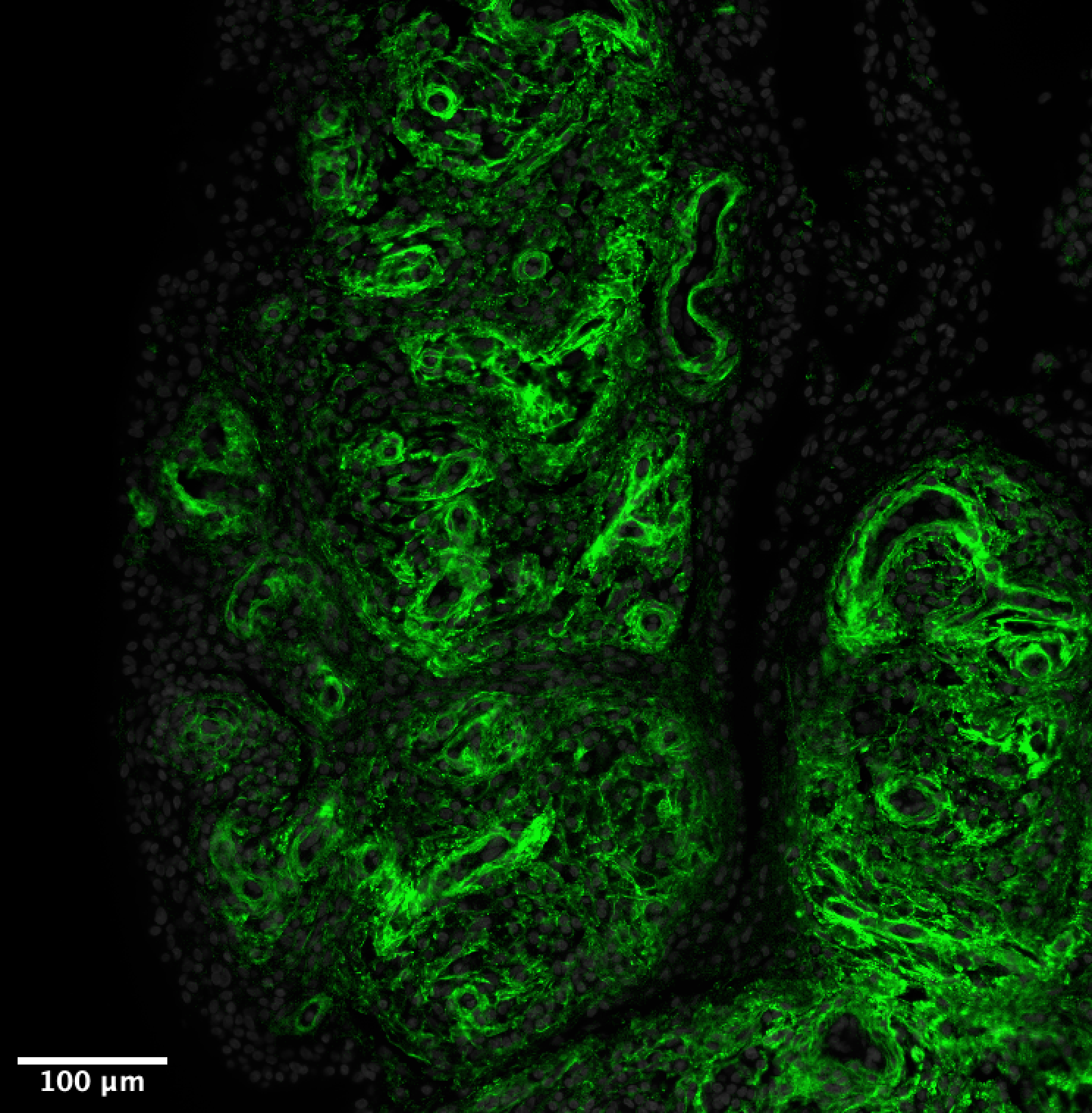

Supplement: Supplementary file 9 — Source data Fig. 2 [file 44320_2025_149_MOESM9_ESM.zip › Figure 2/2E/JRP122_COL1A1_Fig2 (1, x=1638, y=3464, w=2244, h=2283).png]

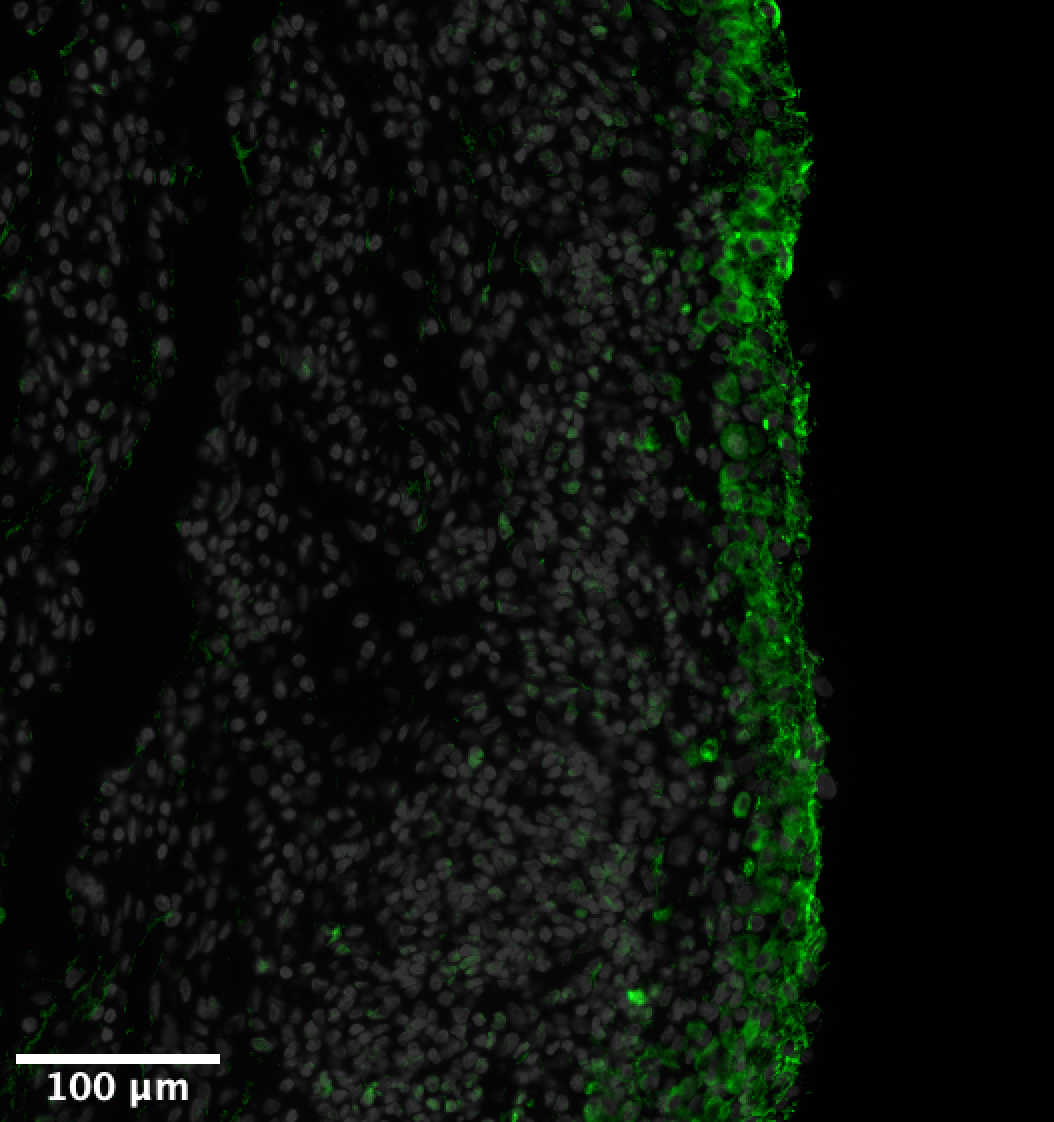

Supplement: Supplementary file 9 — Source data Fig. 2 [file 44320_2025_149_MOESM9_ESM.zip › Figure 2/2E/JRP112_FN_Fig2 (1, x=7109, y=9882, w=1590, h=1688).png]

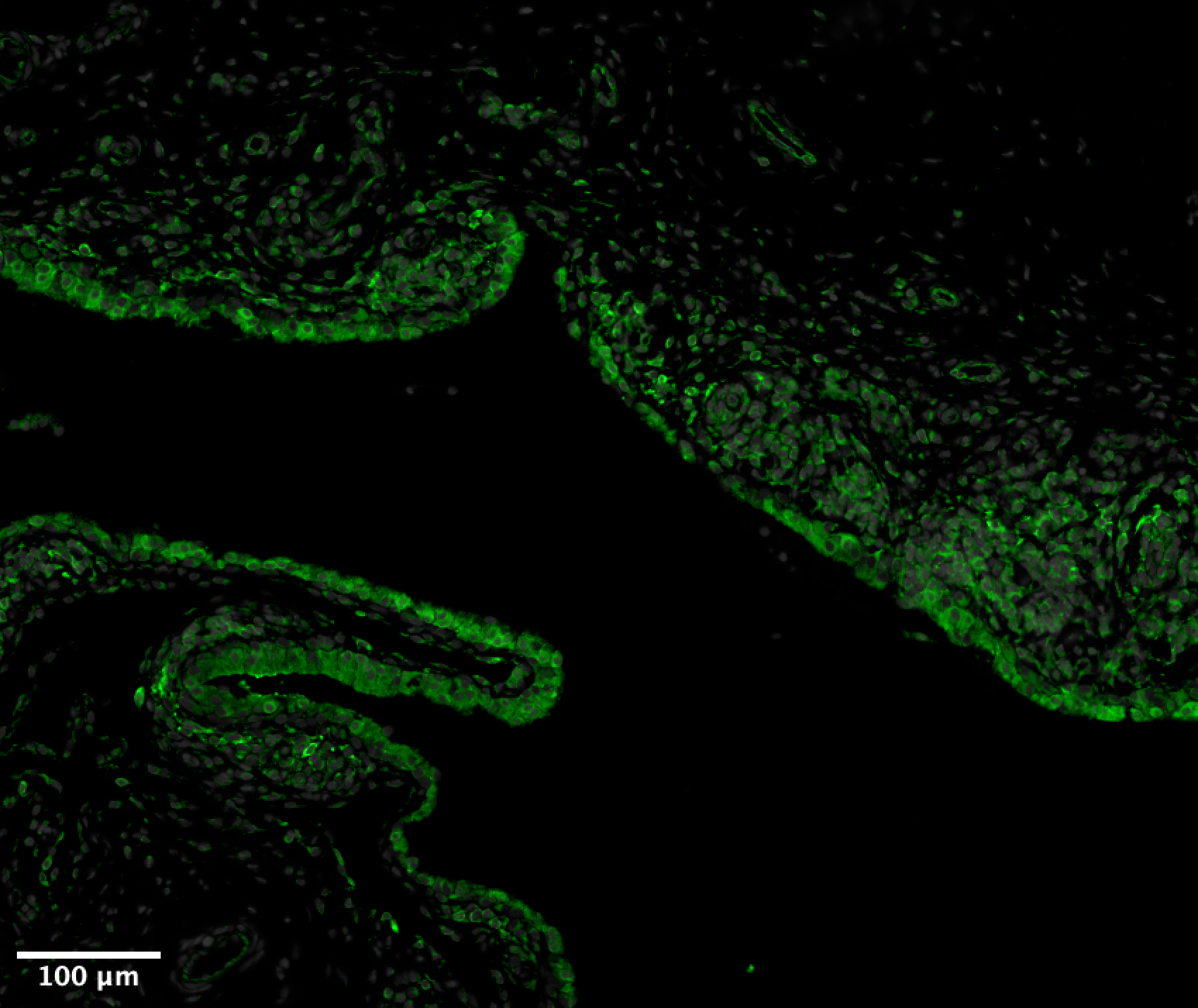

Supplement: Supplementary file 9 — Source data Fig. 2 [file 44320_2025_149_MOESM9_ESM.zip › Figure 2/2E/JPR122_IGFBP5_Fig2 (1, x=14448, y=9039, w=2567, h=2149).png]

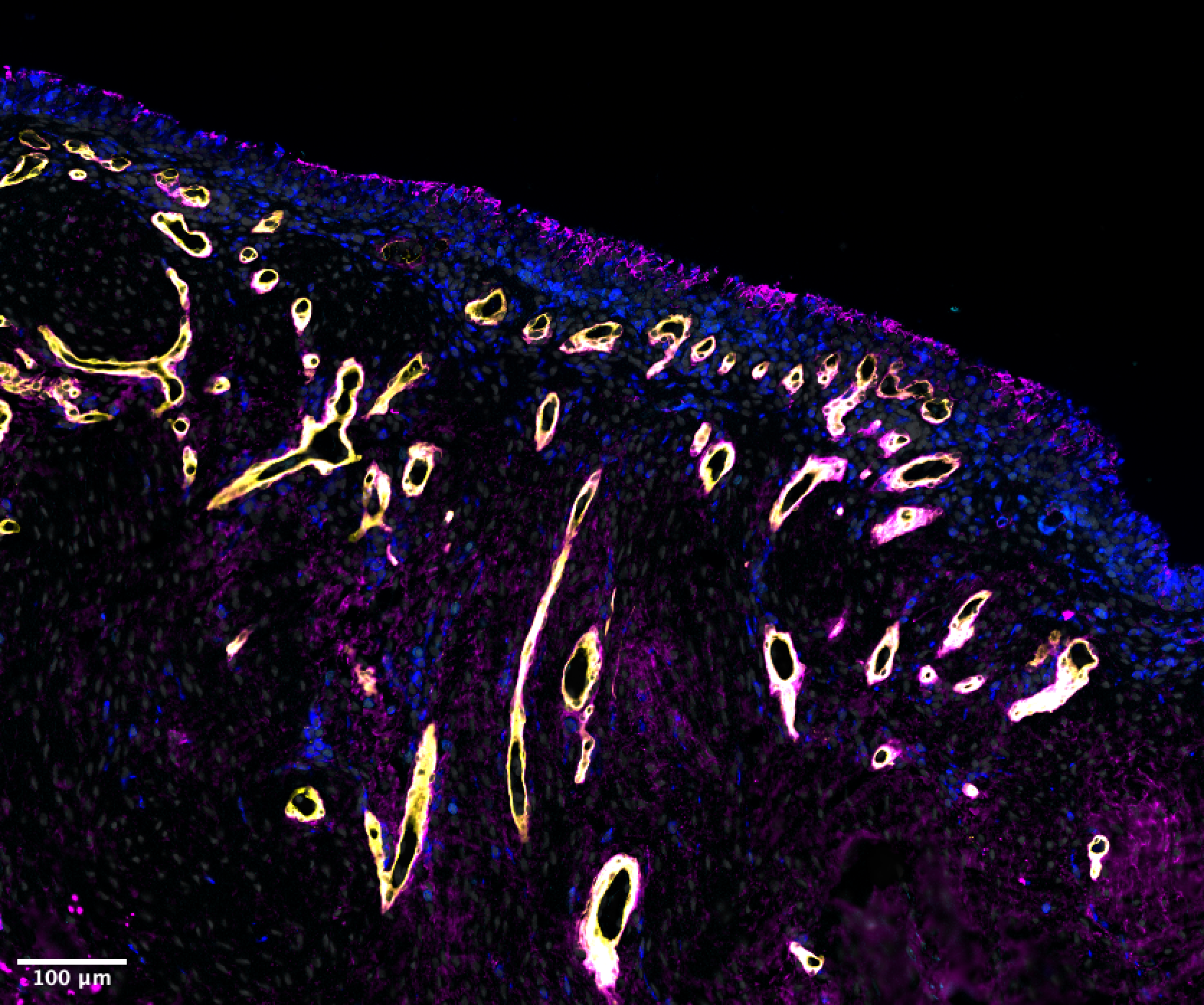

Supplement: Supplementary file 12 — Source data Fig. 5 [file 44320_2025_149_MOESM12_ESM.zip › Figure 5/5A/JPR099_LowRA_Fig5 (1, x=14509, y=963, w=3369, h=2806).png]

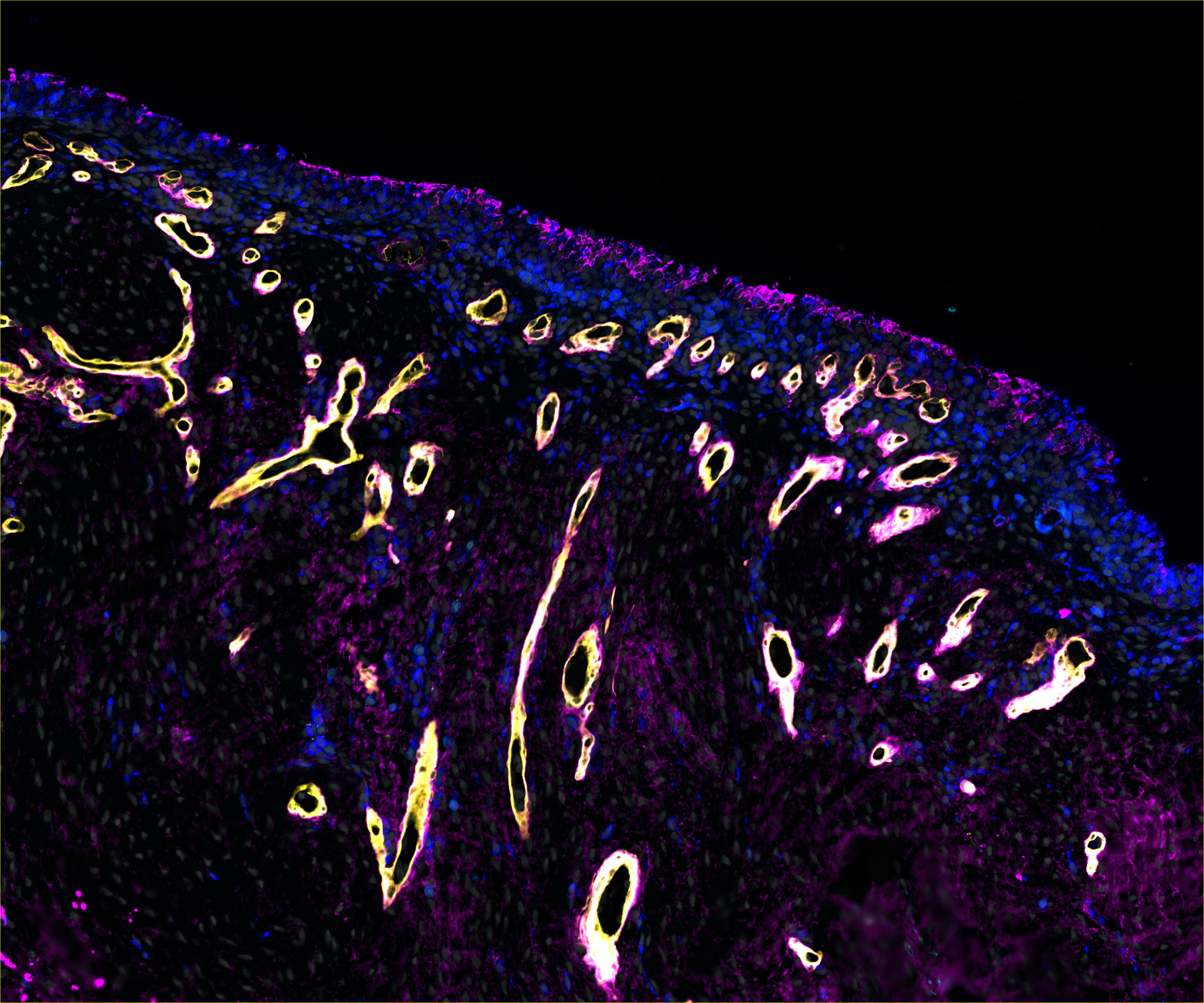

Supplement: Supplementary file 12 — Source data Fig. 5 [file 44320_2025_149_MOESM12_ESM.zip › Figure 5/5A/JPR099_LowRA_Fig5 (1, x=14509, y=963, w=3369, h=2806).tif]

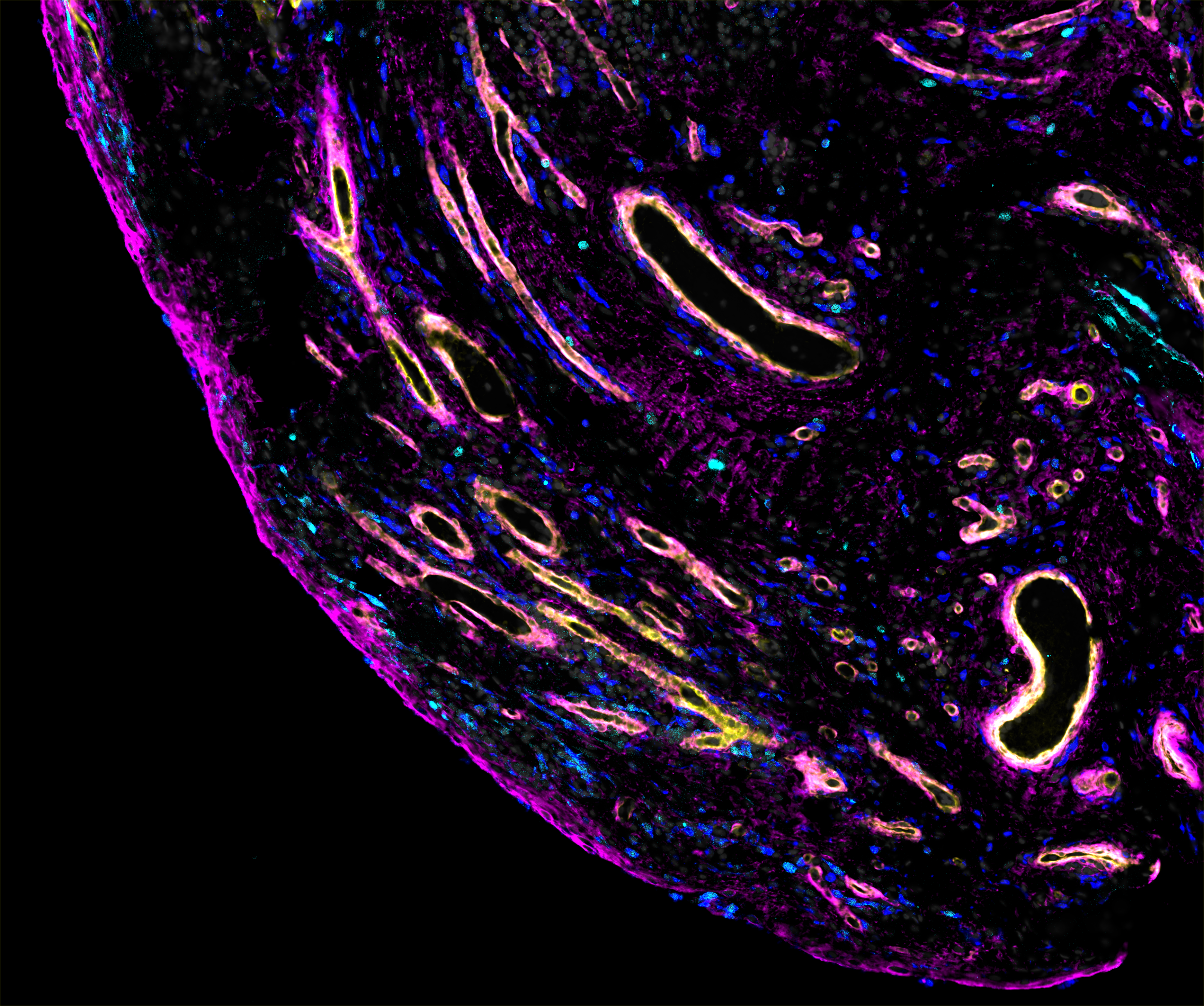

Supplement: Supplementary file 12 — Source data Fig. 5 [file 44320_2025_149_MOESM12_ESM.zip › Figure 5/5A/JPR139d_MidRA_Fig5 (1, x=1442, y=24880, w=3477, h=2906).tif]

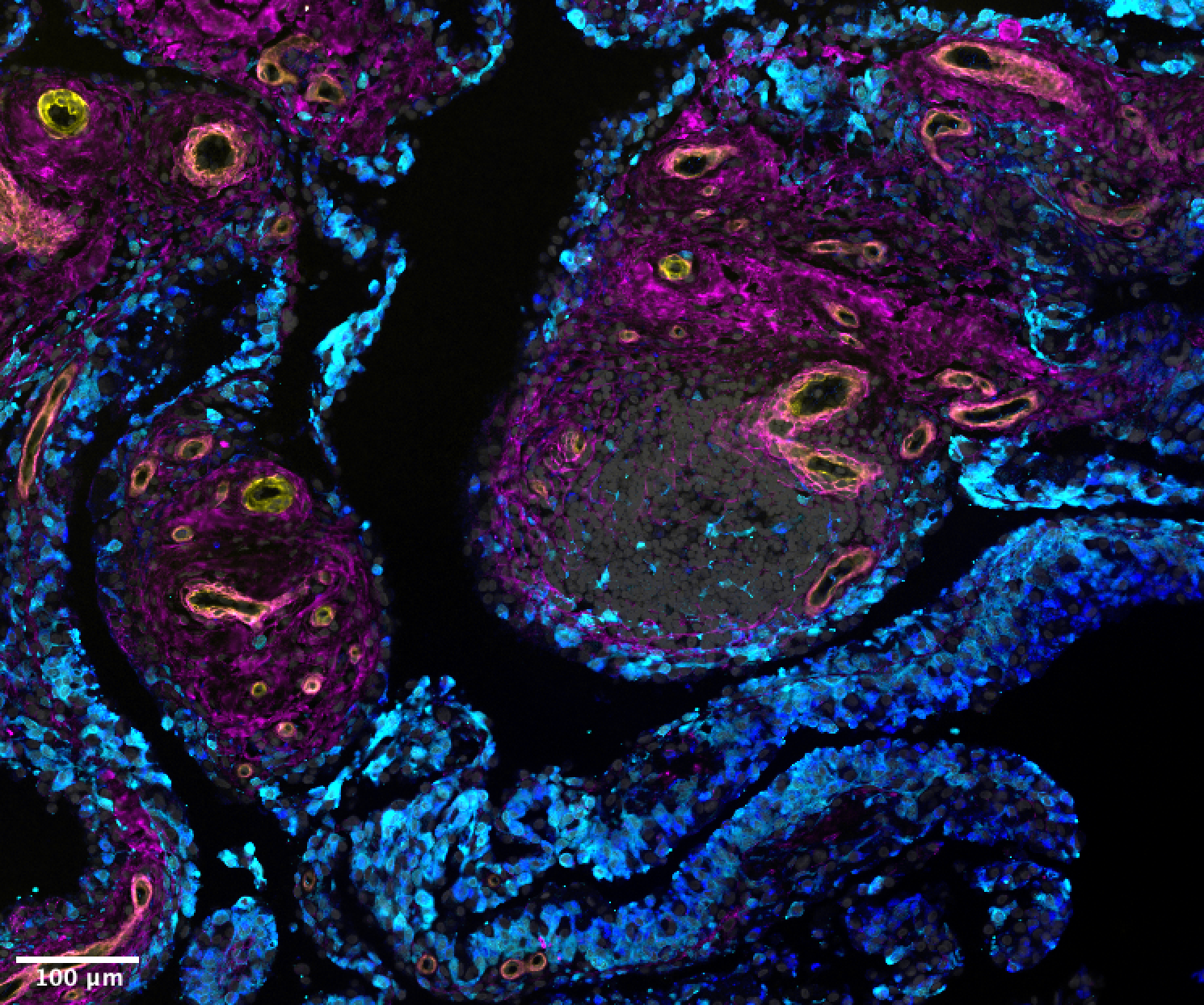

Supplement: Supplementary file 12 — Source data Fig. 5 [file 44320_2025_149_MOESM12_ESM.zip › Figure 5/5A/JRP118_HighRA_Fig5 (1, x=4737, y=17162, w=3000, h=2505).png]

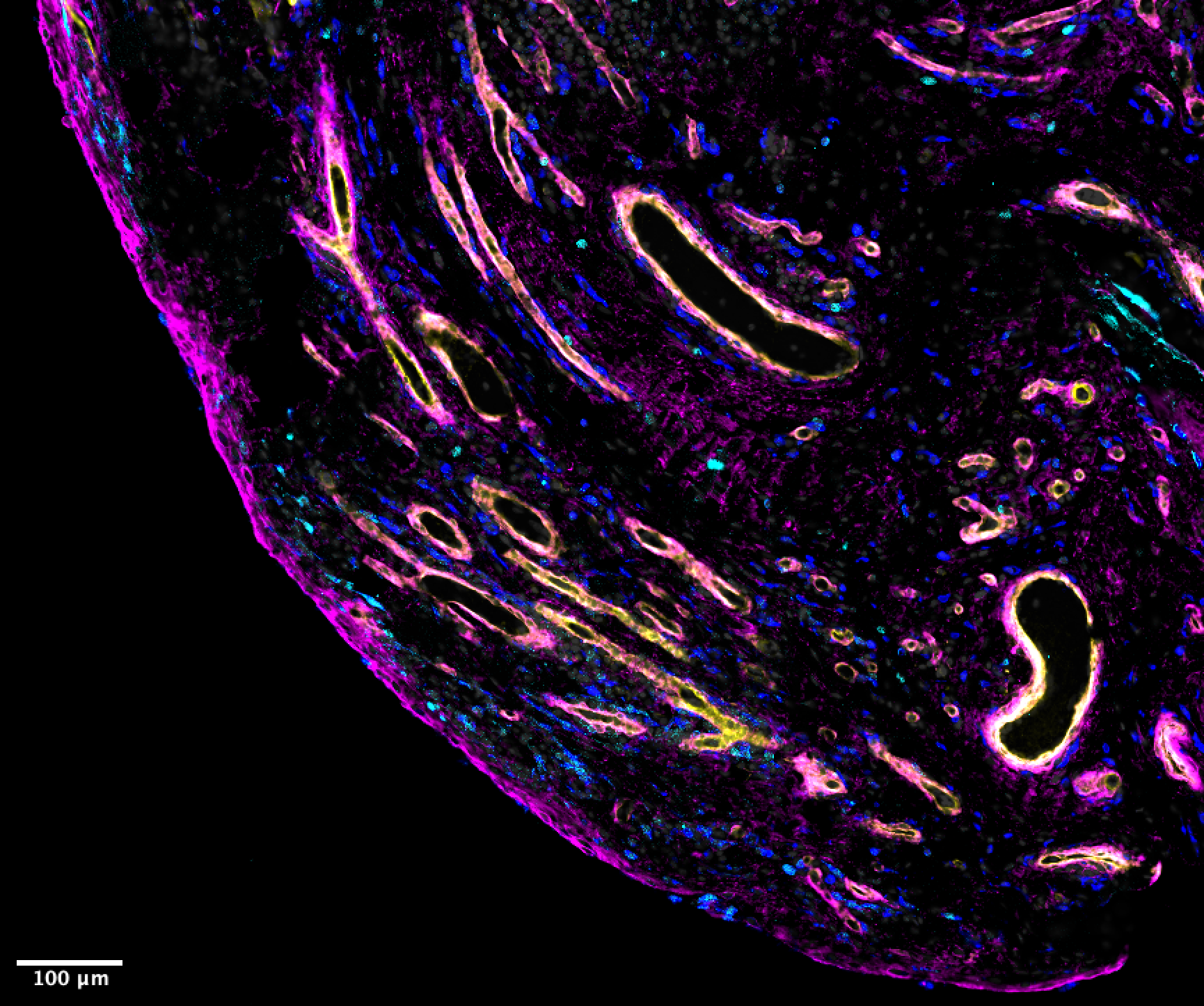

Supplement: Supplementary file 12 — Source data Fig. 5 [file 44320_2025_149_MOESM12_ESM.zip › Figure 5/5A/JPR139d_MidRA_Fig5 (1, x=1442, y=24880, w=3477, h=2906).png]

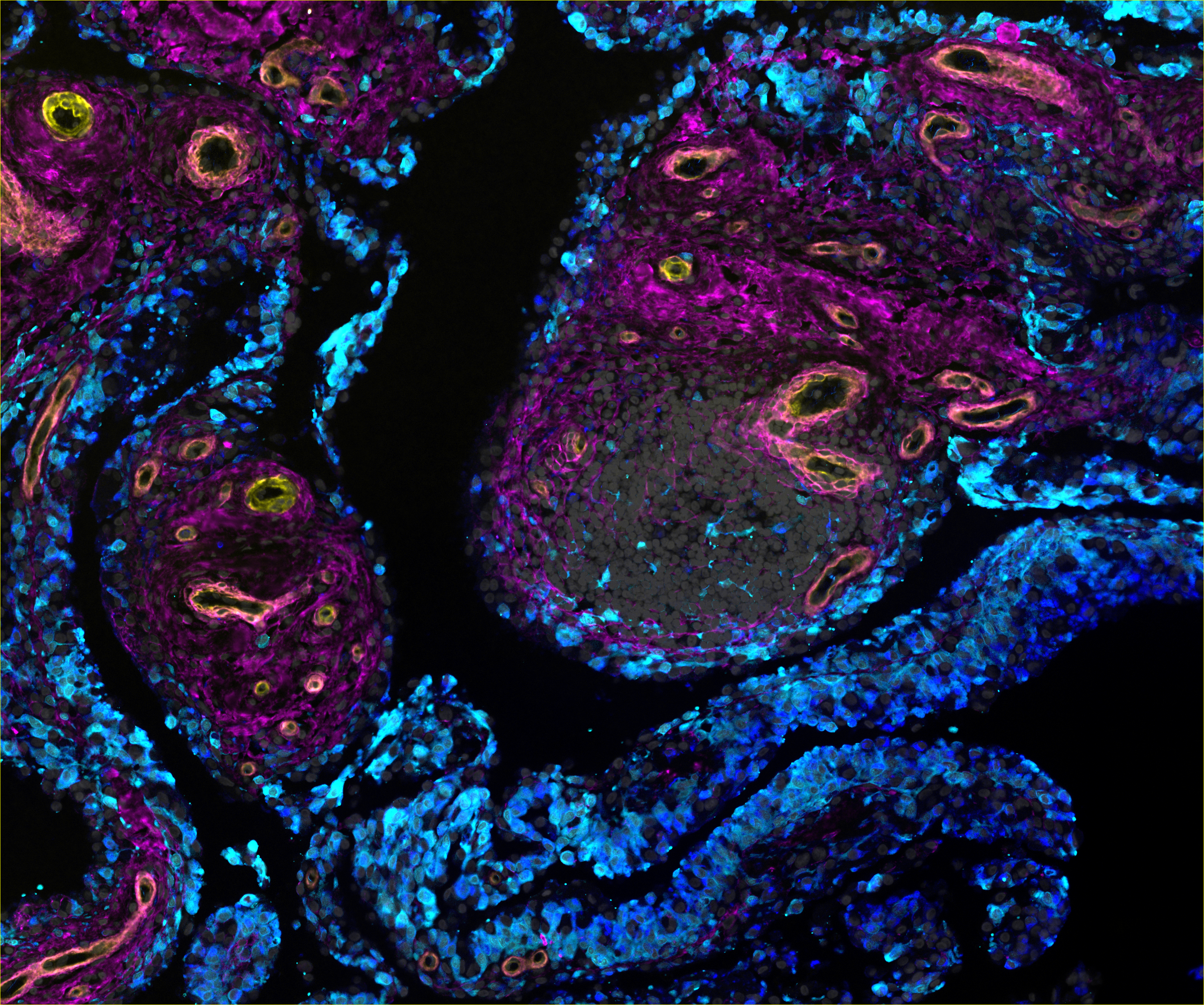

Supplement: Supplementary file 12 — Source data Fig. 5 [file 44320_2025_149_MOESM12_ESM.zip › Figure 5/5A/JRP118_HighRA_Fig5 (1, x=4737, y=17162, w=3000, h=2505).tif]

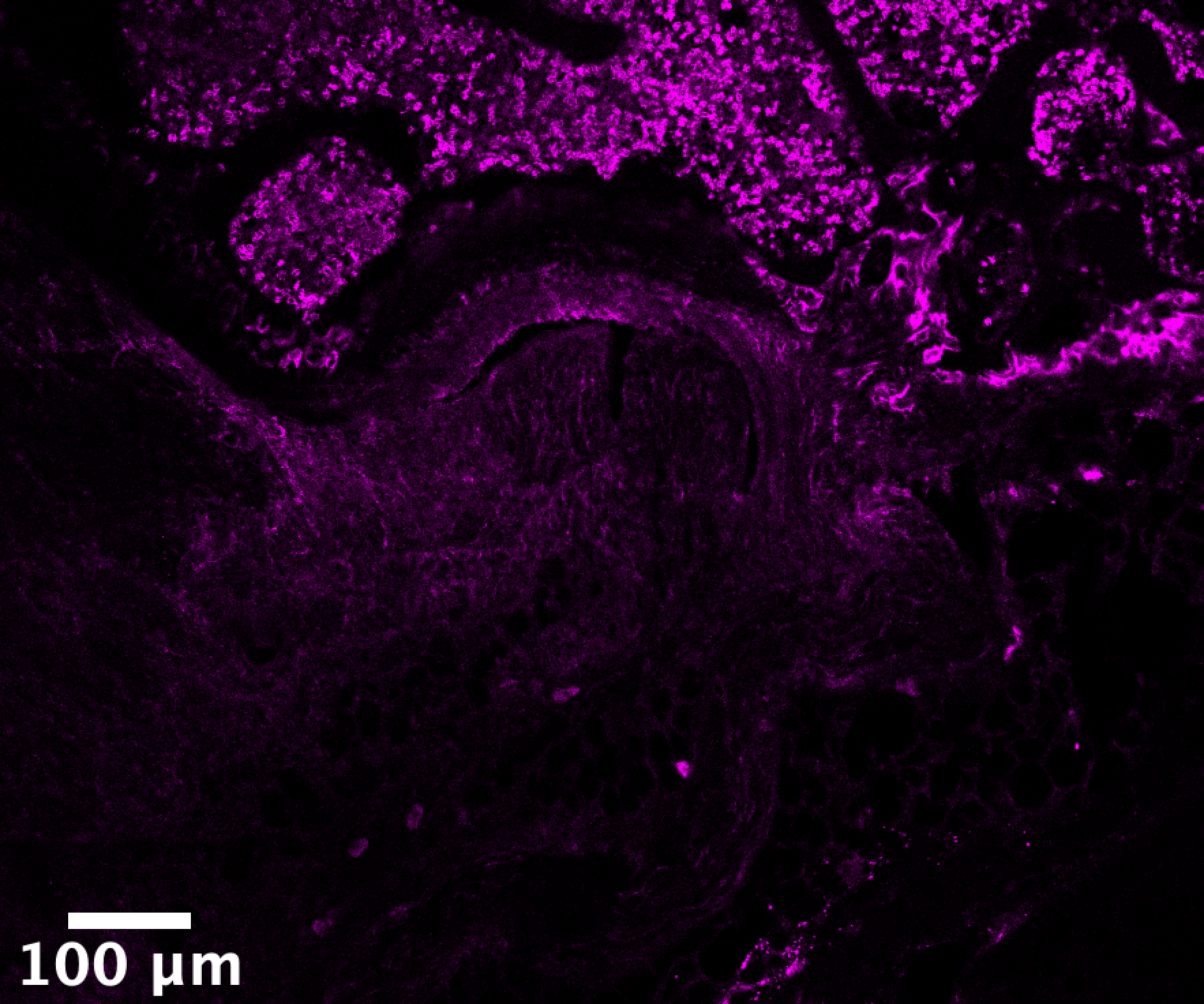

Supplement: Supplementary file 12 — Source data Fig. 5 [file 44320_2025_149_MOESM12_ESM.zip › Figure 5/5C/Day14_COL6.png]

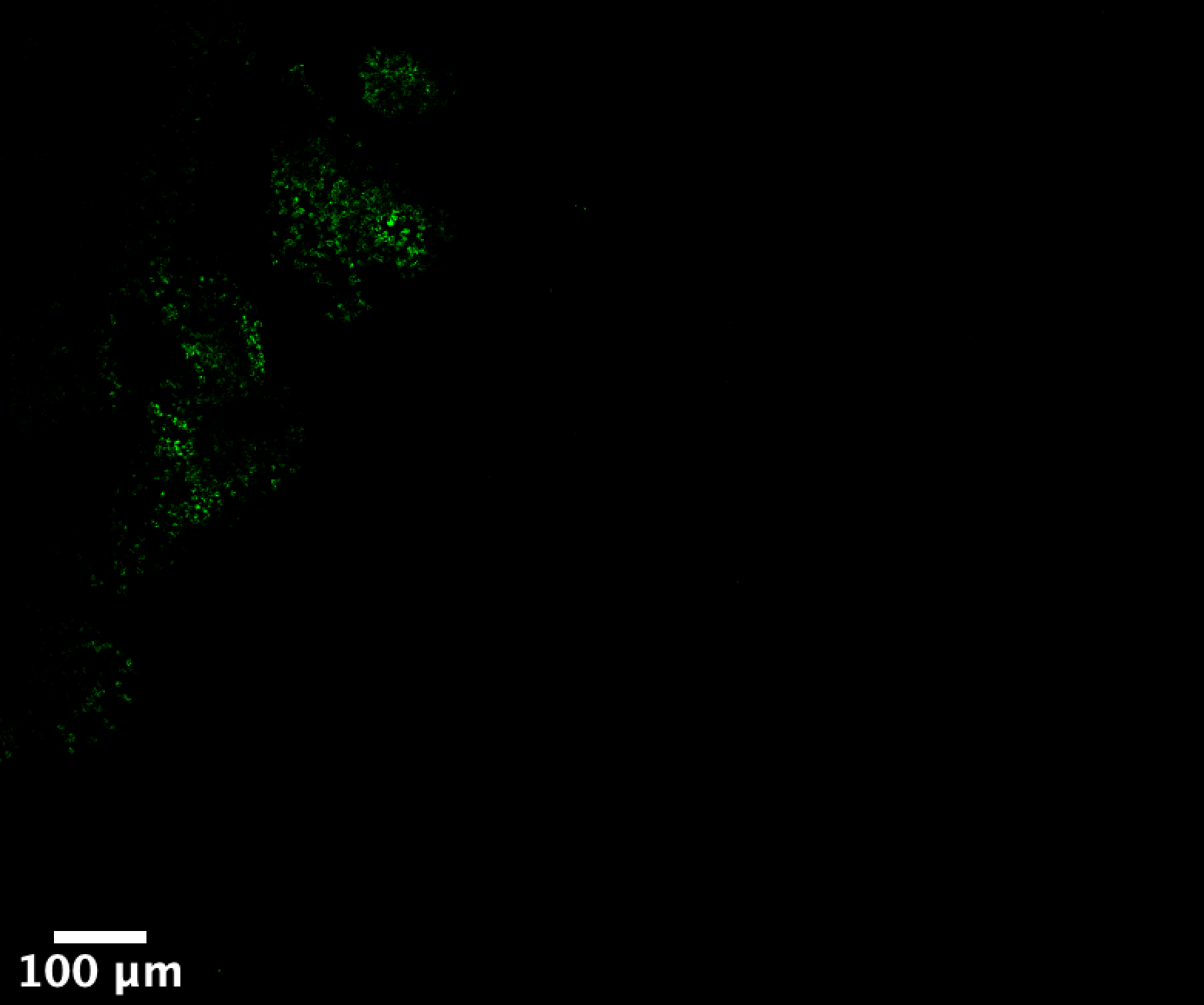

Supplement: Supplementary file 12 — Source data Fig. 5 [file 44320_2025_149_MOESM12_ESM.zip › Figure 5/5C/Naive_CD177.png]

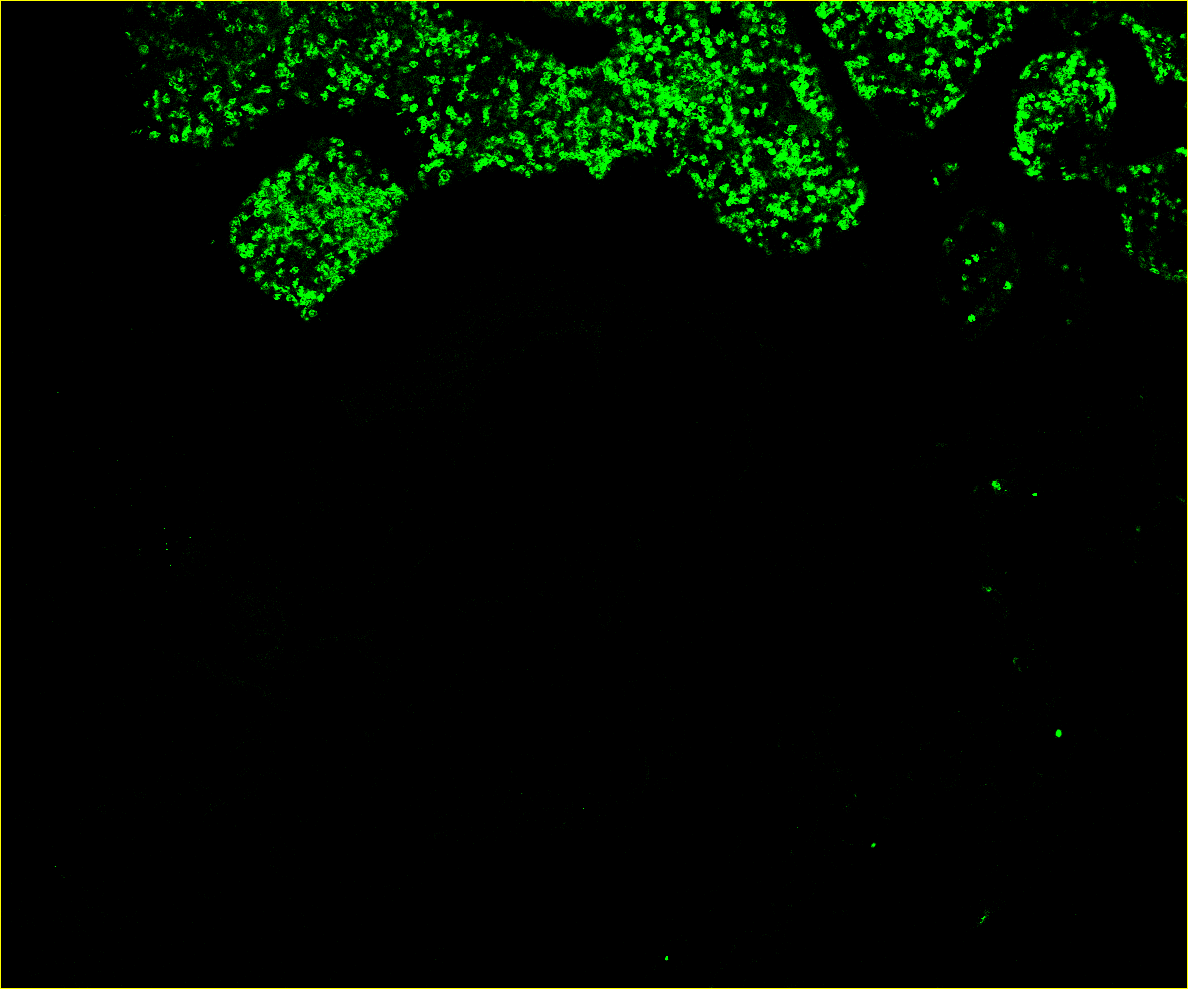

Supplement: Supplementary file 12 — Source data Fig. 5 [file 44320_2025_149_MOESM12_ESM.zip › Figure 5/5C/Day14_CD177.tif]

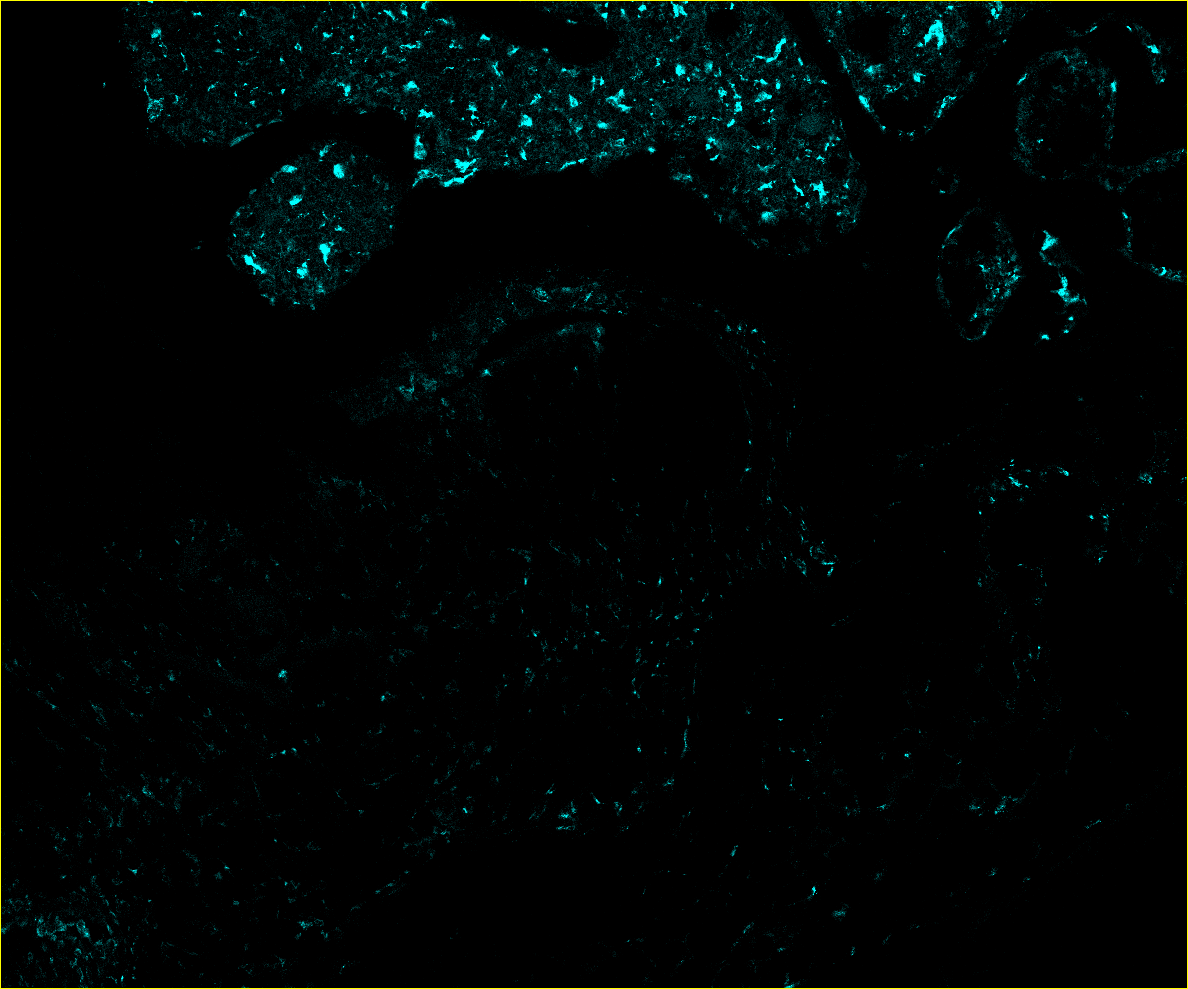

Supplement: Supplementary file 12 — Source data Fig. 5 [file 44320_2025_149_MOESM12_ESM.zip › Figure 5/5C/Day14_CD68.tif]

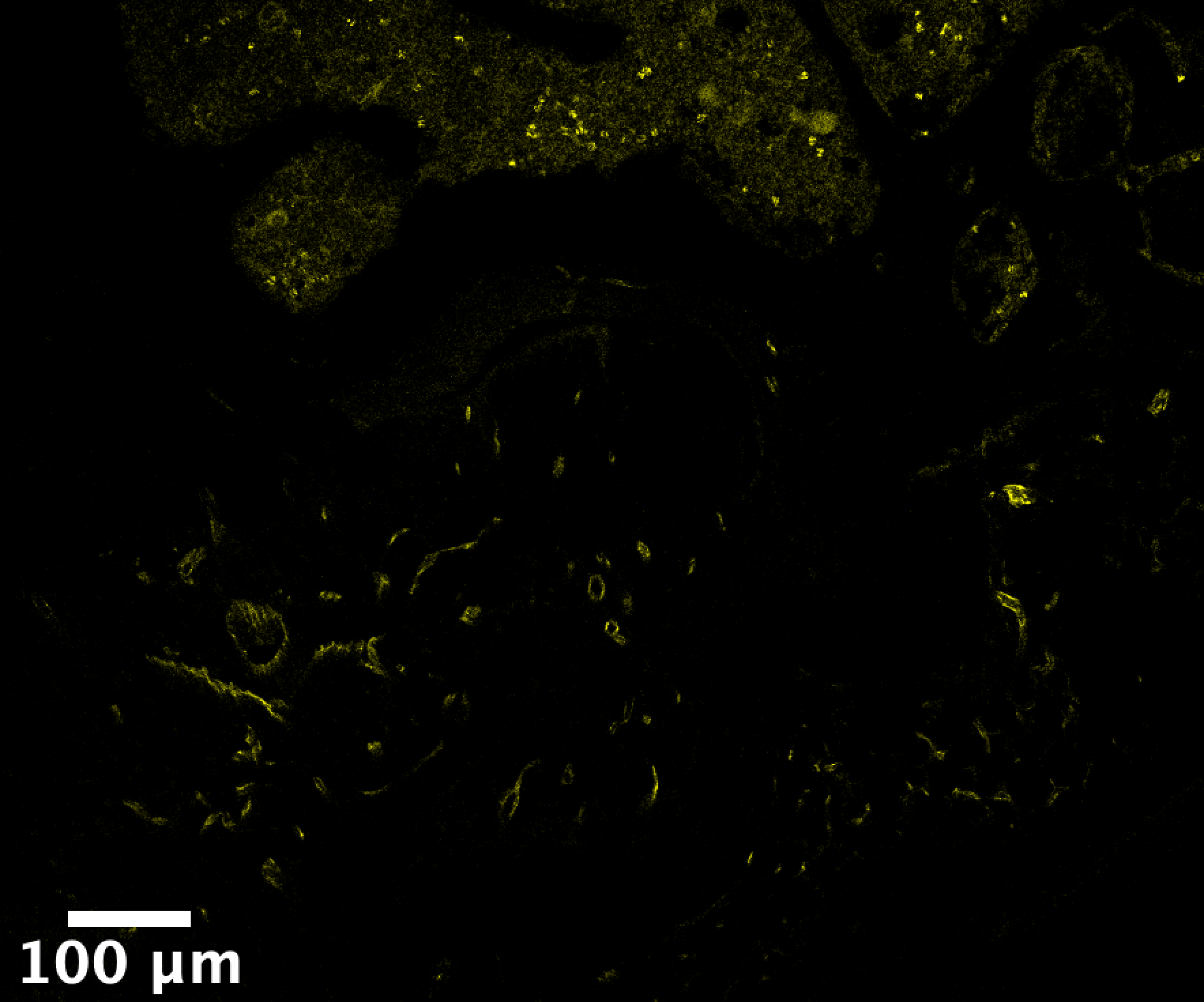

Supplement: Supplementary file 12 — Source data Fig. 5 [file 44320_2025_149_MOESM12_ESM.zip › Figure 5/5C/Day14_CD31.png]

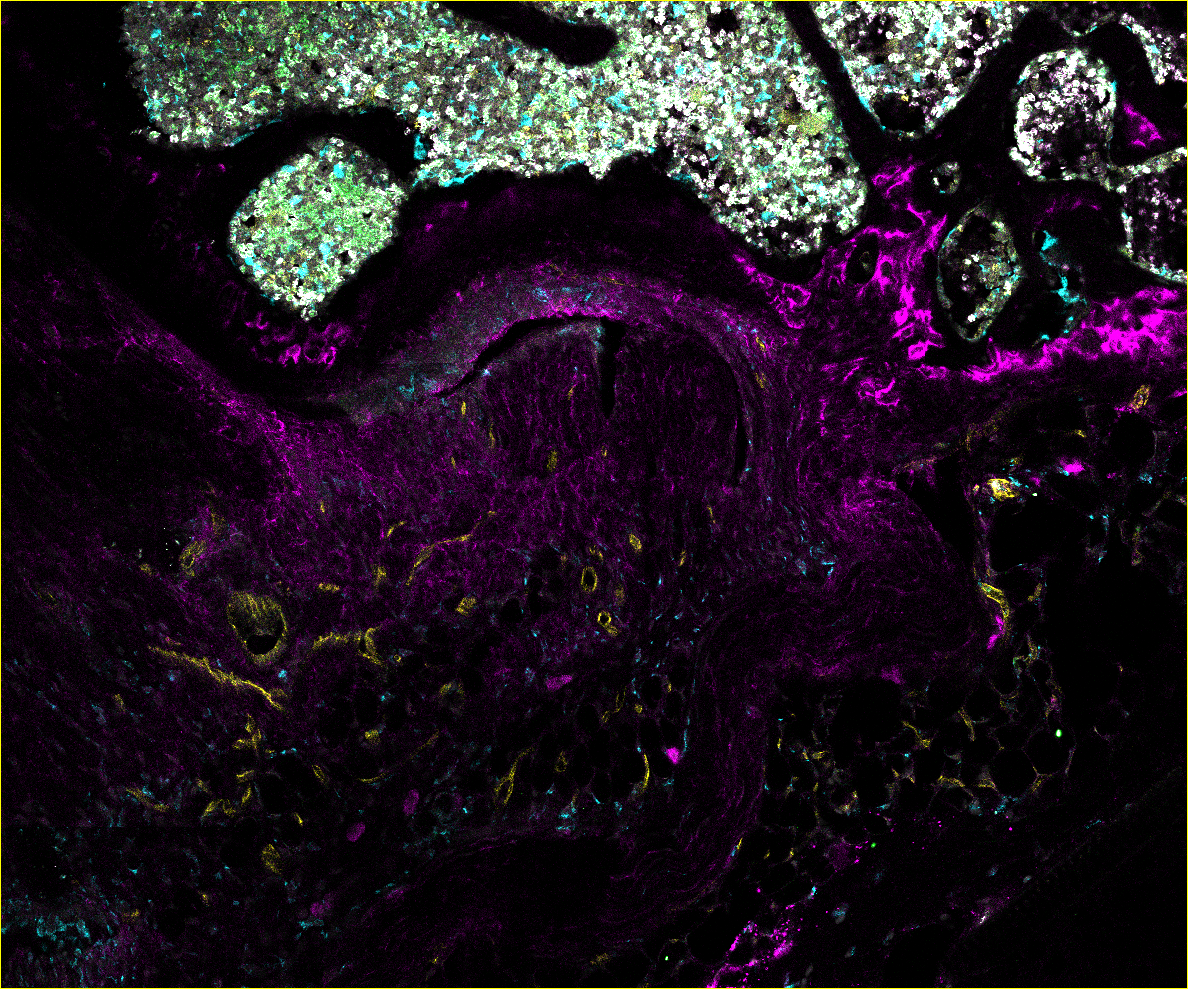

Supplement: Supplementary file 12 — Source data Fig. 5 [file 44320_2025_149_MOESM12_ESM.zip › Figure 5/5C/Day14_Full.tif]

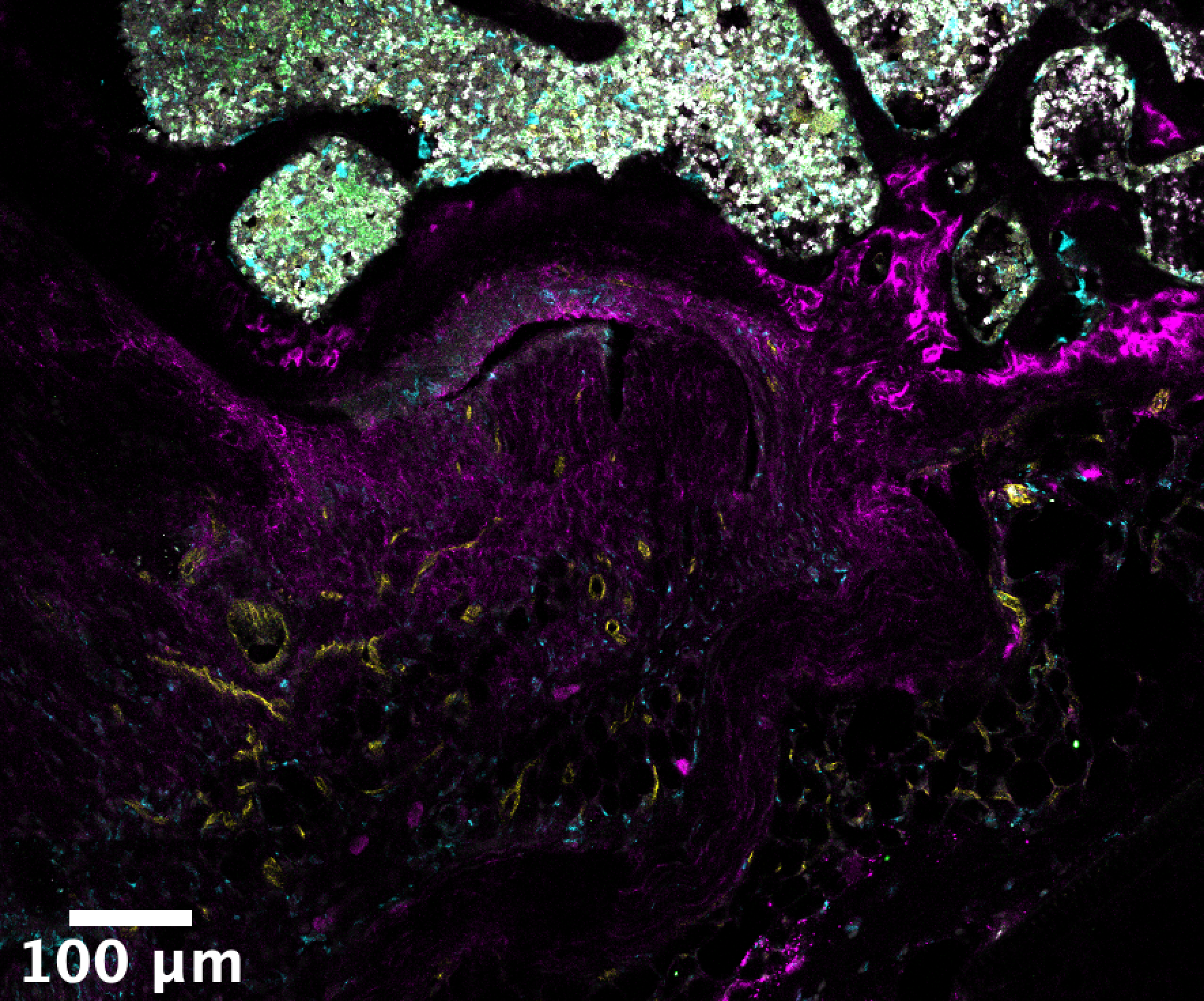

Supplement: Supplementary file 12 — Source data Fig. 5 [file 44320_2025_149_MOESM12_ESM.zip › Figure 5/5C/Day14_Full.png]

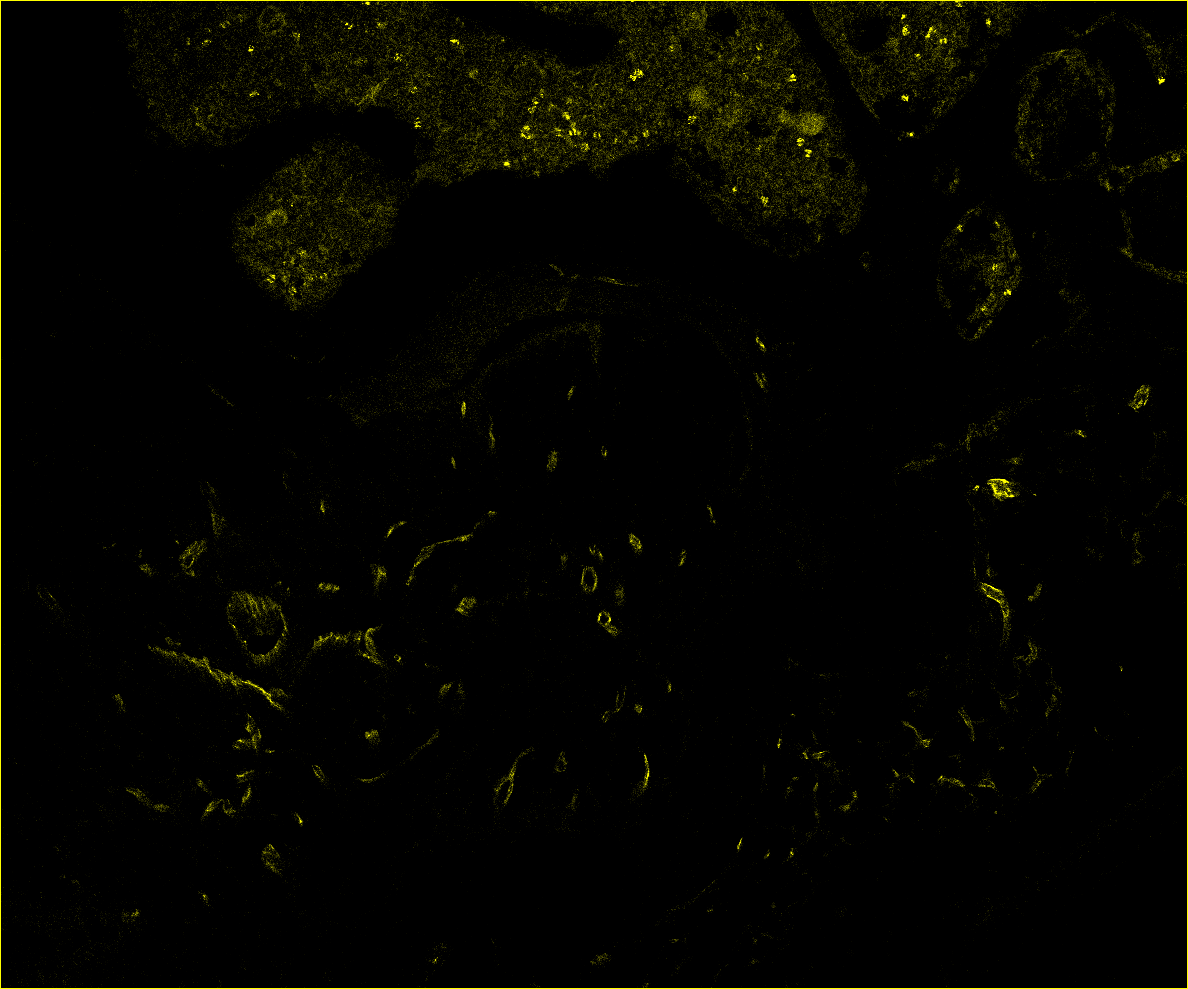

Supplement: Supplementary file 12 — Source data Fig. 5 [file 44320_2025_149_MOESM12_ESM.zip › Figure 5/5C/Day14_CD31.tif]

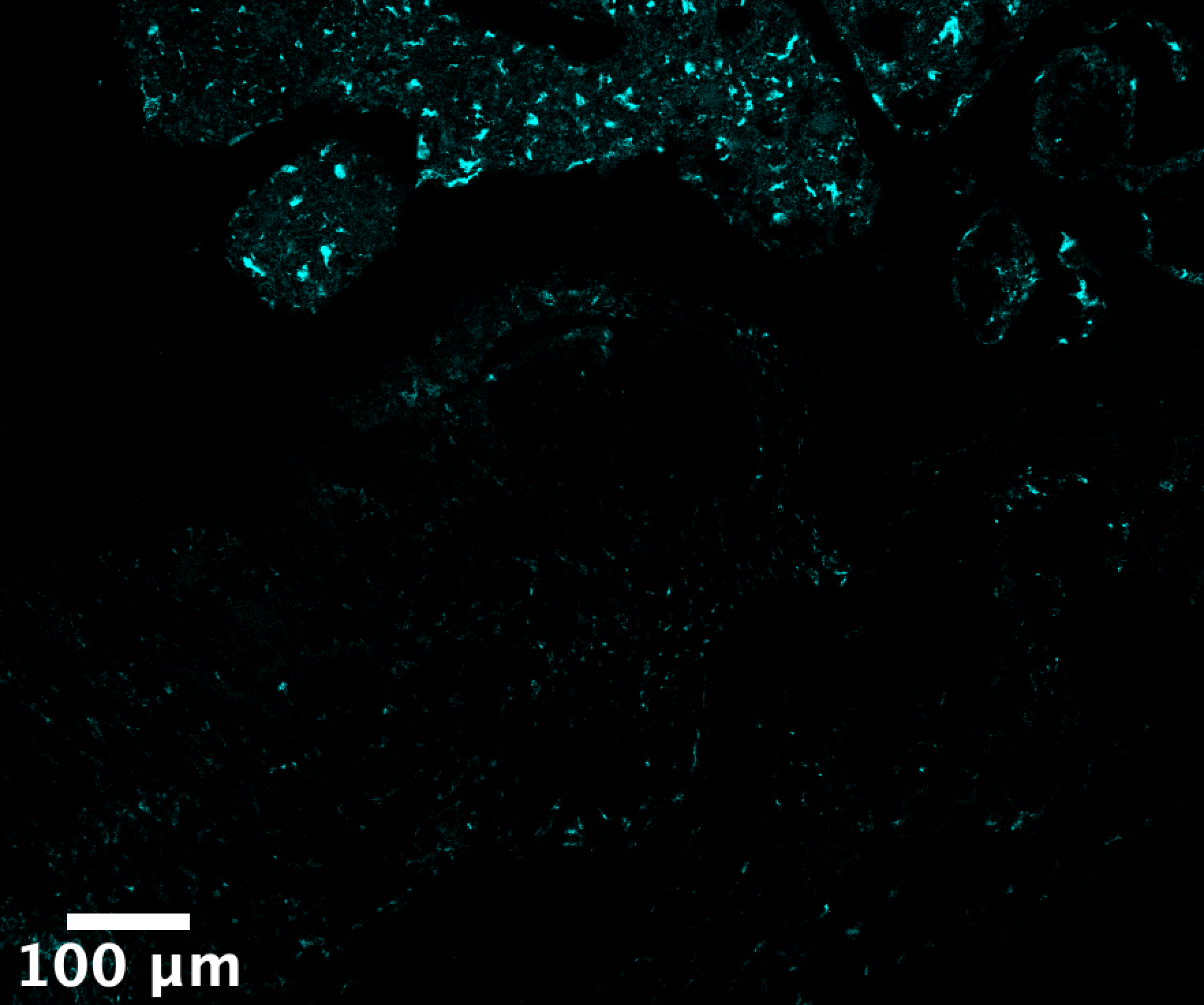

Supplement: Supplementary file 12 — Source data Fig. 5 [file 44320_2025_149_MOESM12_ESM.zip › Figure 5/5C/Day14_CD68.png]

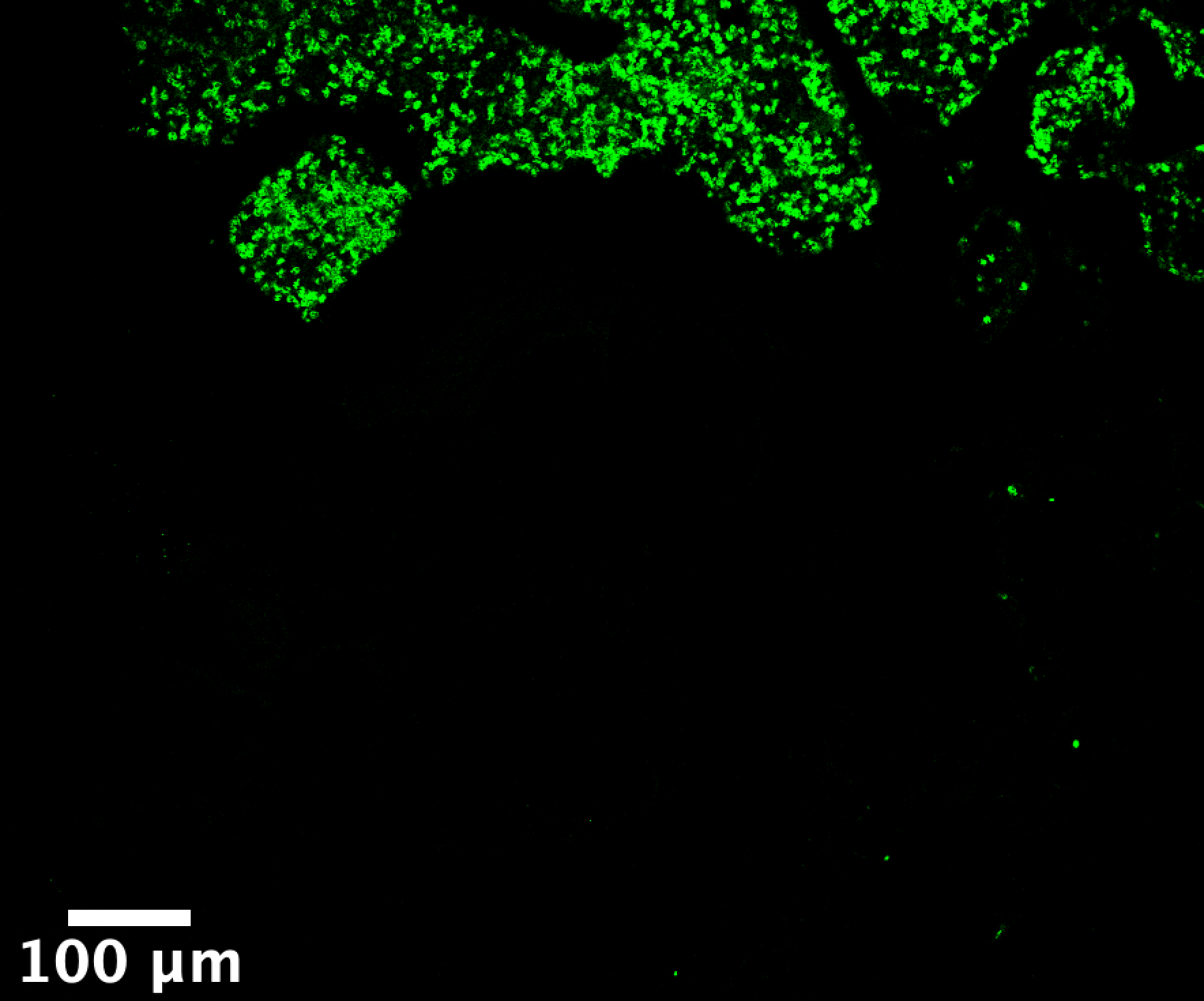

Supplement: Supplementary file 12 — Source data Fig. 5 [file 44320_2025_149_MOESM12_ESM.zip › Figure 5/5C/Day14_CD177.png]

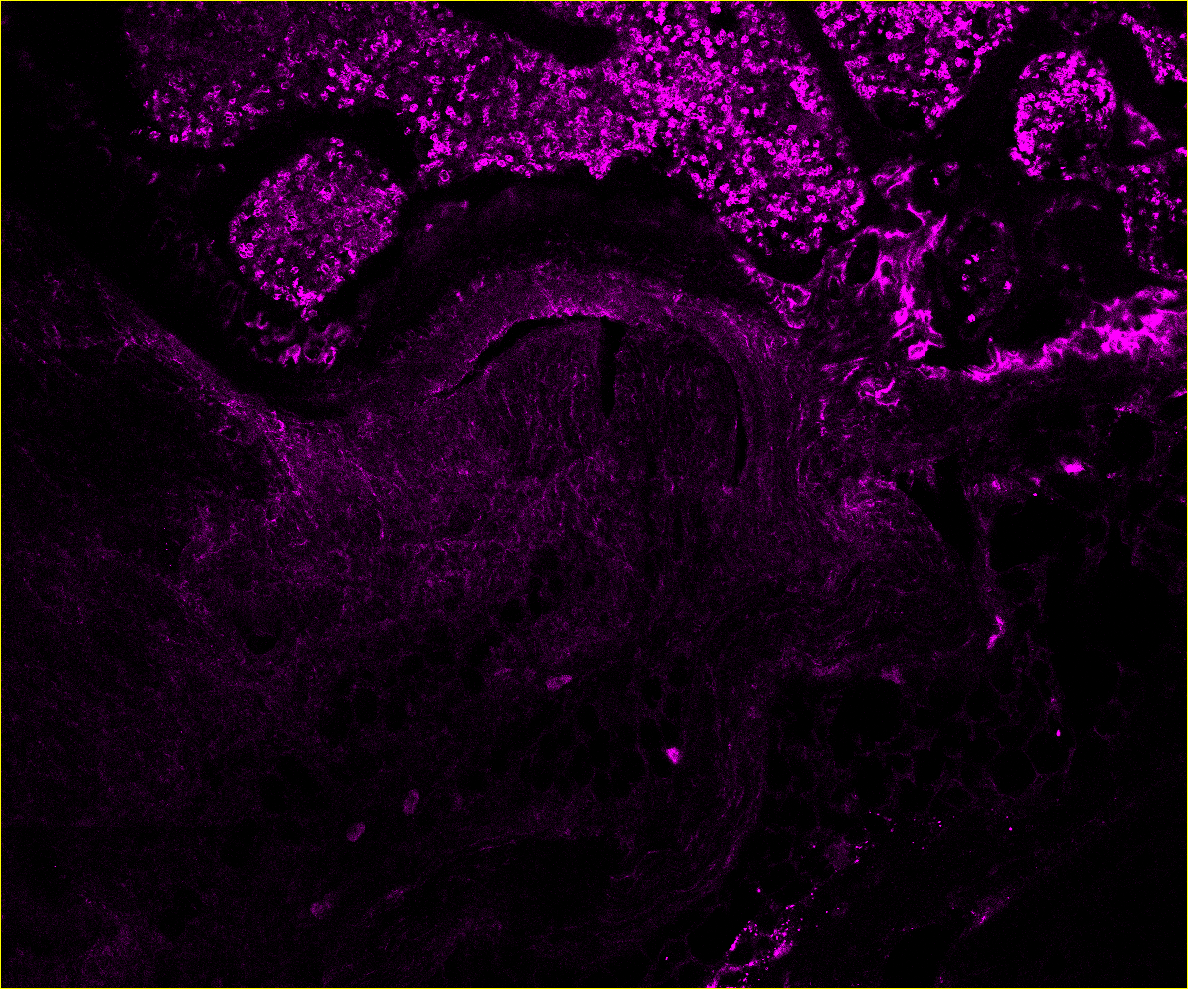

Supplement: Supplementary file 12 — Source data Fig. 5 [file 44320_2025_149_MOESM12_ESM.zip › Figure 5/5C/Day14_COL6.tif]

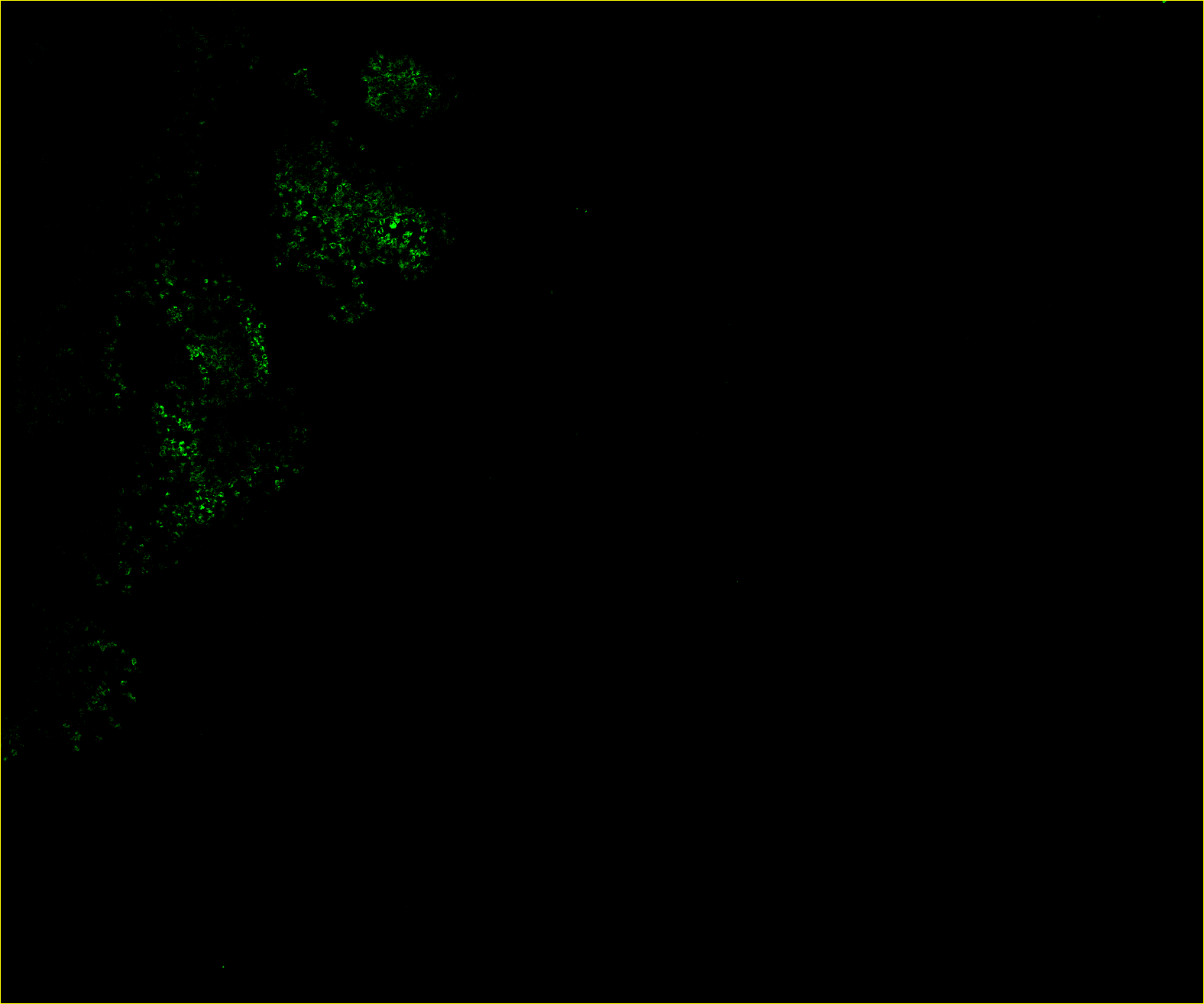

Supplement: Supplementary file 12 — Source data Fig. 5 [file 44320_2025_149_MOESM12_ESM.zip › Figure 5/5C/Naive_CD177.tif]

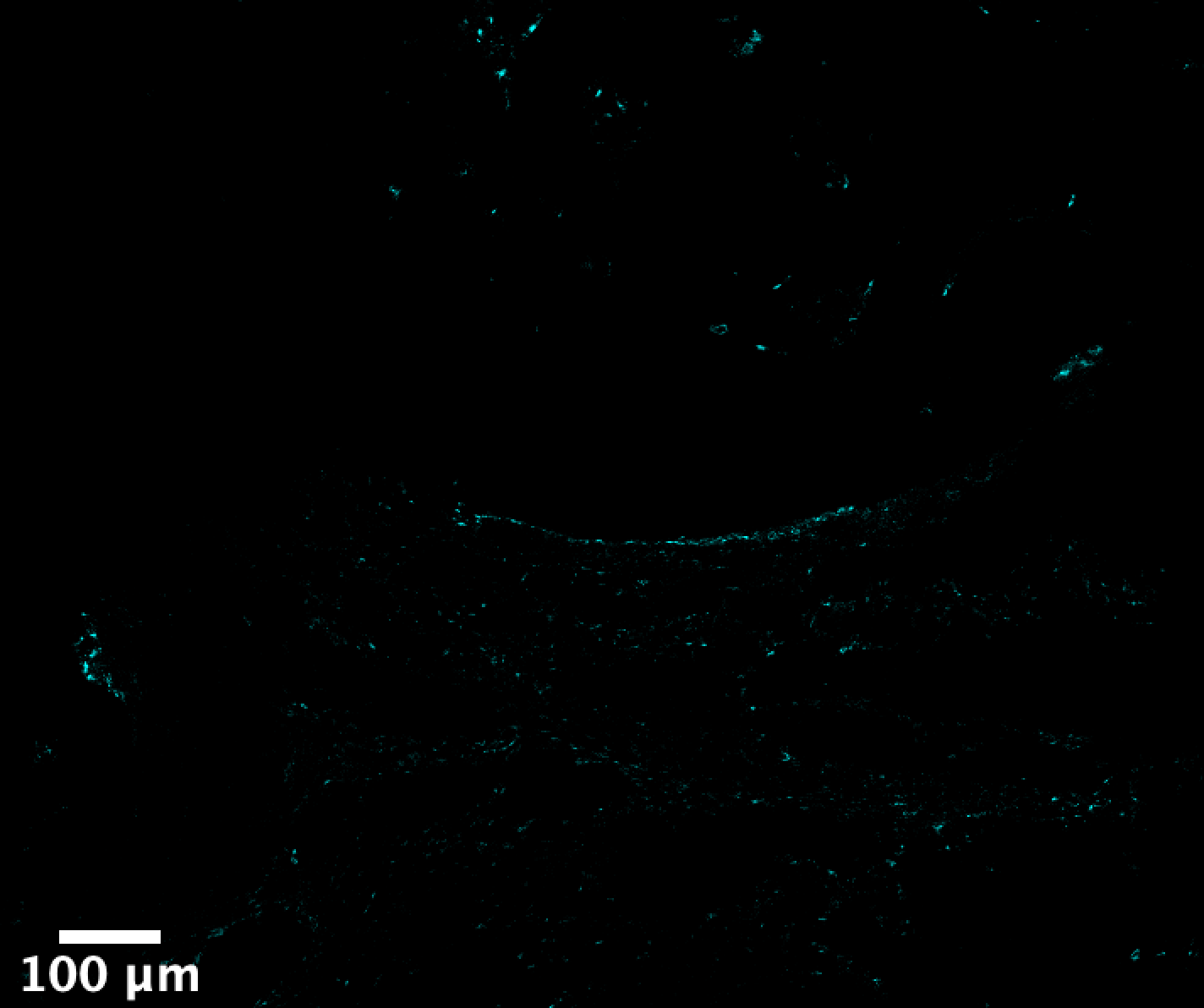

Supplement: Supplementary file 12 — Source data Fig. 5 [file 44320_2025_149_MOESM12_ESM.zip › Figure 5/5C/Day2_CD68.png]

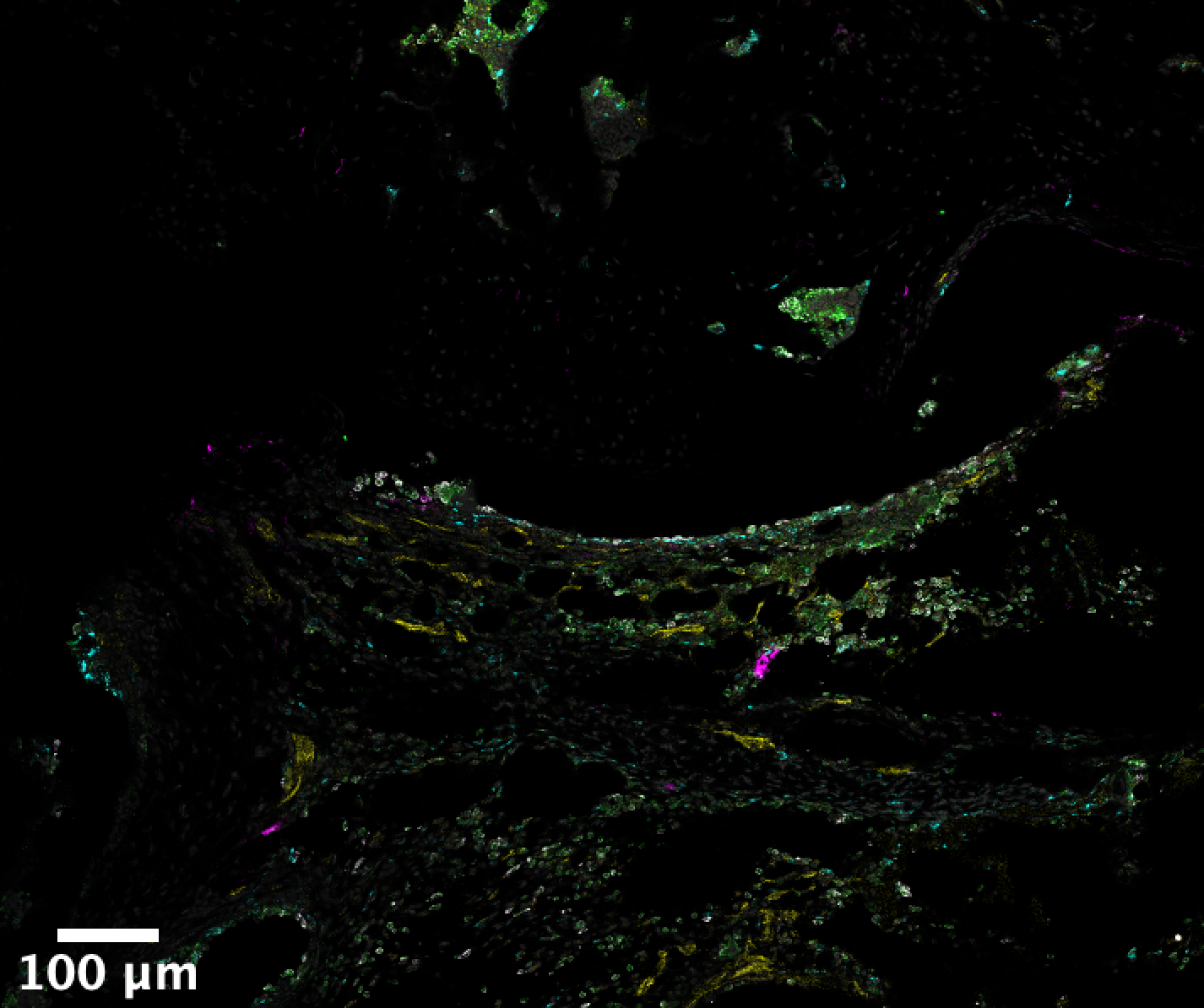

Supplement: Supplementary file 12 — Source data Fig. 5 [file 44320_2025_149_MOESM12_ESM.zip › Figure 5/5C/Day2_Full.png]

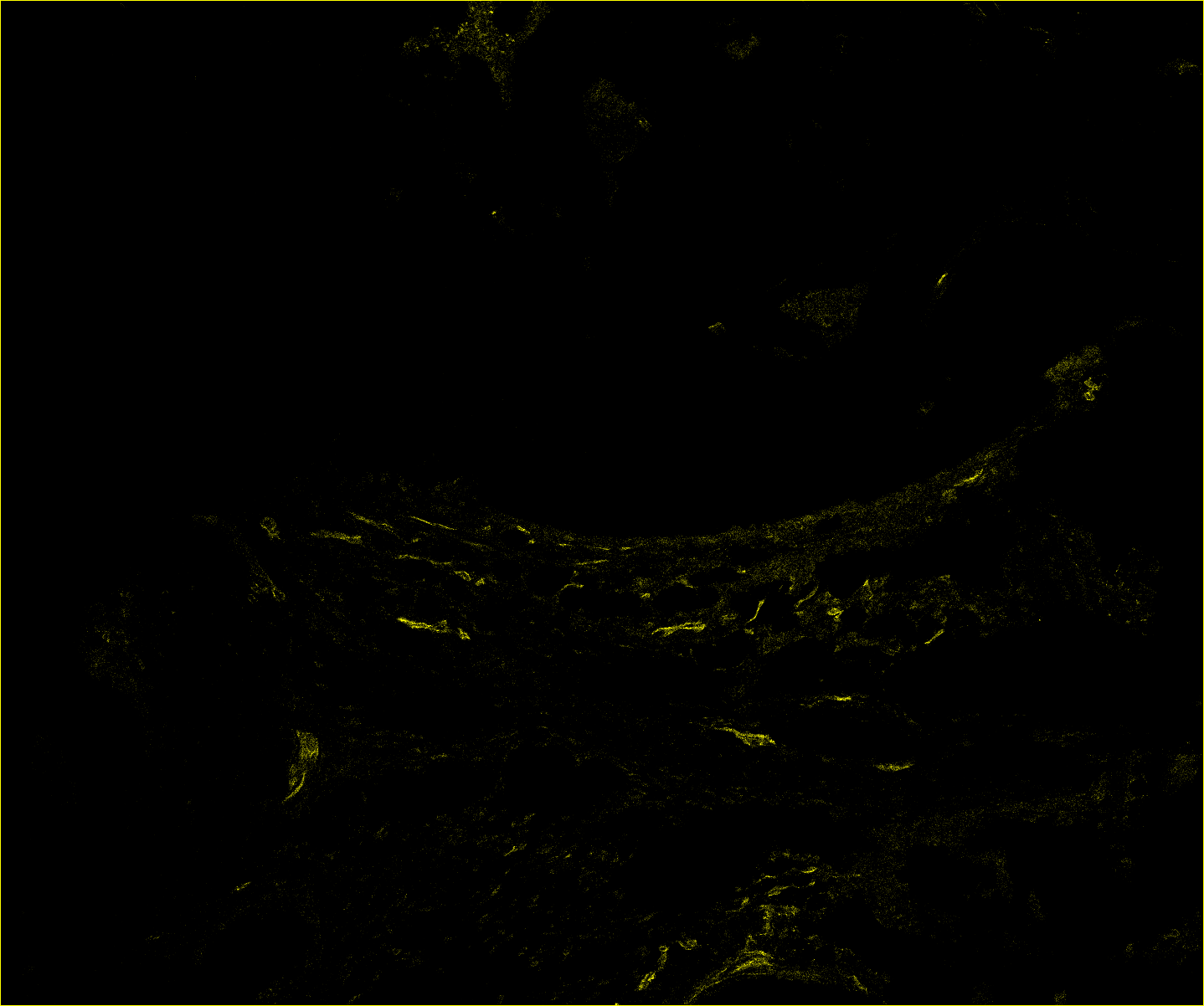

Supplement: Supplementary file 12 — Source data Fig. 5 [file 44320_2025_149_MOESM12_ESM.zip › Figure 5/5C/Day2_CD31.tif]

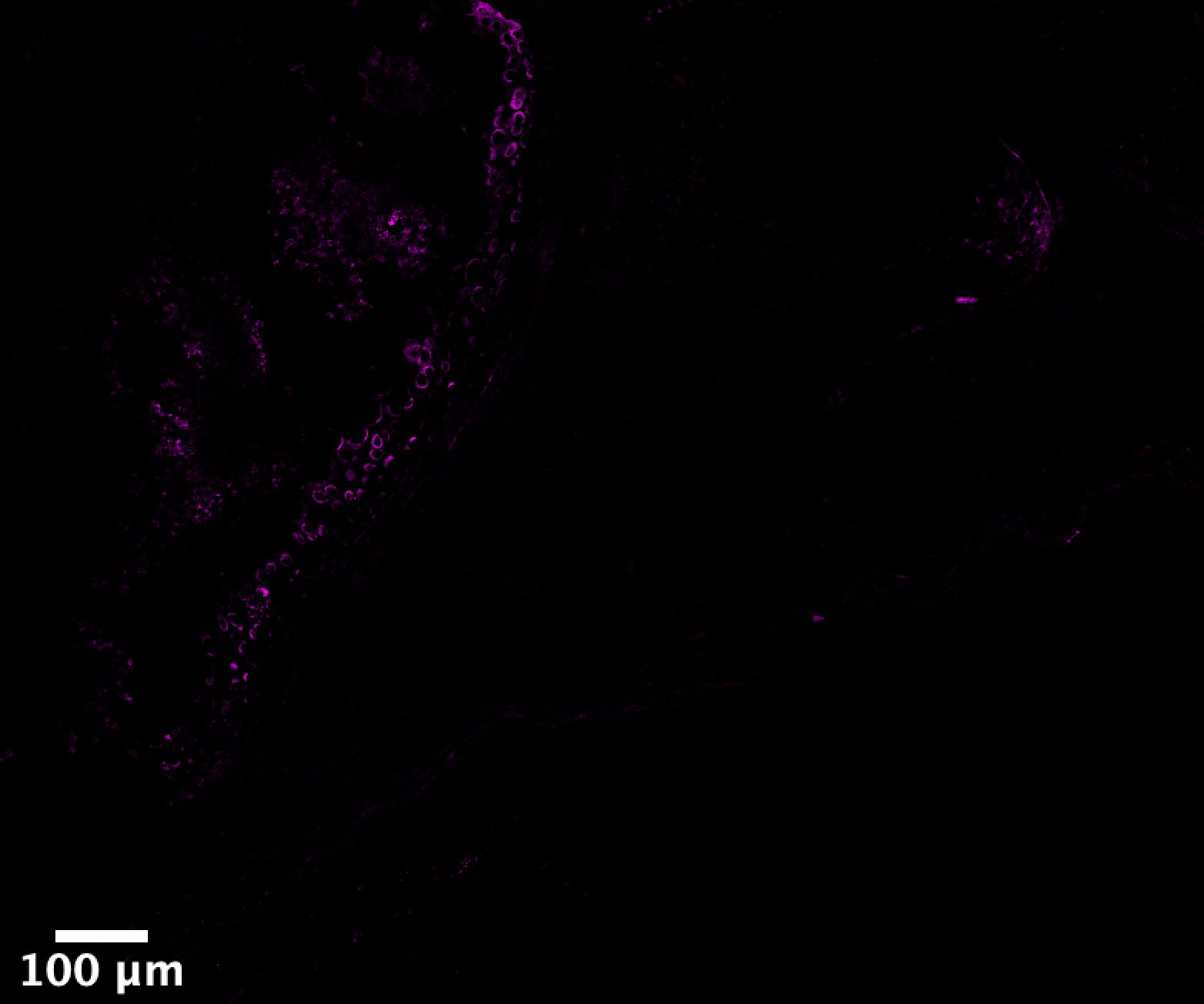

Supplement: Supplementary file 12 — Source data Fig. 5 [file 44320_2025_149_MOESM12_ESM.zip › Figure 5/5C/Naive_COL6.png]

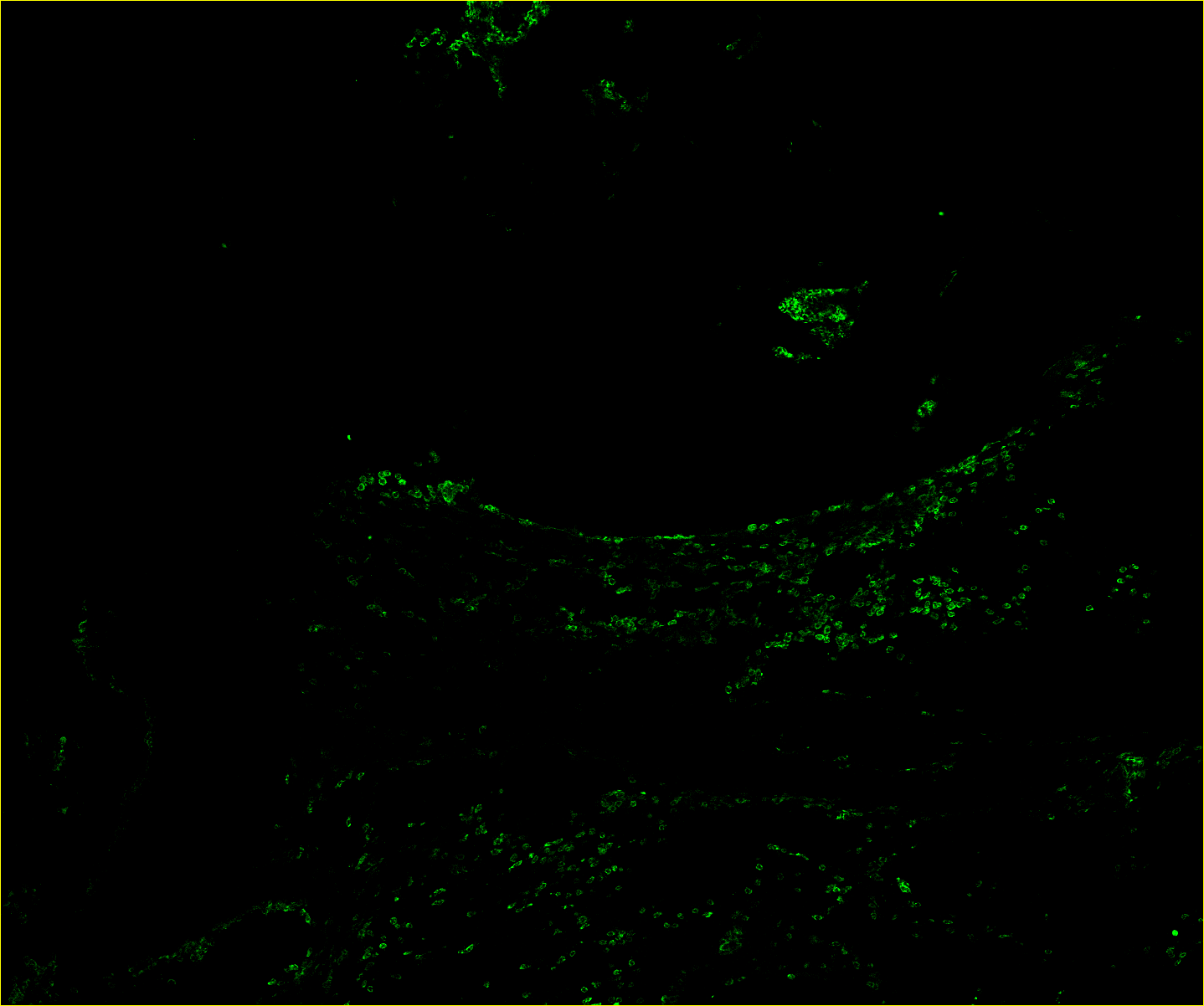

Supplement: Supplementary file 12 — Source data Fig. 5 [file 44320_2025_149_MOESM12_ESM.zip › Figure 5/5C/Day2_CD177.tif]

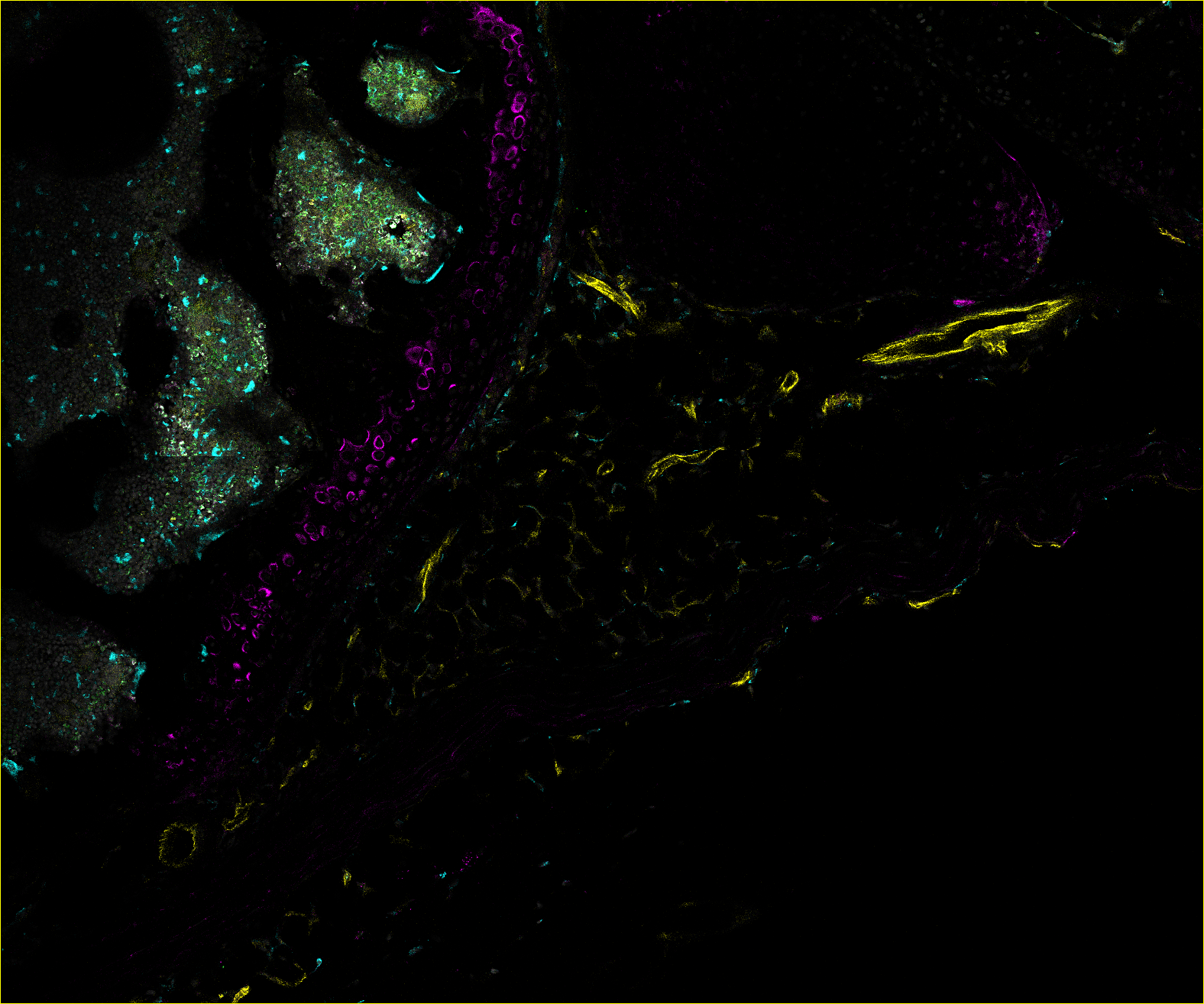

Supplement: Supplementary file 12 — Source data Fig. 5 [file 44320_2025_149_MOESM12_ESM.zip › Figure 5/5C/Naive_Full.tif]

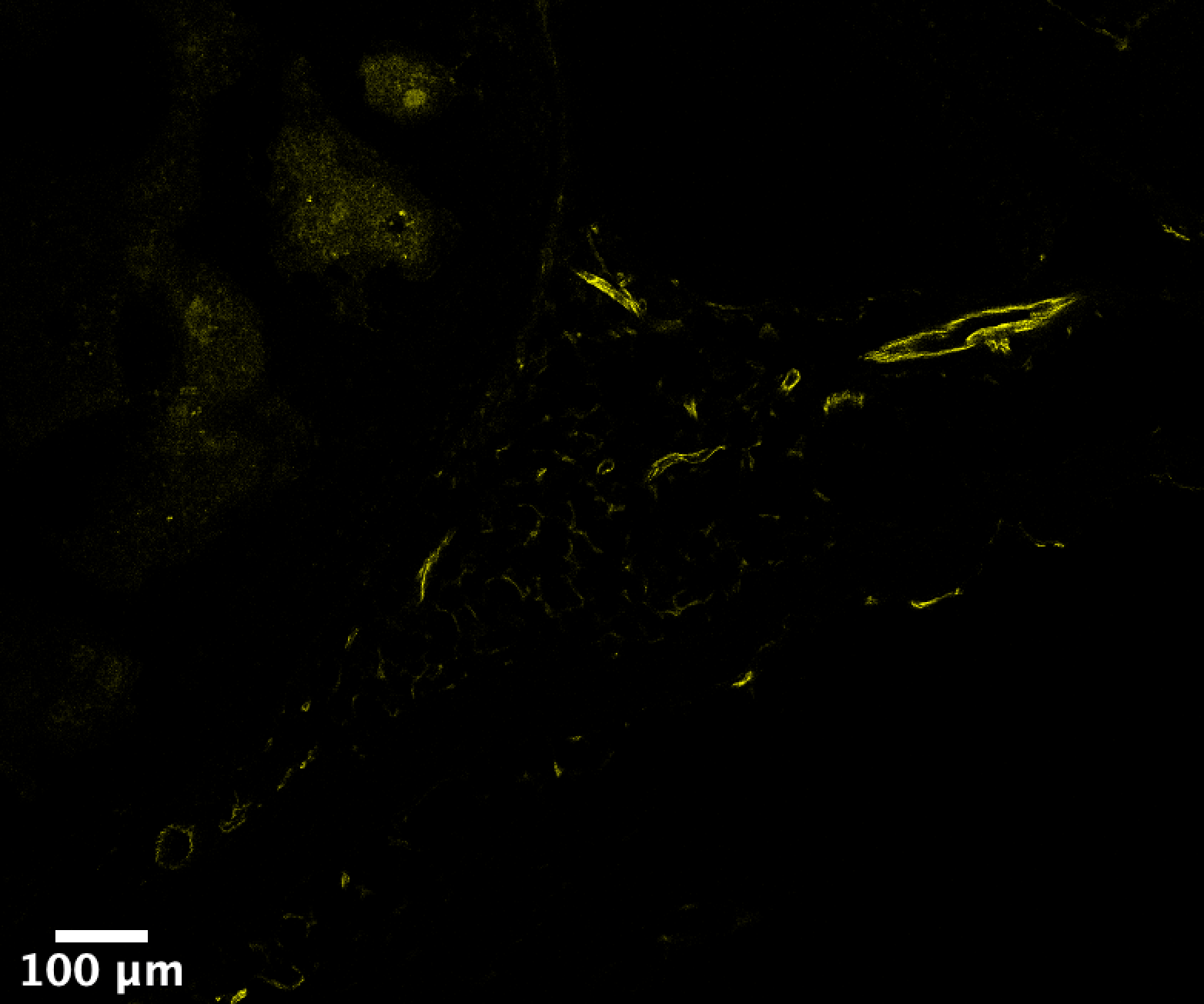

Supplement: Supplementary file 12 — Source data Fig. 5 [file 44320_2025_149_MOESM12_ESM.zip › Figure 5/5C/Naive_CD31.png]

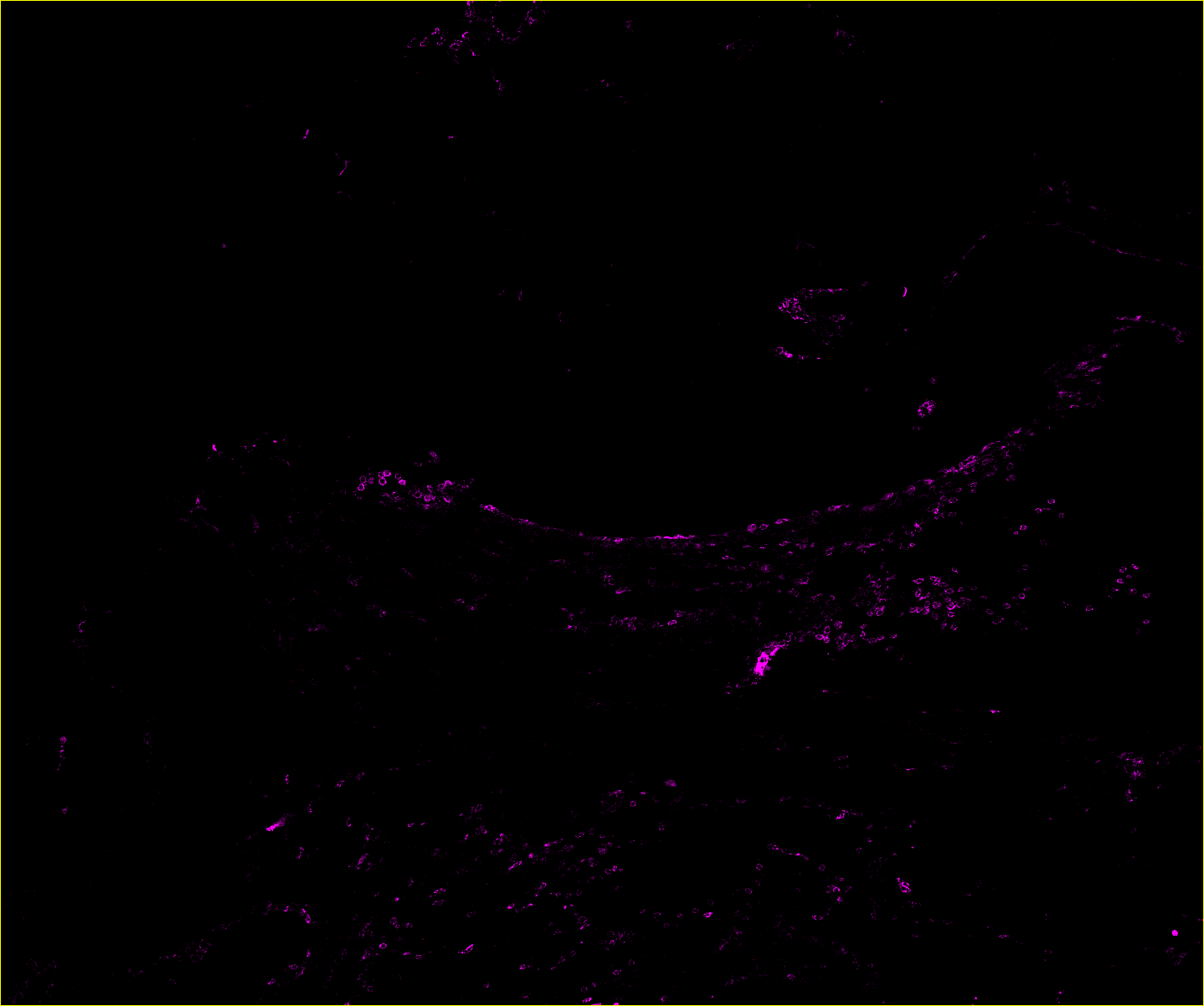

Supplement: Supplementary file 12 — Source data Fig. 5 [file 44320_2025_149_MOESM12_ESM.zip › Figure 5/5C/Day2_COL6.tif]

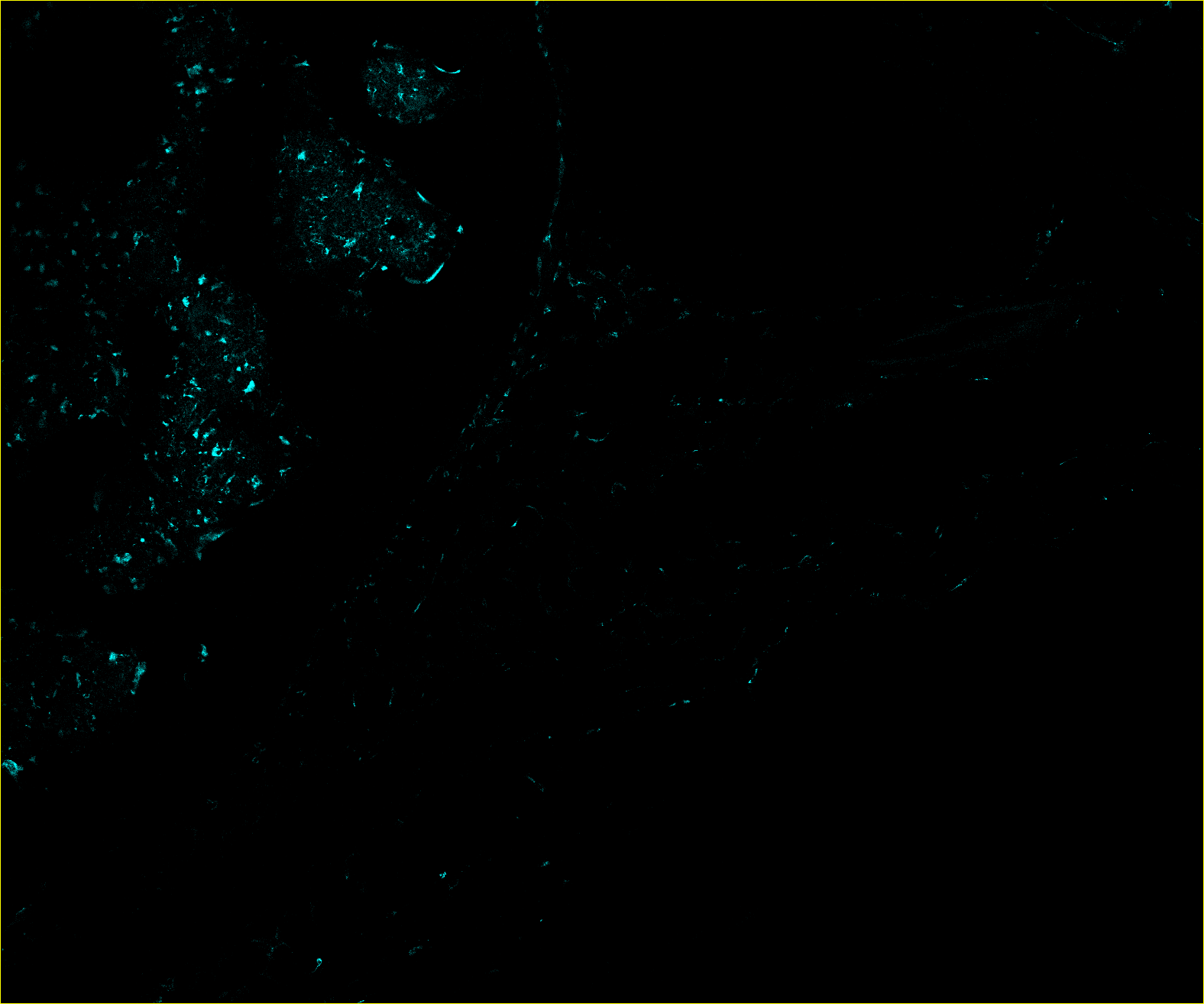

Supplement: Supplementary file 12 — Source data Fig. 5 [file 44320_2025_149_MOESM12_ESM.zip › Figure 5/5C/Naive_CD68.tif]

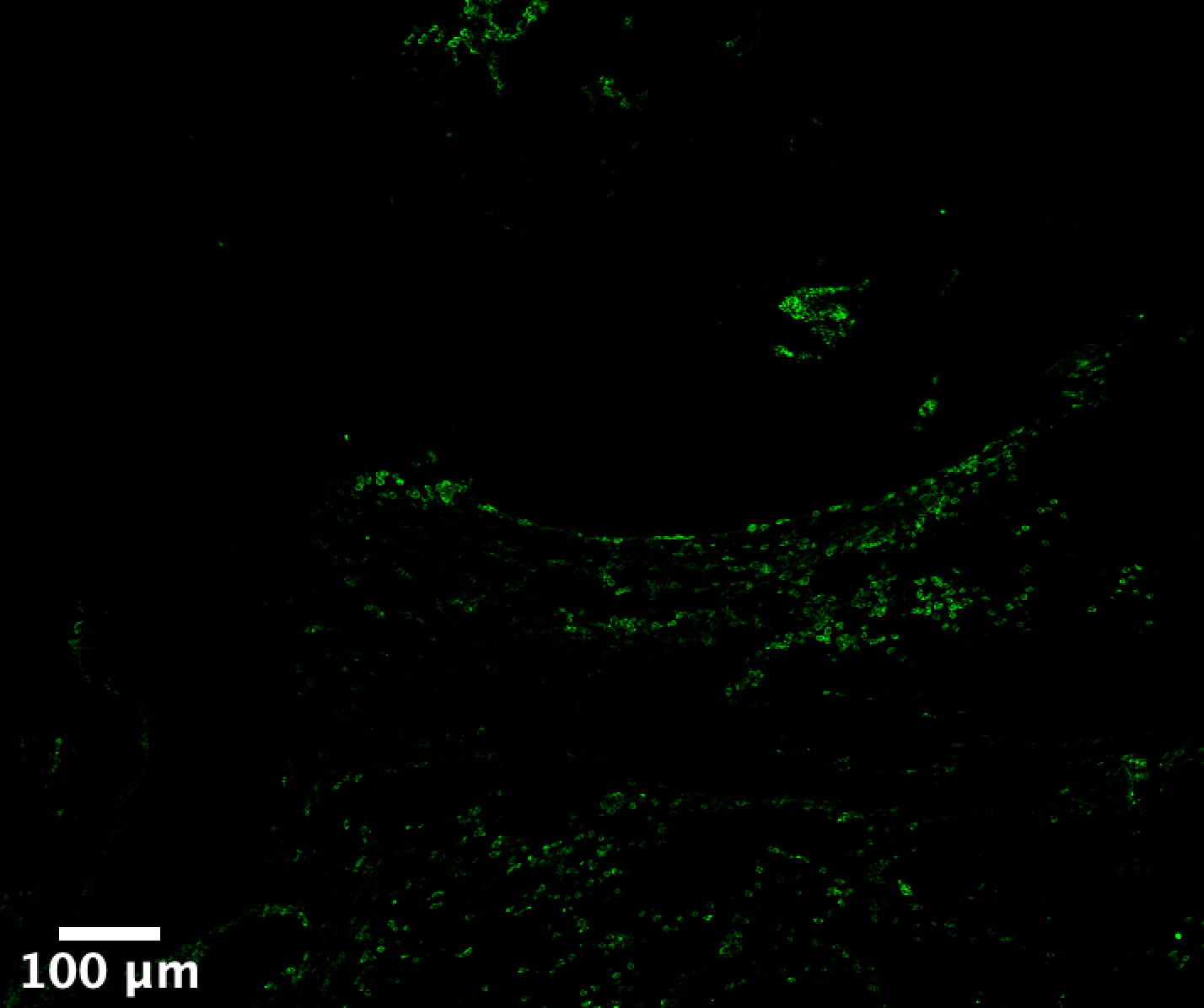

Supplement: Supplementary file 12 — Source data Fig. 5 [file 44320_2025_149_MOESM12_ESM.zip › Figure 5/5C/Day2_CD177.png]

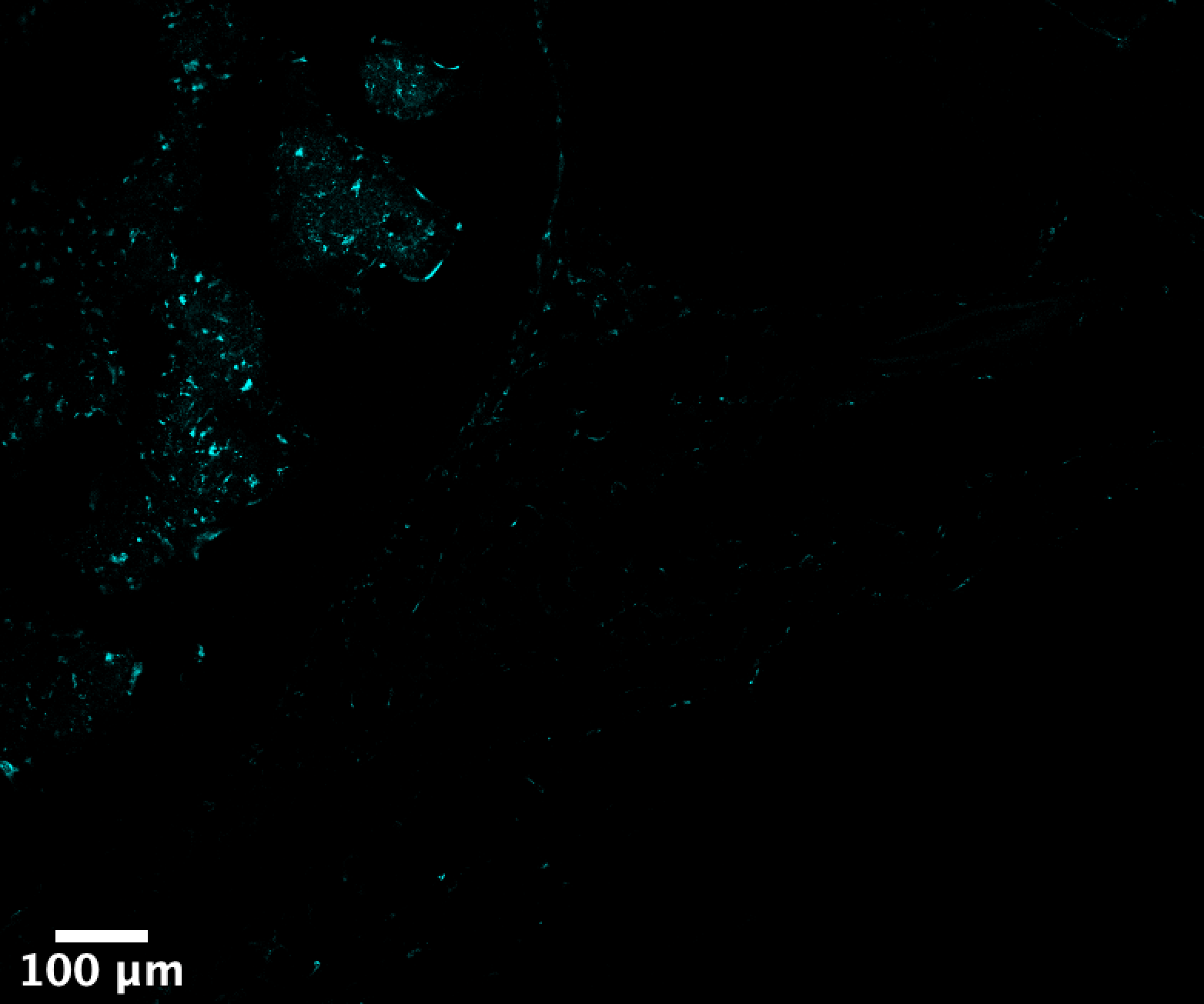

Supplement: Supplementary file 12 — Source data Fig. 5 [file 44320_2025_149_MOESM12_ESM.zip › Figure 5/5C/Naive_CD68.png]

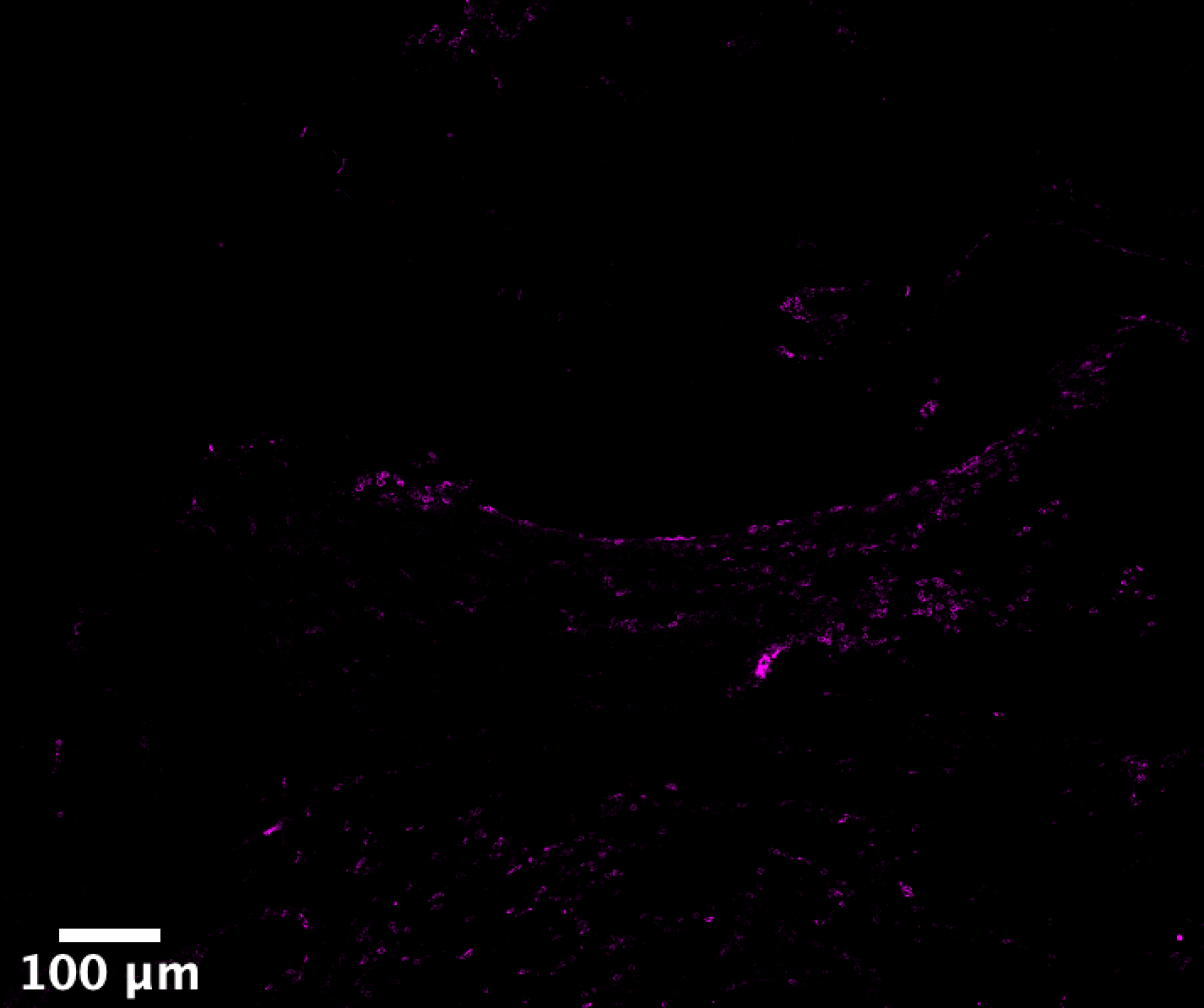

Supplement: Supplementary file 12 — Source data Fig. 5 [file 44320_2025_149_MOESM12_ESM.zip › Figure 5/5C/Day2_COL6.png]

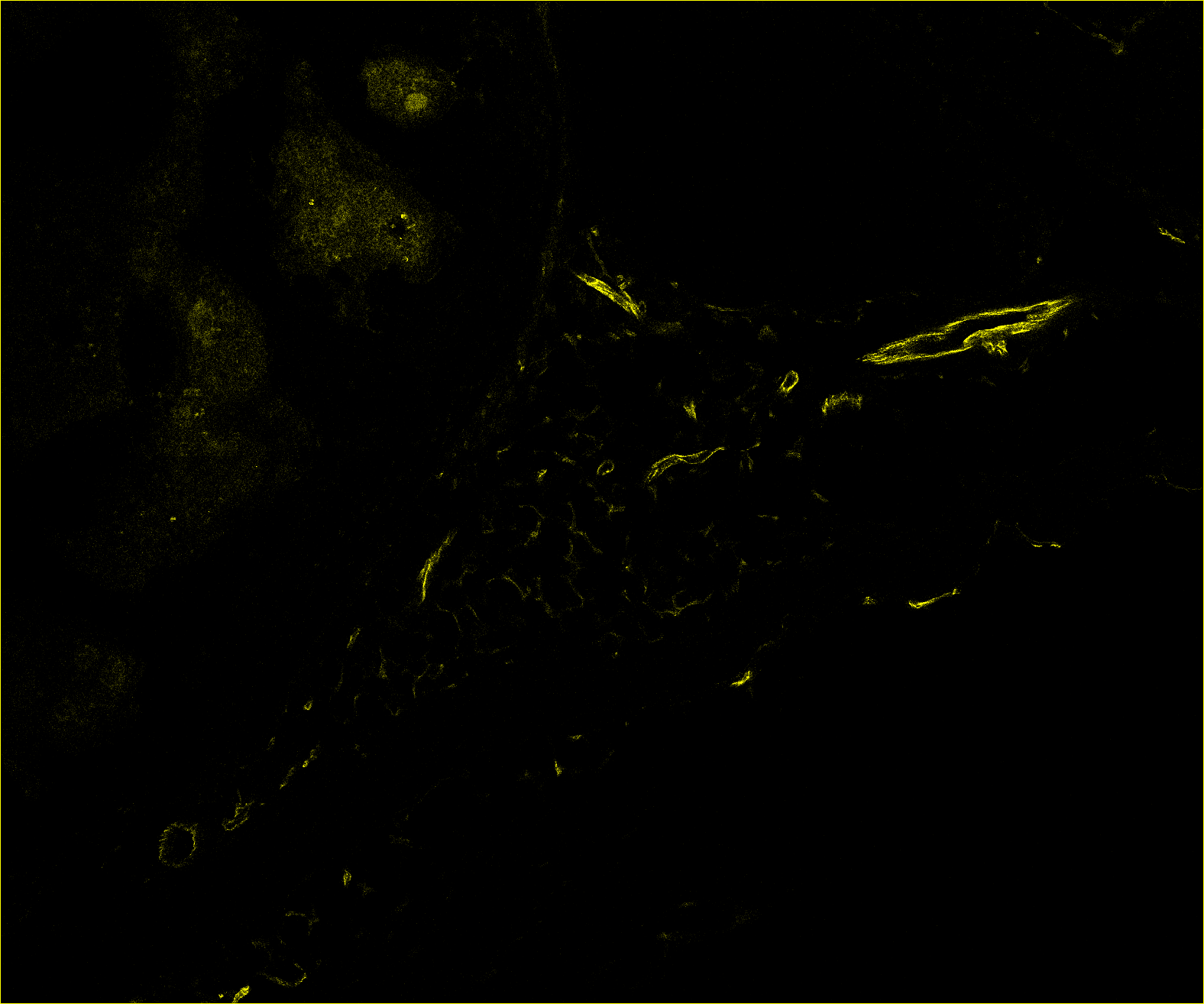

Supplement: Supplementary file 12 — Source data Fig. 5 [file 44320_2025_149_MOESM12_ESM.zip › Figure 5/5C/Naive_CD31.tif]

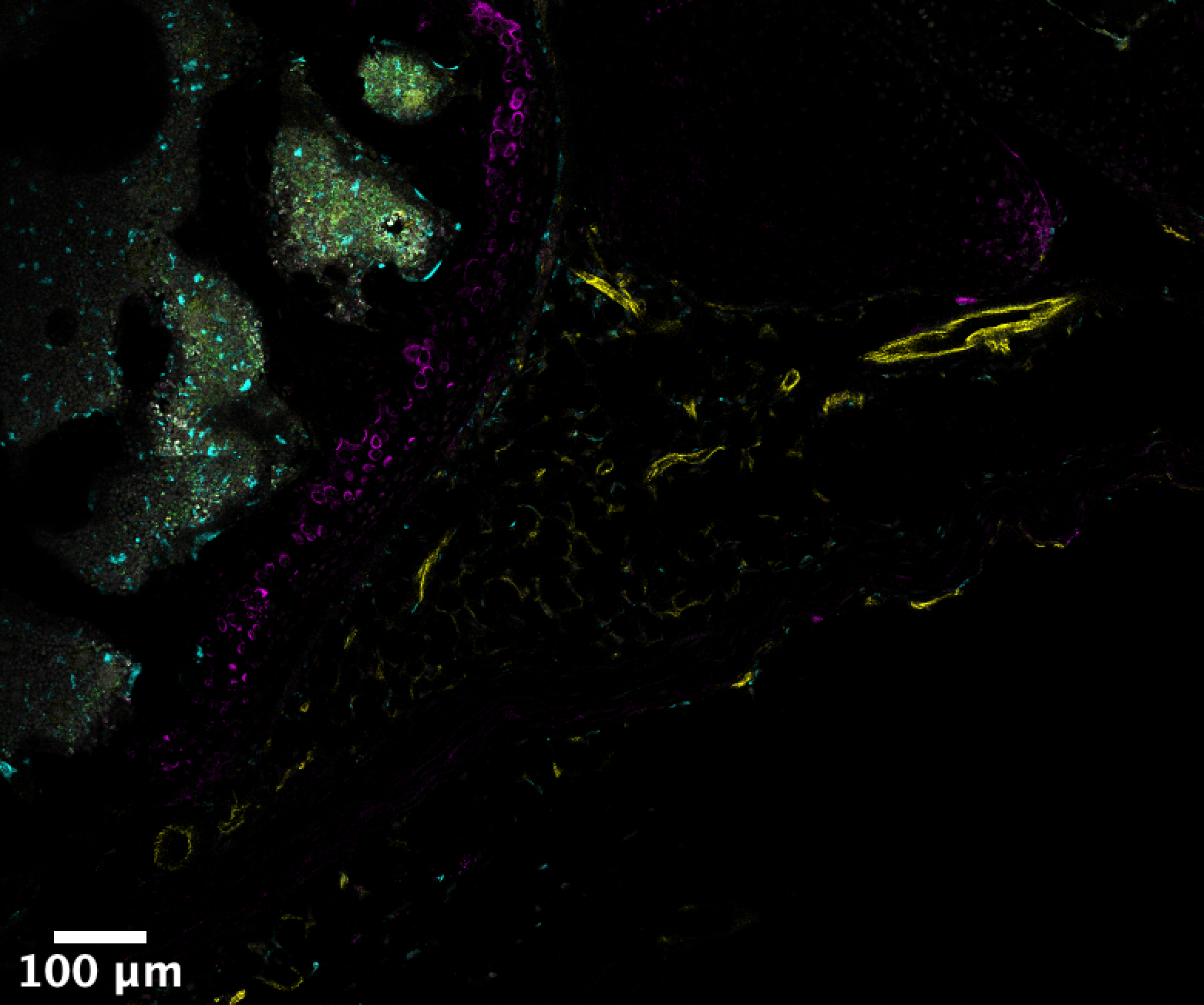

Supplement: Supplementary file 12 — Source data Fig. 5 [file 44320_2025_149_MOESM12_ESM.zip › Figure 5/5C/Naive_Full.png]

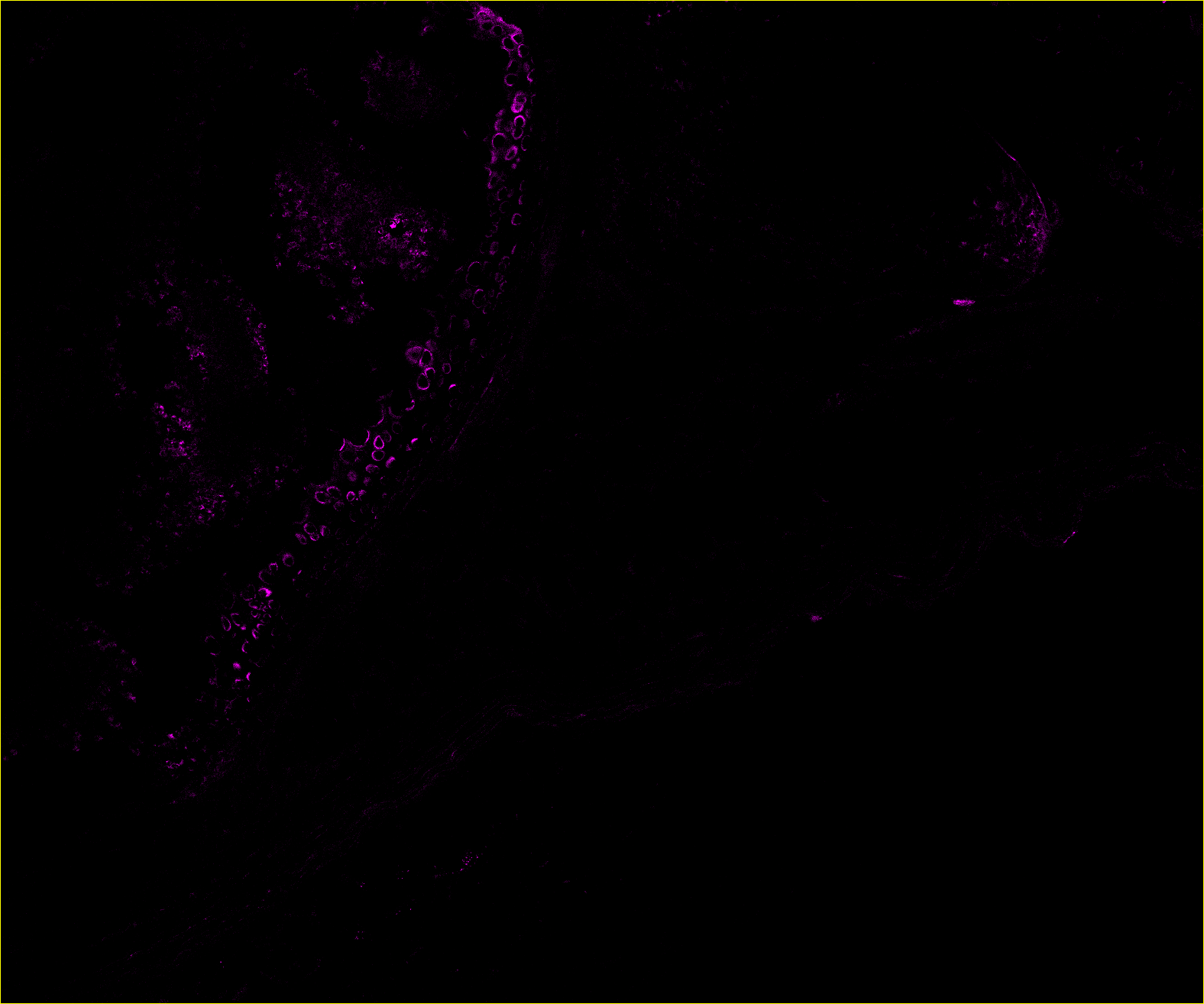

Supplement: Supplementary file 12 — Source data Fig. 5 [file 44320_2025_149_MOESM12_ESM.zip › Figure 5/5C/Naive_COL6.tif]

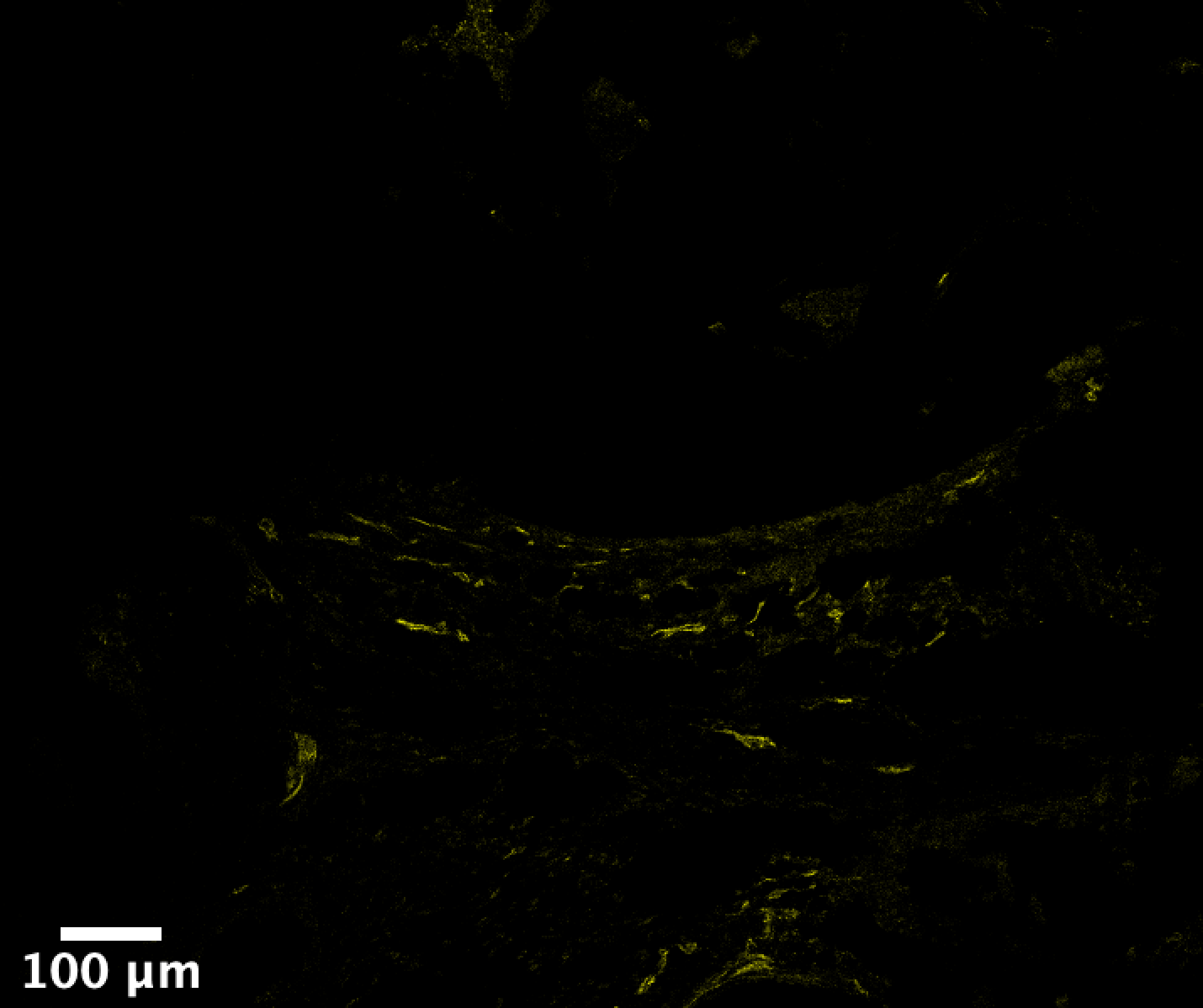

Supplement: Supplementary file 12 — Source data Fig. 5 [file 44320_2025_149_MOESM12_ESM.zip › Figure 5/5C/Day2_CD31.png]

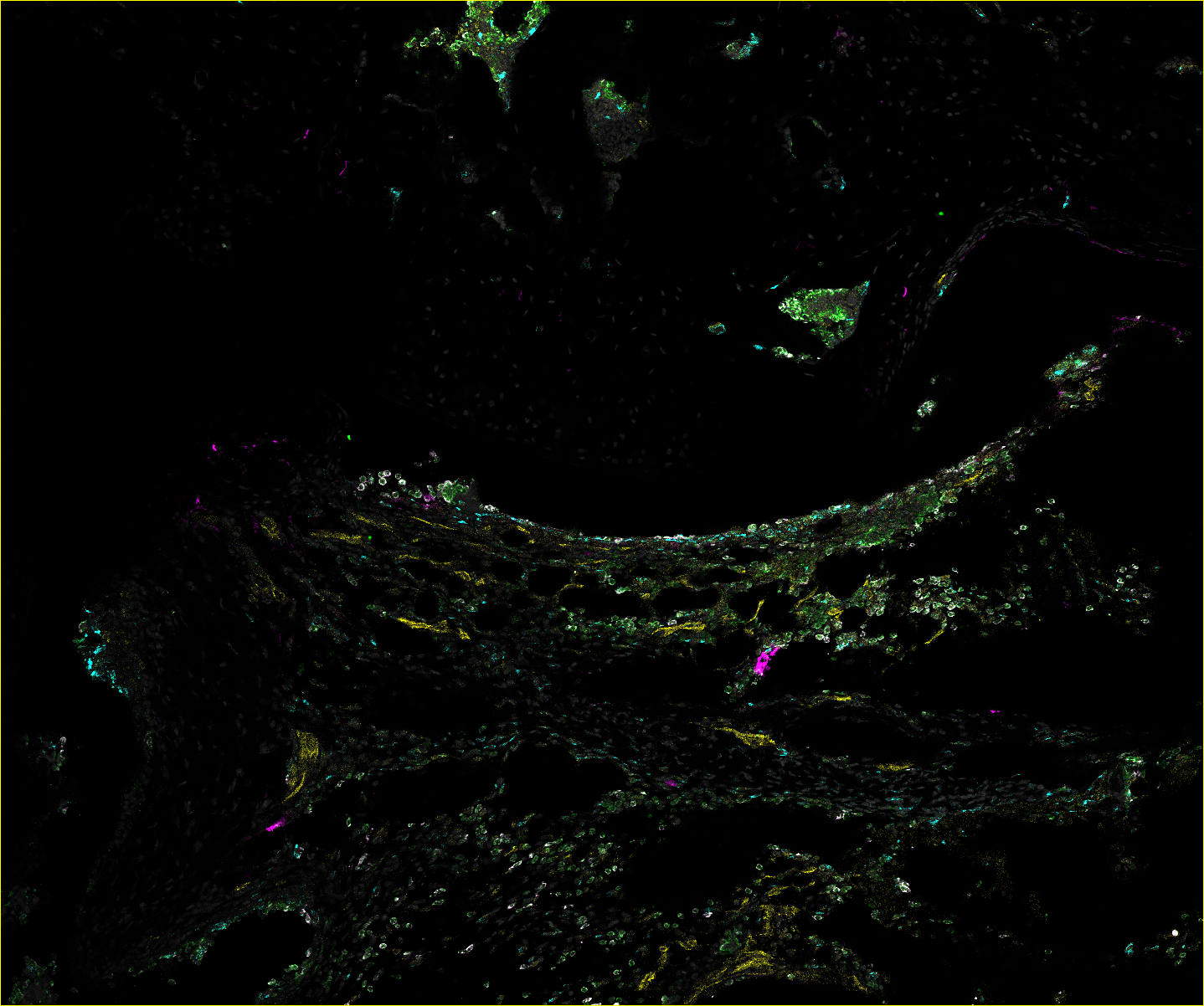

Supplement: Supplementary file 12 — Source data Fig. 5 [file 44320_2025_149_MOESM12_ESM.zip › Figure 5/5C/Day2_Full.tif]

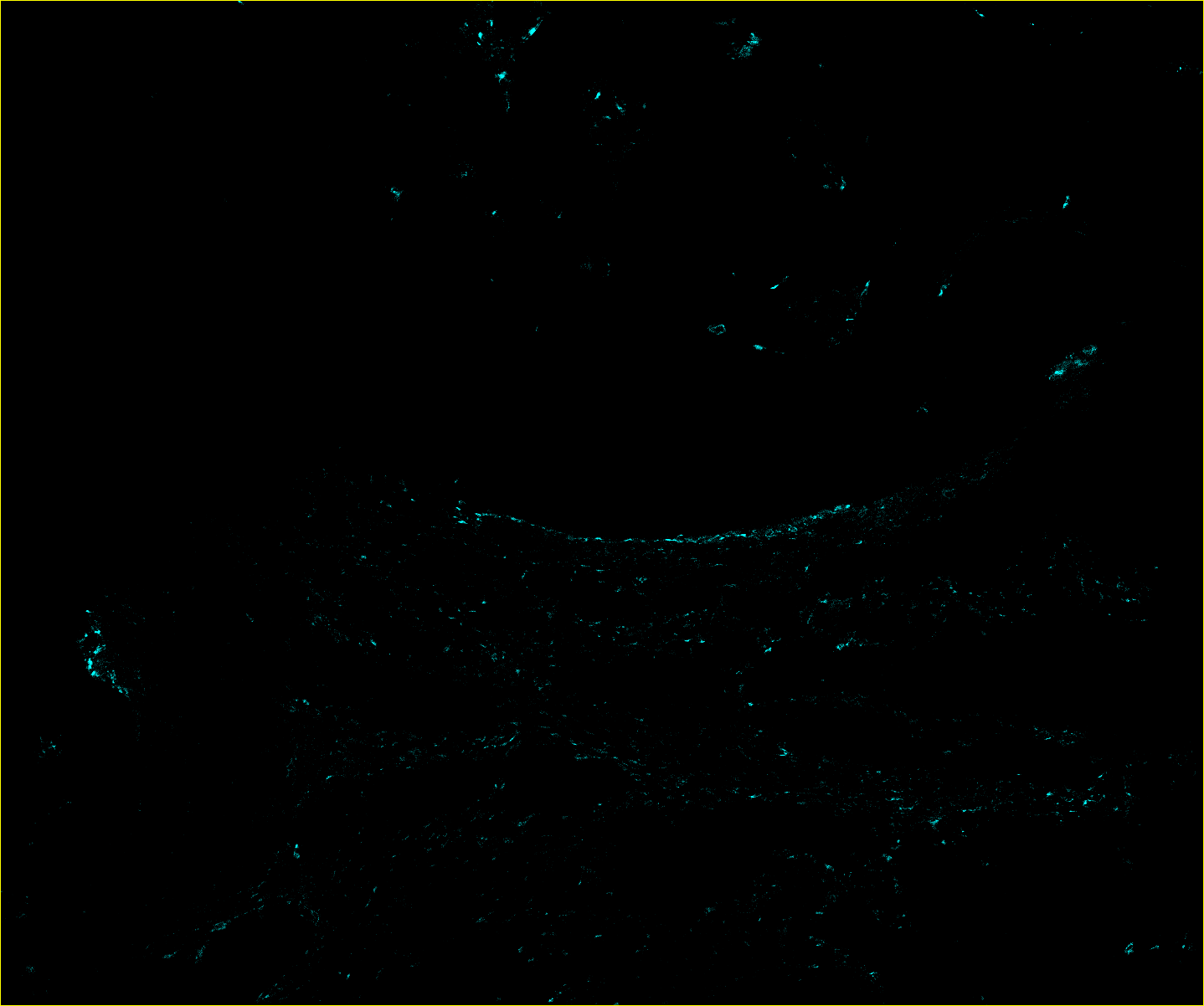

Supplement: Supplementary file 12 — Source data Fig. 5 [file 44320_2025_149_MOESM12_ESM.zip › Figure 5/5C/Day2_CD68.tif]

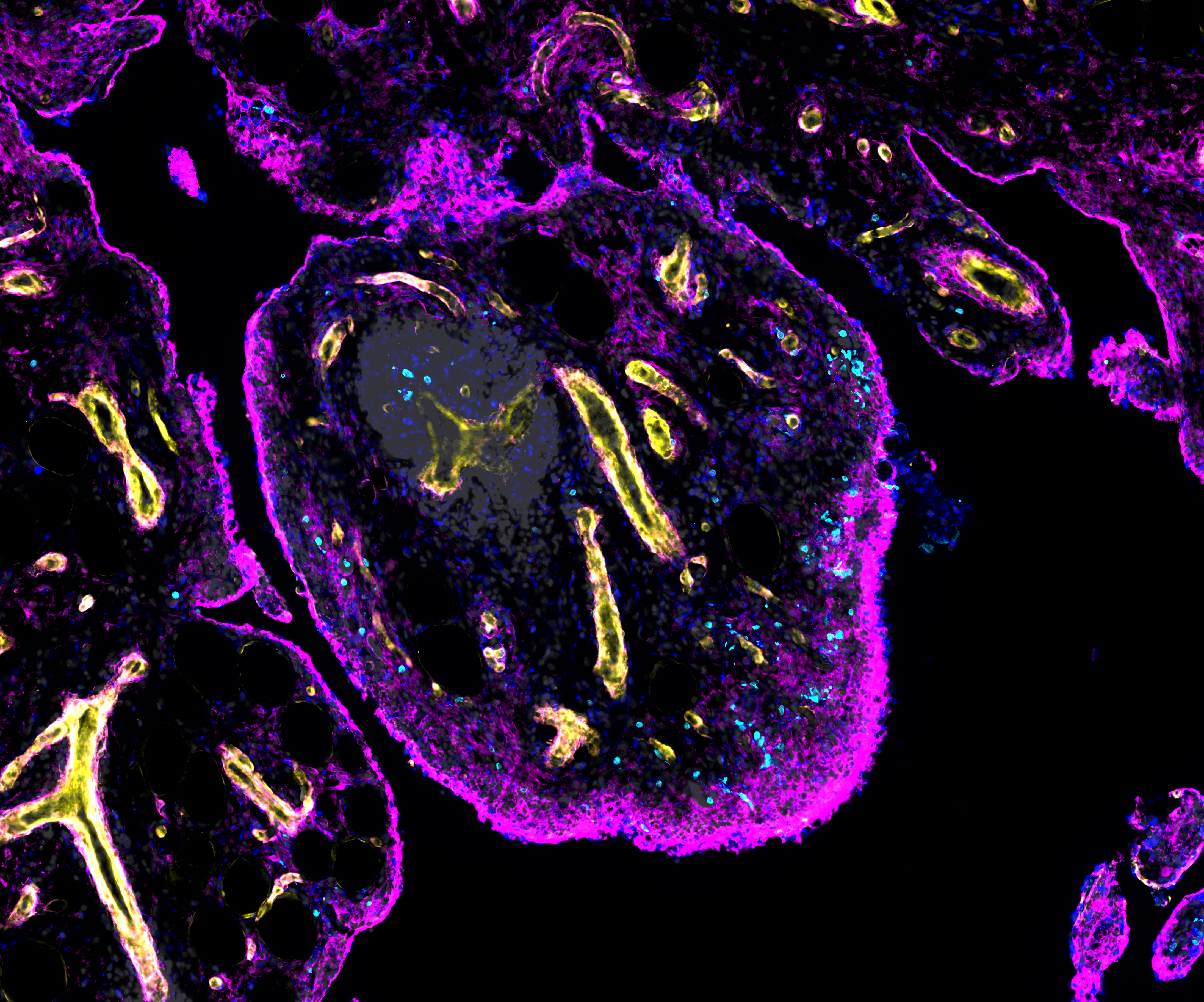

Supplement: Supplementary file 12 — Source data Fig. 5 [file 44320_2025_149_MOESM12_ESM.zip › Figure 5/5B/S00292623_LowOA_Fig5 (1, x=53566, y=13884, w=4381, h=3645).tif]

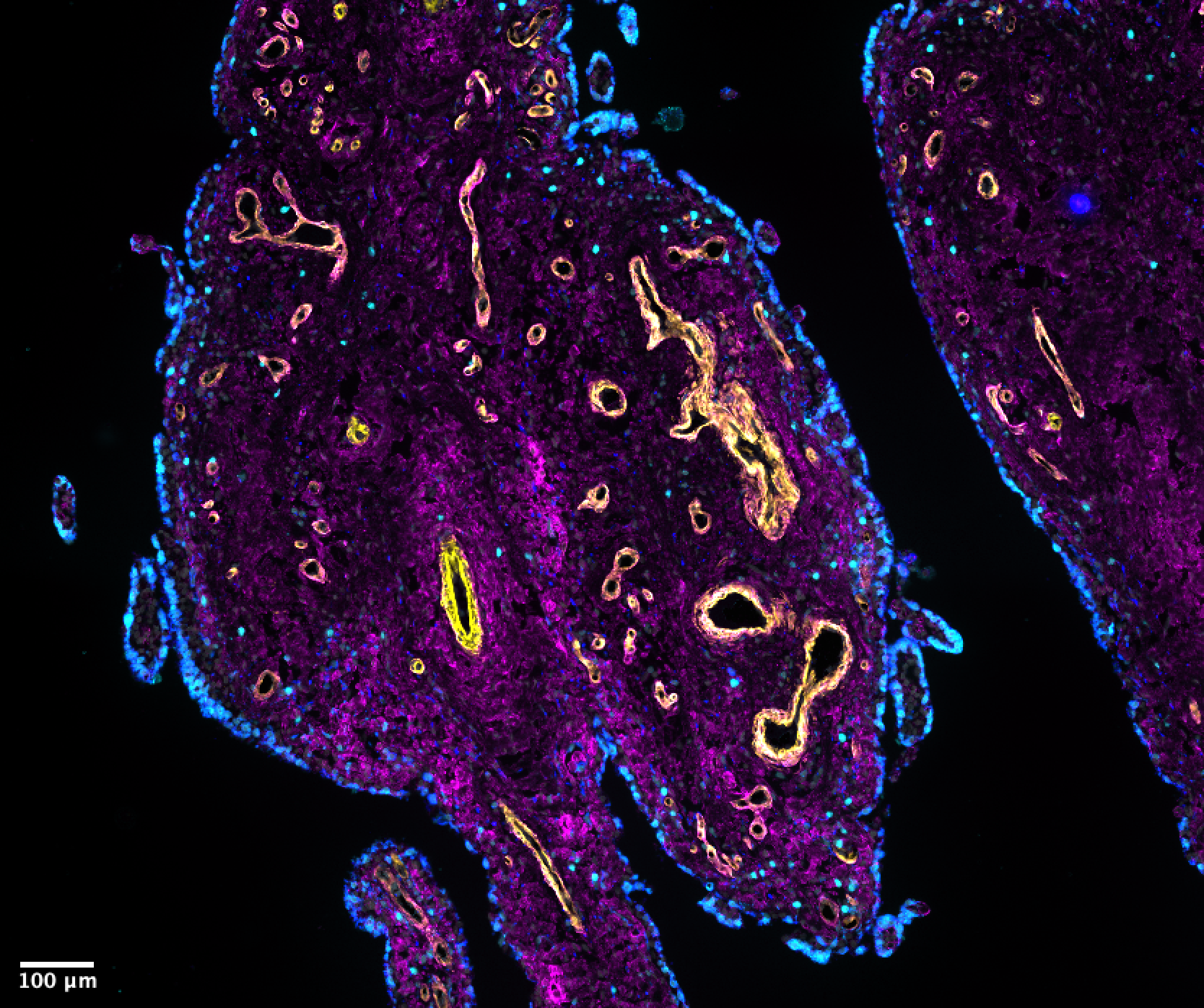

Supplement: Supplementary file 12 — Source data Fig. 5 [file 44320_2025_149_MOESM12_ESM.zip › Figure 5/5B/JRP144_HighOA_Fig5 (1, x=5732, y=8722, w=5032, h=4206).png]

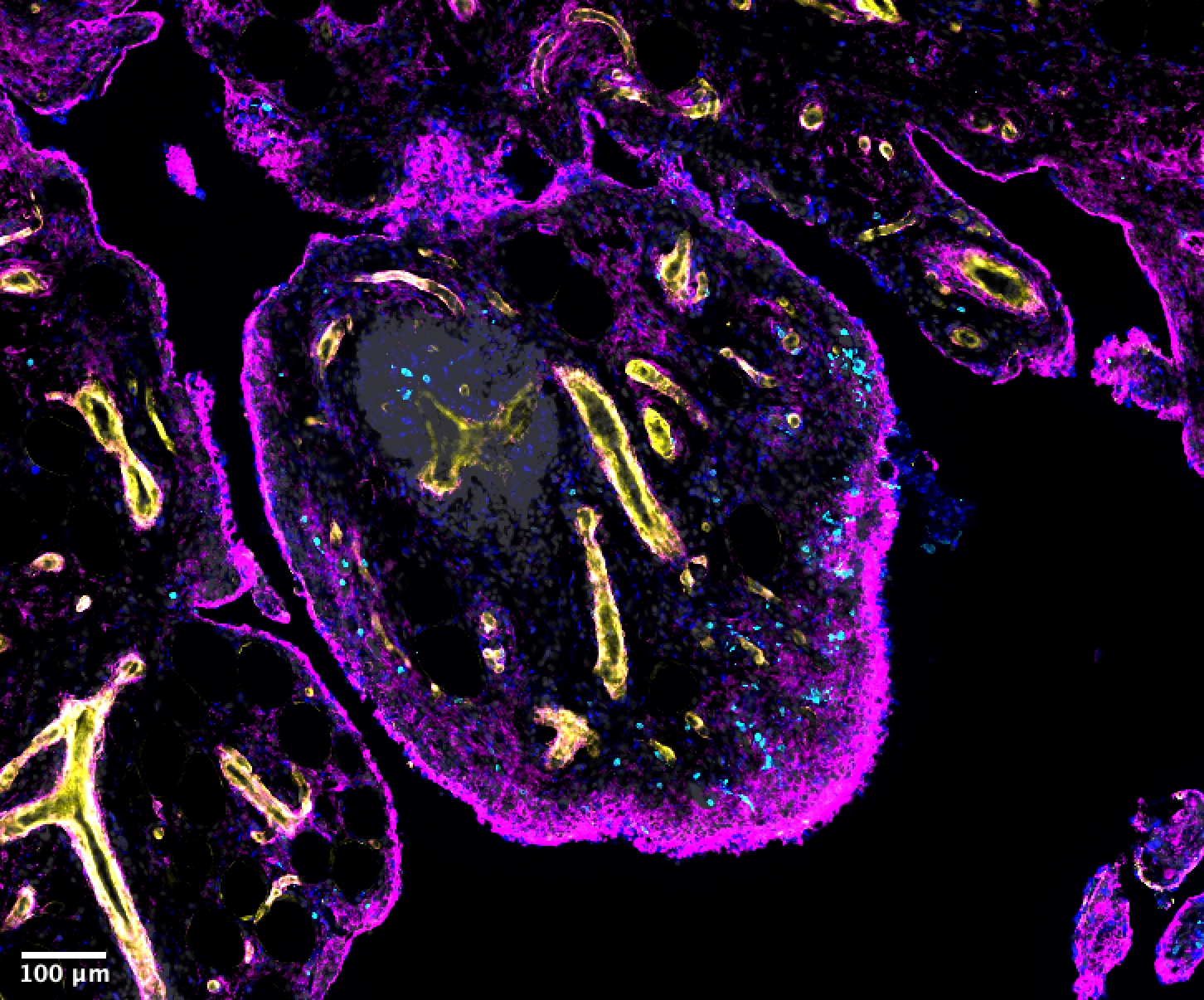

Supplement: Supplementary file 12 — Source data Fig. 5 [file 44320_2025_149_MOESM12_ESM.zip › Figure 5/5B/S00292623_LowOA_Fig5 (1, x=53566, y=13884, w=4381, h=3645).png]

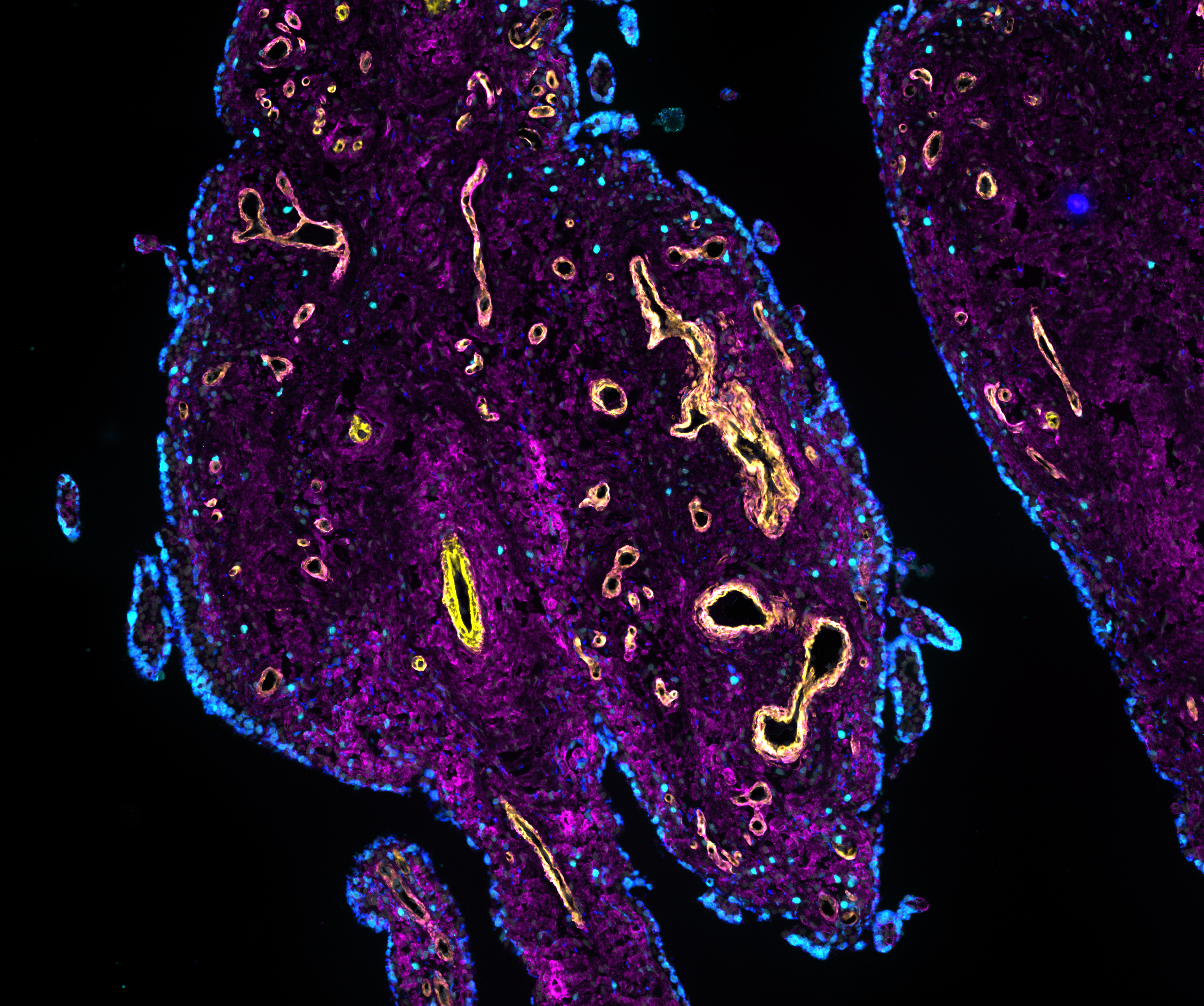

Supplement: Supplementary file 12 — Source data Fig. 5 [file 44320_2025_149_MOESM12_ESM.zip › Figure 5/5B/JRP144_HighOA_Fig5 (1, x=5732, y=8722, w=5032, h=4206).tif]

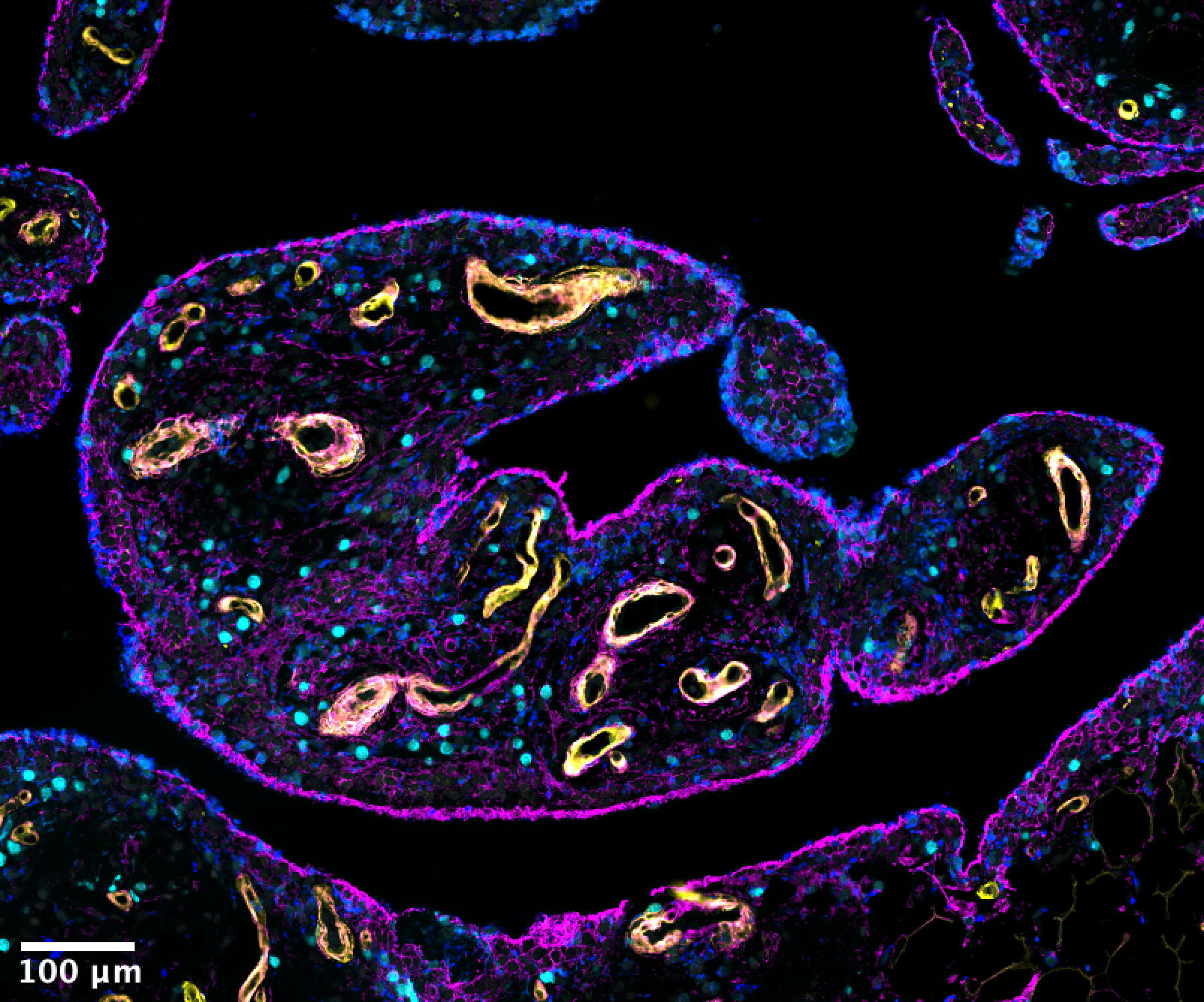

Supplement: Supplementary file 12 — Source data Fig. 5 [file 44320_2025_149_MOESM12_ESM.zip › Figure 5/5B/S00293087_MidOA_Fig5 (1, x=14310, y=10187, w=3239, h=2700).png]

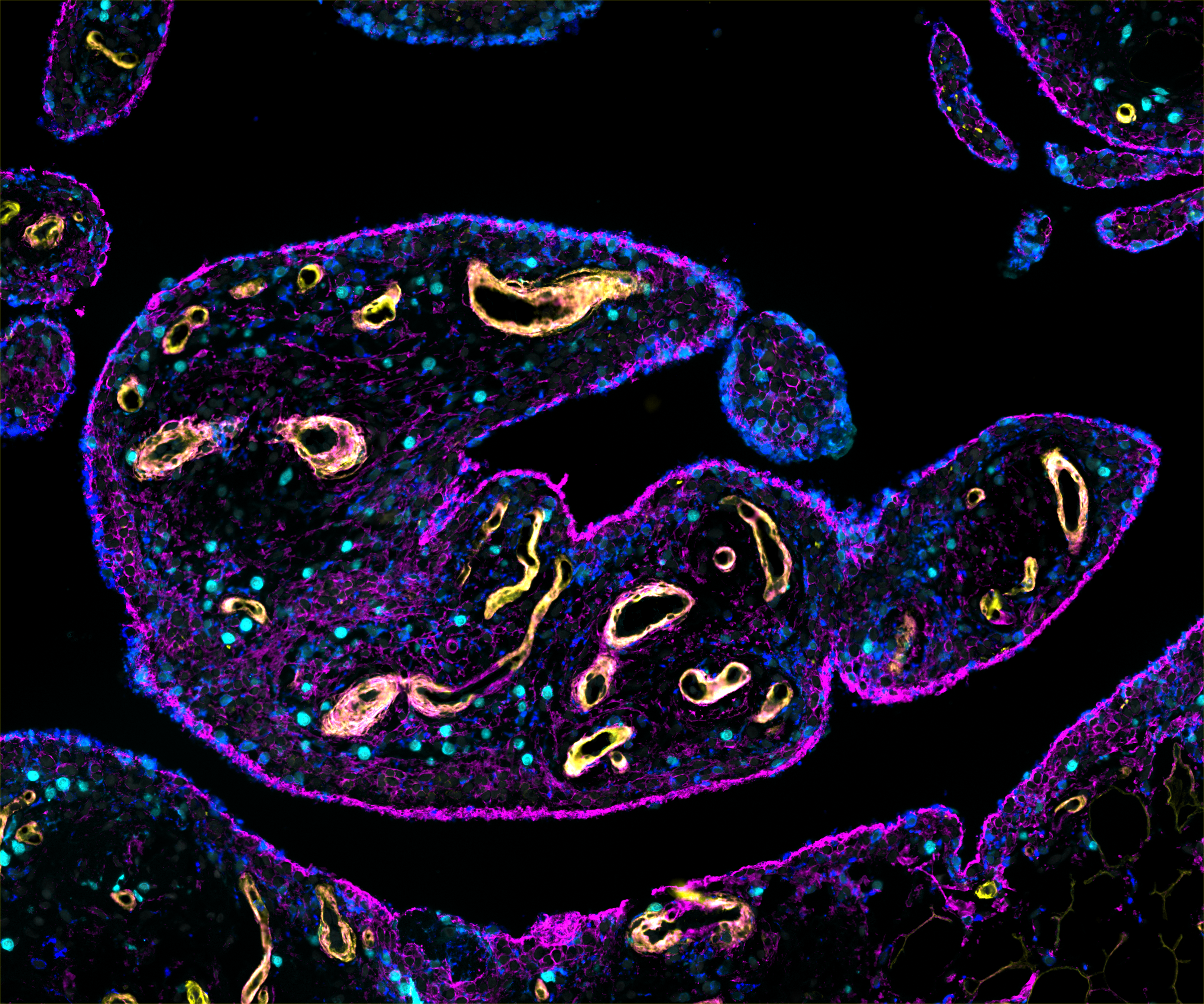

Supplement: Supplementary file 12 — Source data Fig. 5 [file 44320_2025_149_MOESM12_ESM.zip › Figure 5/5B/S00293087_MidOA_Fig5 (1, x=14310, y=10187, w=3239, h=2700).tif]

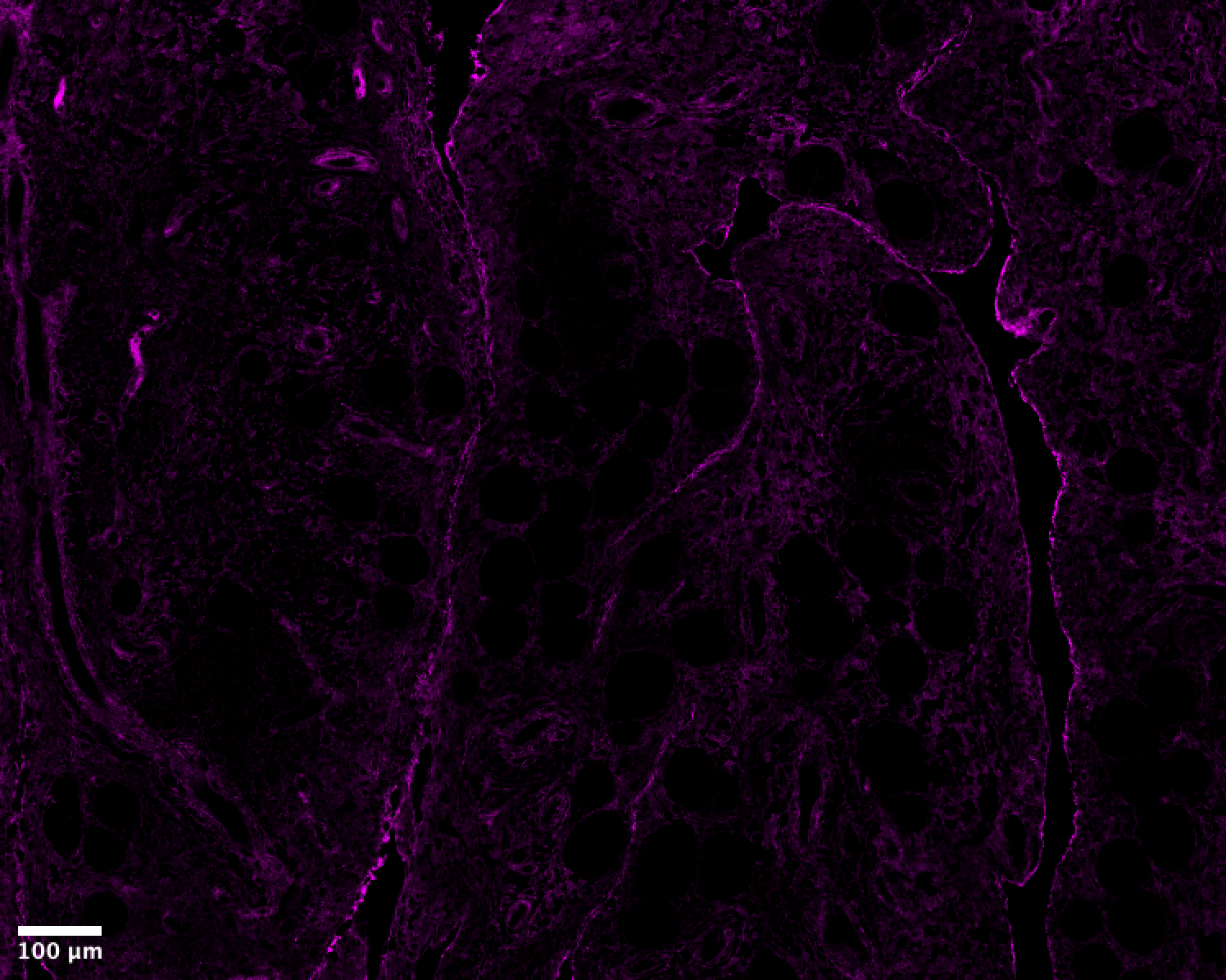

Supplement: Supplementary file 13 — Source data Fig. 6 [file 44320_2025_149_MOESM13_ESM.zip › Figure 6/6A-B/S00292623_OACOL6_Fig6 (1, x=5544, y=9829, w=4512, h=3606).png]

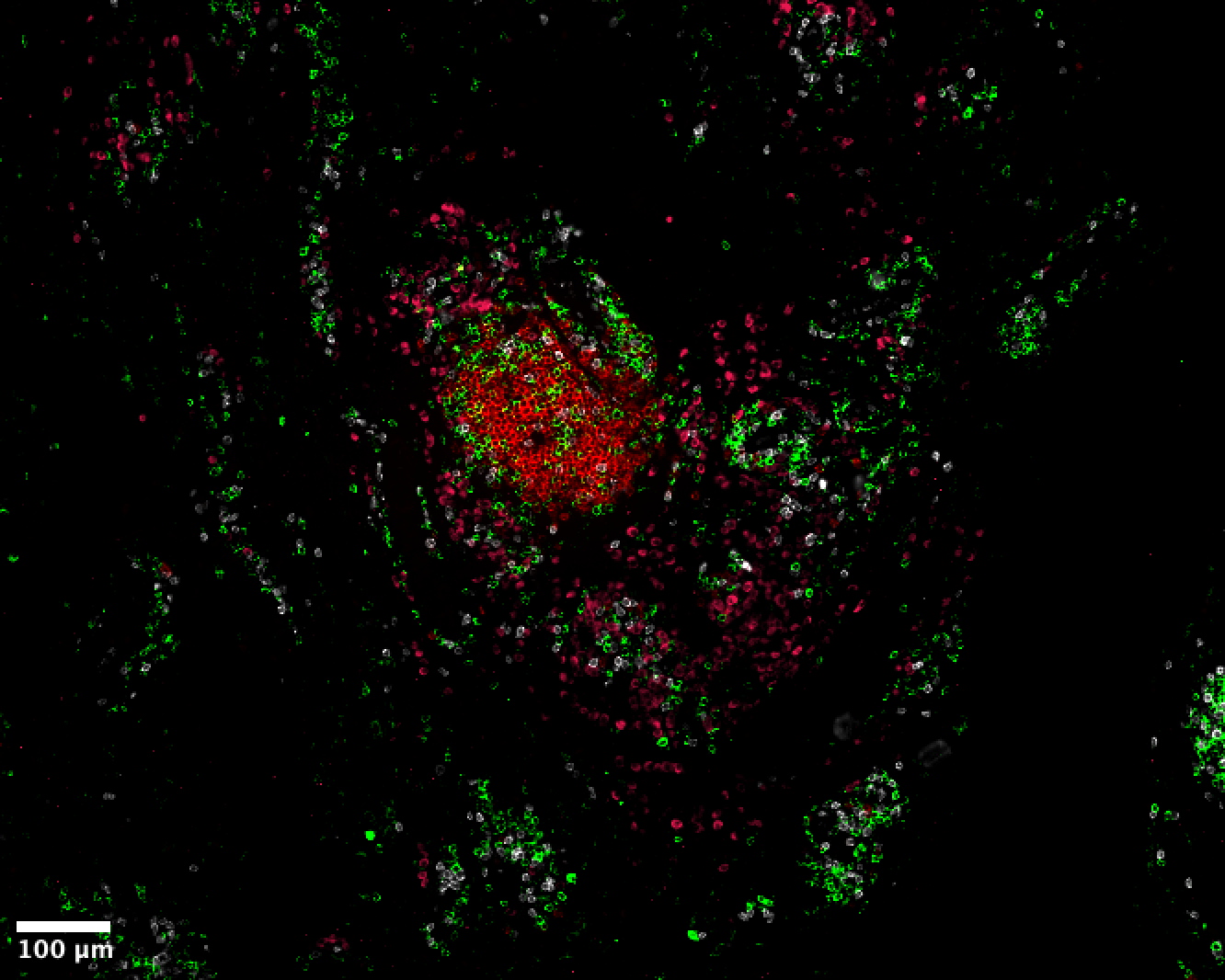

Supplement: Supplementary file 13 — Source data Fig. 6 [file 44320_2025_149_MOESM13_ESM.zip › Figure 6/6A-B/JRP141_RALympho_Fig6 (1, x=32530, y=3615, w=4024, h=3212).png]

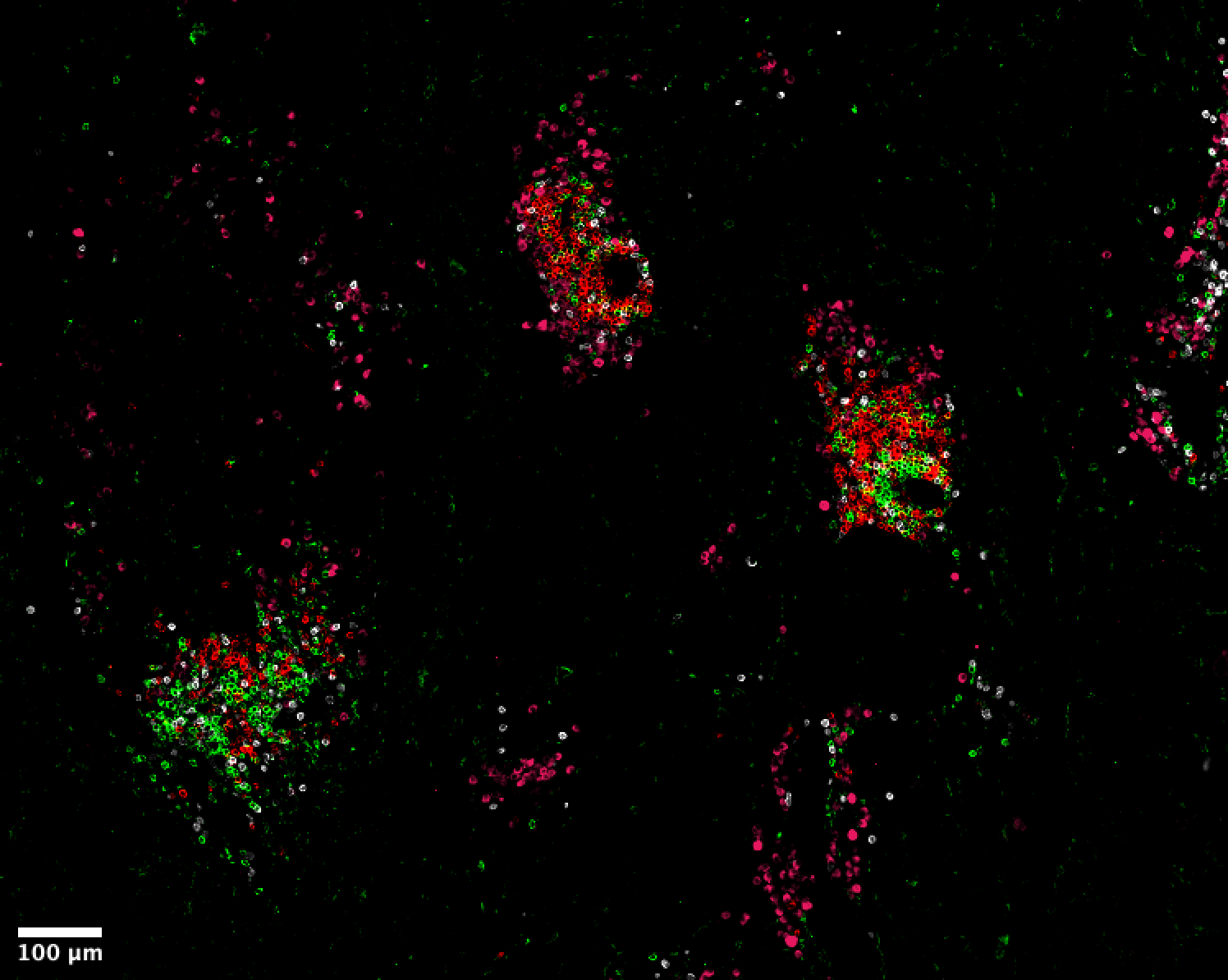

Supplement: Supplementary file 13 — Source data Fig. 6 [file 44320_2025_149_MOESM13_ESM.zip › Figure 6/6A-B/S00292623_OALympho_Fig6 (1, x=5544, y=9829, w=4512, h=3606).png]

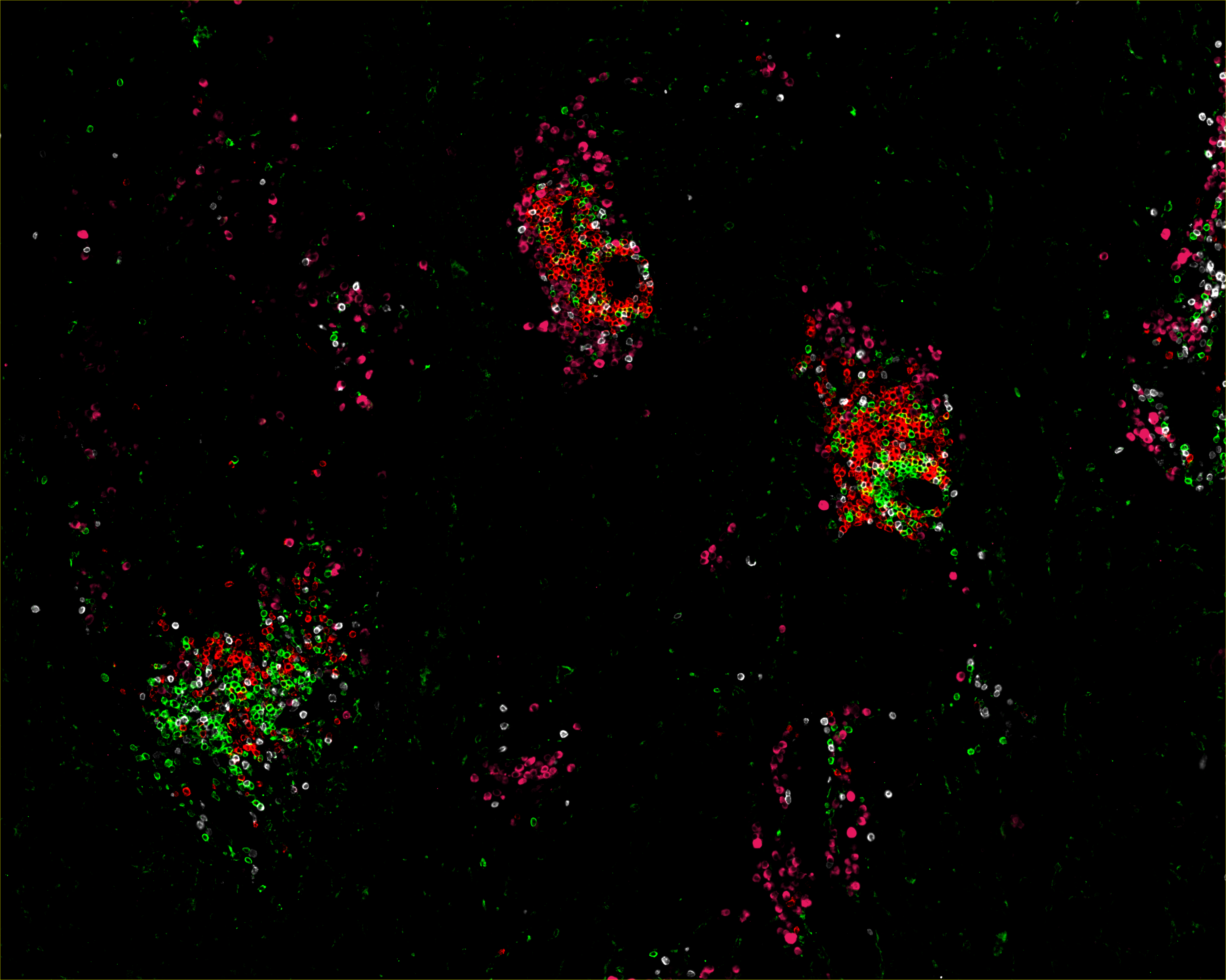

Supplement: Supplementary file 13 — Source data Fig. 6 [file 44320_2025_149_MOESM13_ESM.zip › Figure 6/6A-B/S00292623_OALympho_Fig6 (1, x=5544, y=9829, w=4512, h=3606).tif]

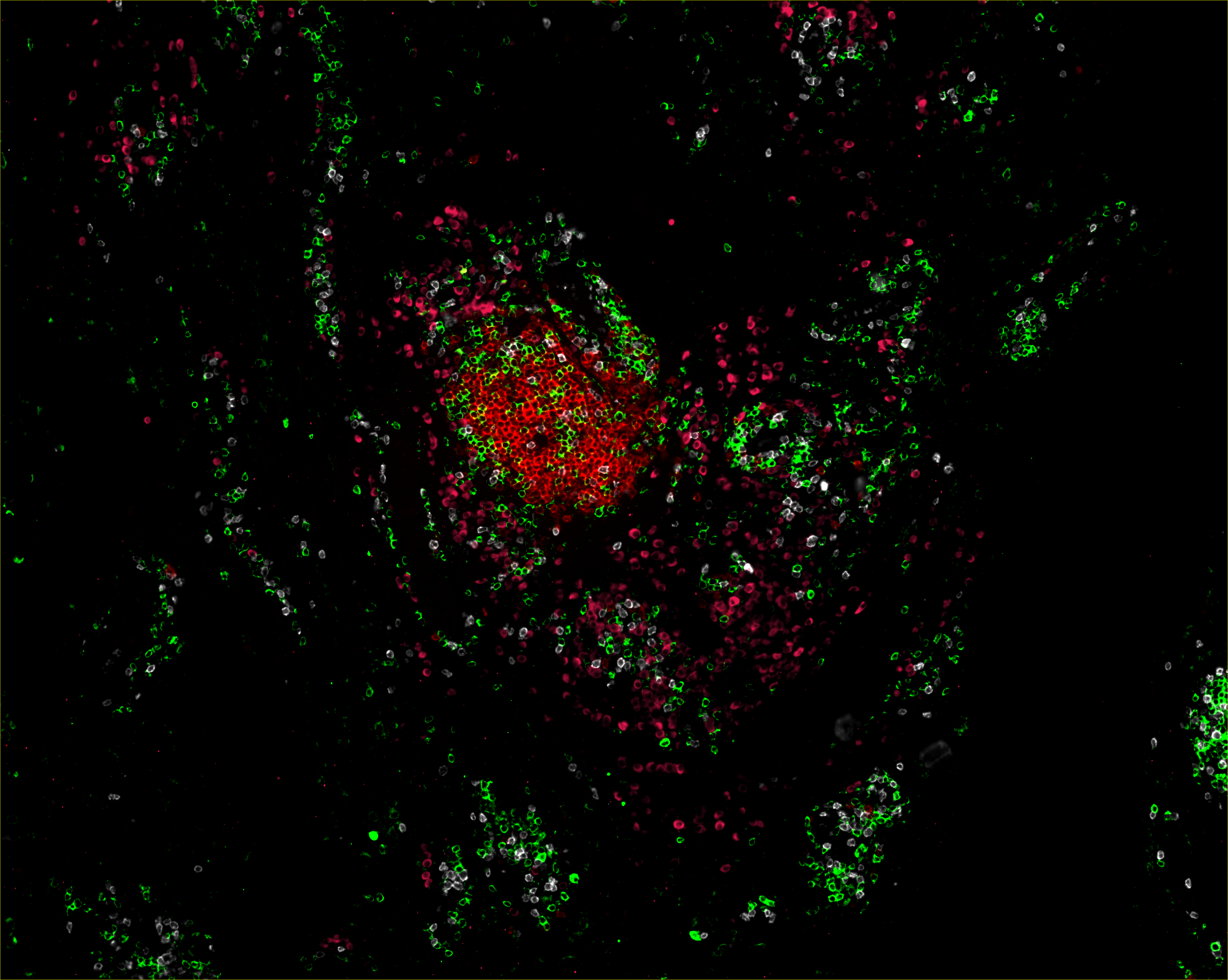

Supplement: Supplementary file 13 — Source data Fig. 6 [file 44320_2025_149_MOESM13_ESM.zip › Figure 6/6A-B/JRP141_RALympho_Fig6 (1, x=32530, y=3615, w=4024, h=3212).tif]

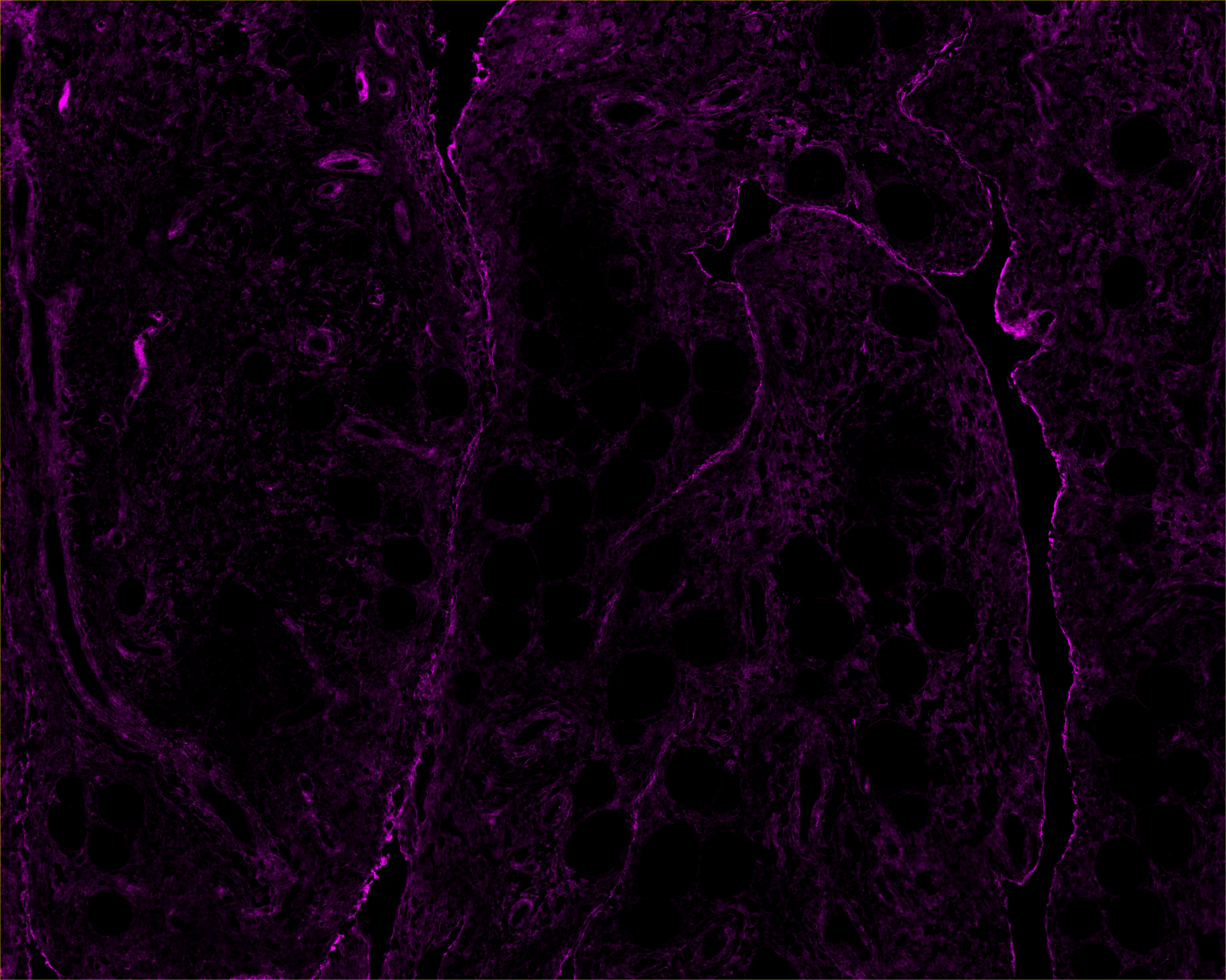

Supplement: Supplementary file 13 — Source data Fig. 6 [file 44320_2025_149_MOESM13_ESM.zip › Figure 6/6A-B/S00292623_OACOL6_Fig6 (1, x=5544, y=9829, w=4512, h=3606).tif]

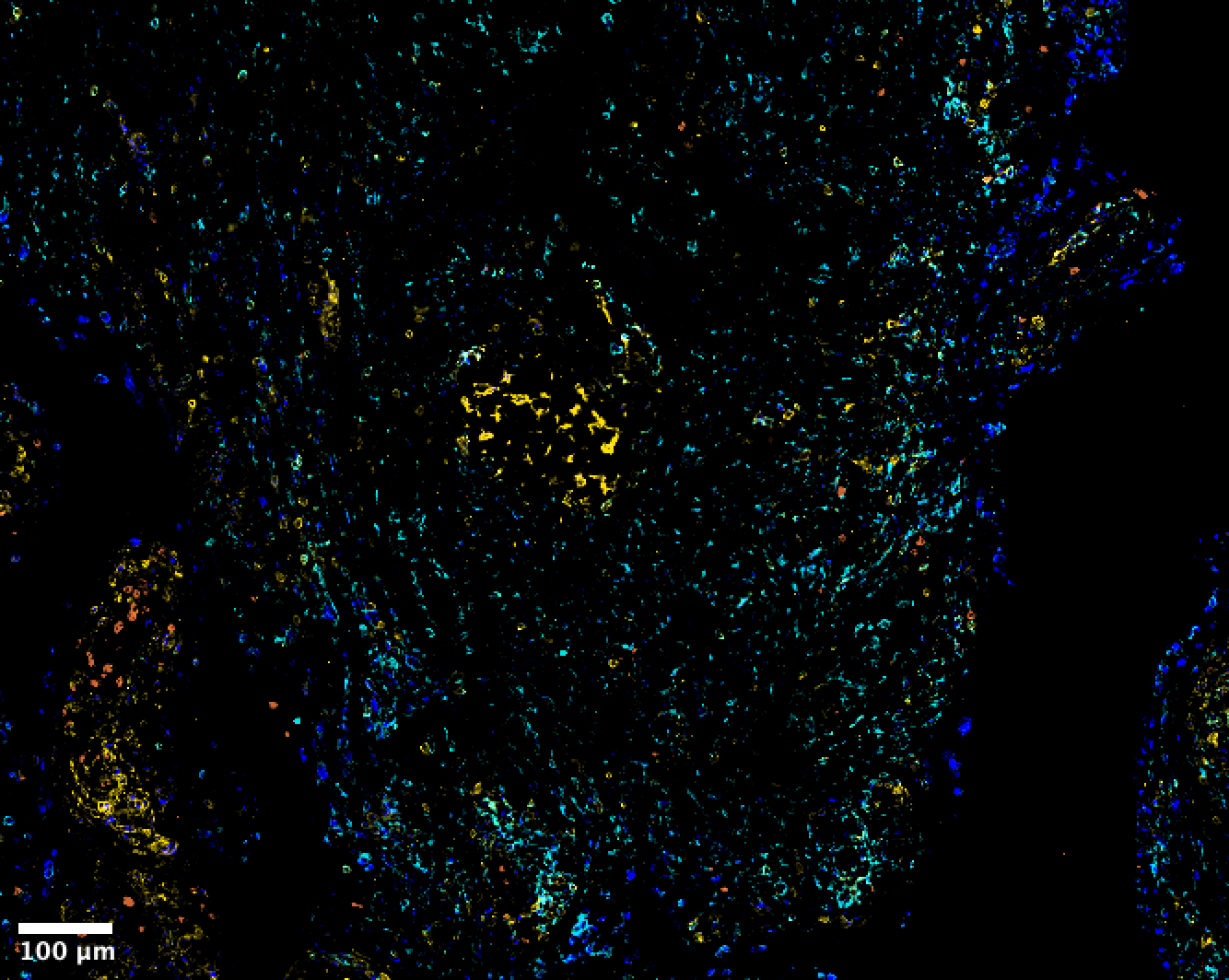

Supplement: Supplementary file 13 — Source data Fig. 6 [file 44320_2025_149_MOESM13_ESM.zip › Figure 6/6A-B/JRP141_RAMyeloid_Fig6 (1, x=32530, y=3615, w=4024, h=3212).png]

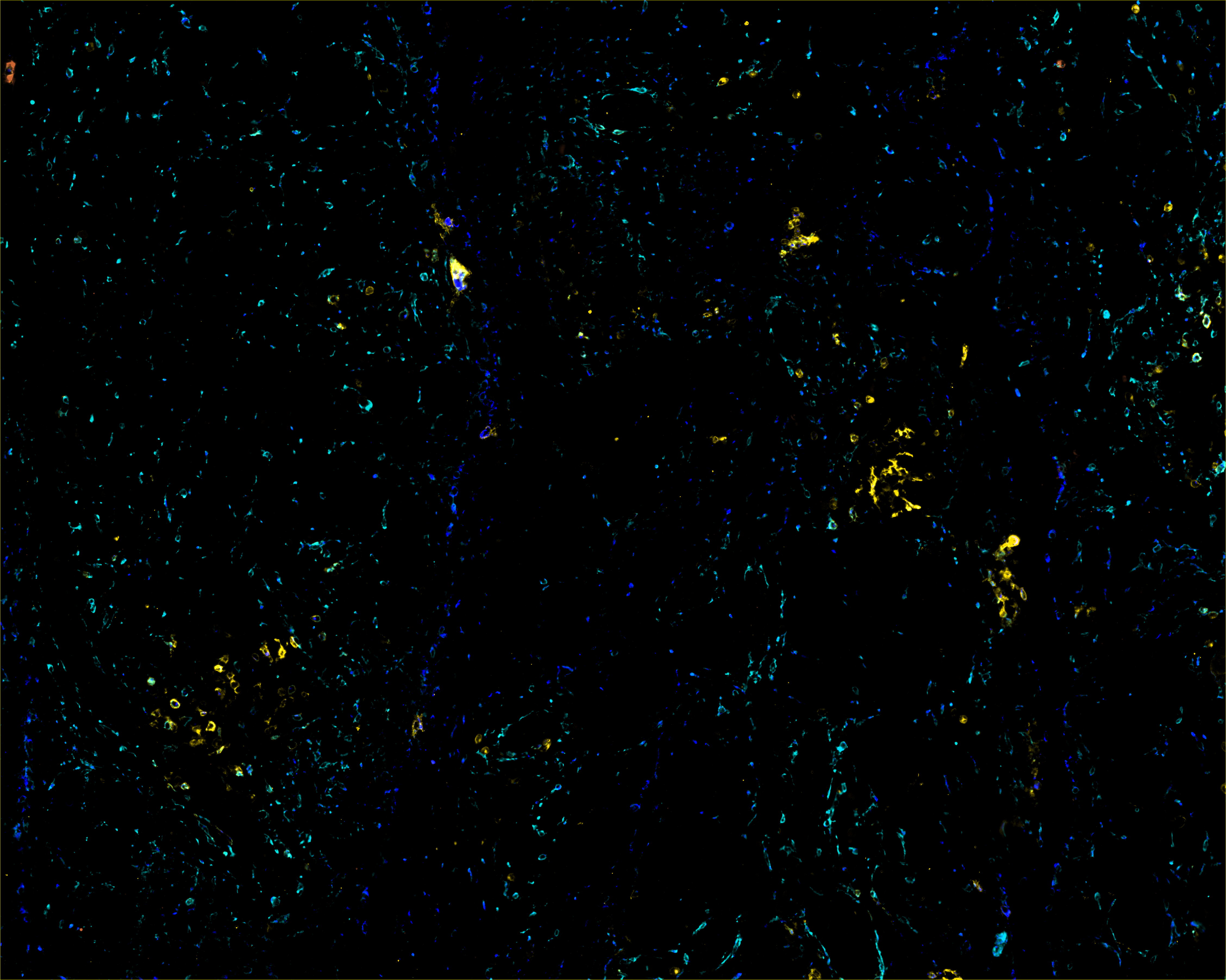

Supplement: Supplementary file 13 — Source data Fig. 6 [file 44320_2025_149_MOESM13_ESM.zip › Figure 6/6A-B/S00292623_OAMyeloid_Fig6 (1, x=5544, y=9829, w=4512, h=3606).tif]

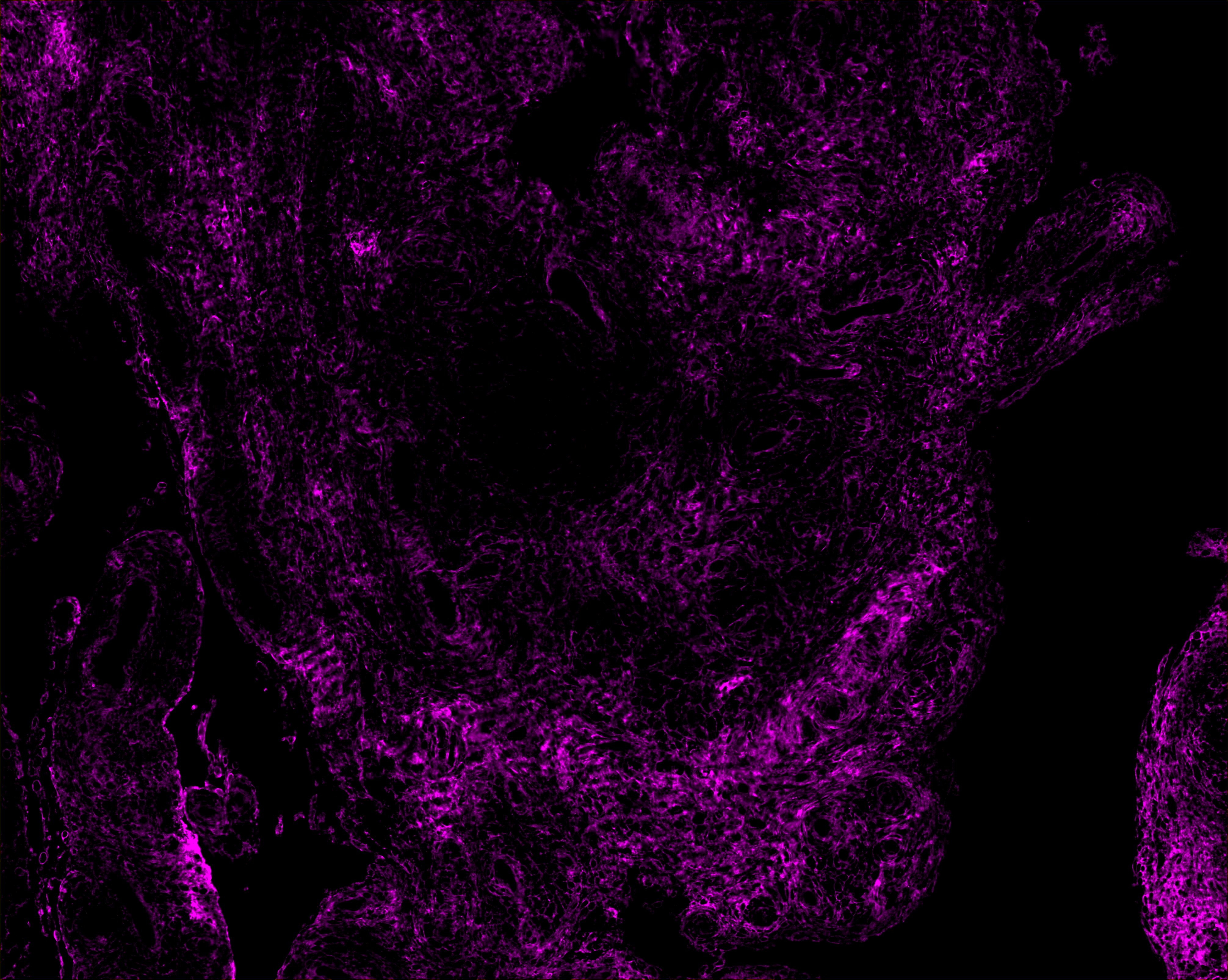

Supplement: Supplementary file 13 — Source data Fig. 6 [file 44320_2025_149_MOESM13_ESM.zip › Figure 6/6A-B/JRP141_RACOL6_Fig6 (1, x=32530, y=3615, w=4024, h=3212).tif]

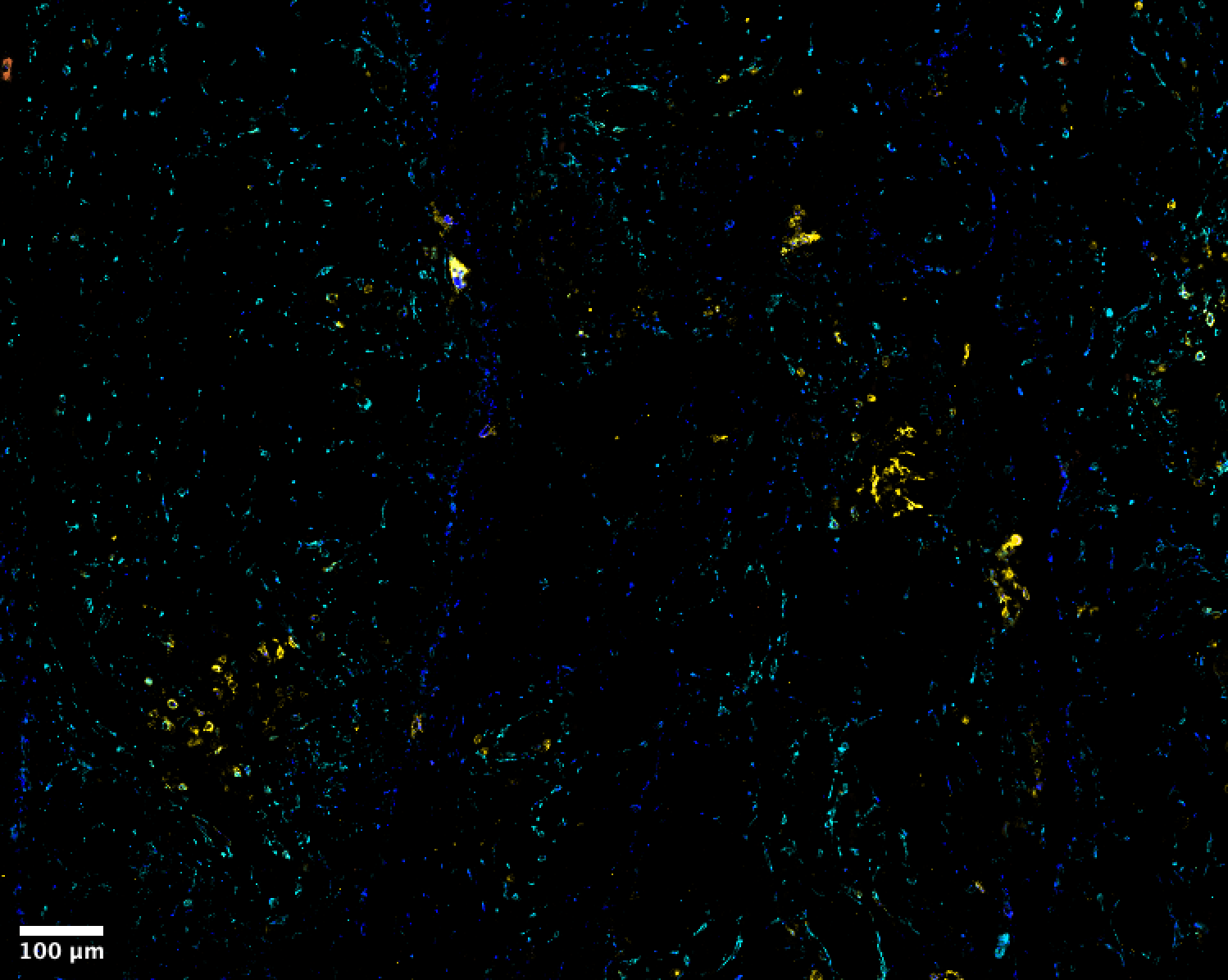

Supplement: Supplementary file 13 — Source data Fig. 6 [file 44320_2025_149_MOESM13_ESM.zip › Figure 6/6A-B/S00292623_OAMyeloid_Fig6 (1, x=5544, y=9829, w=4512, h=3606).png]

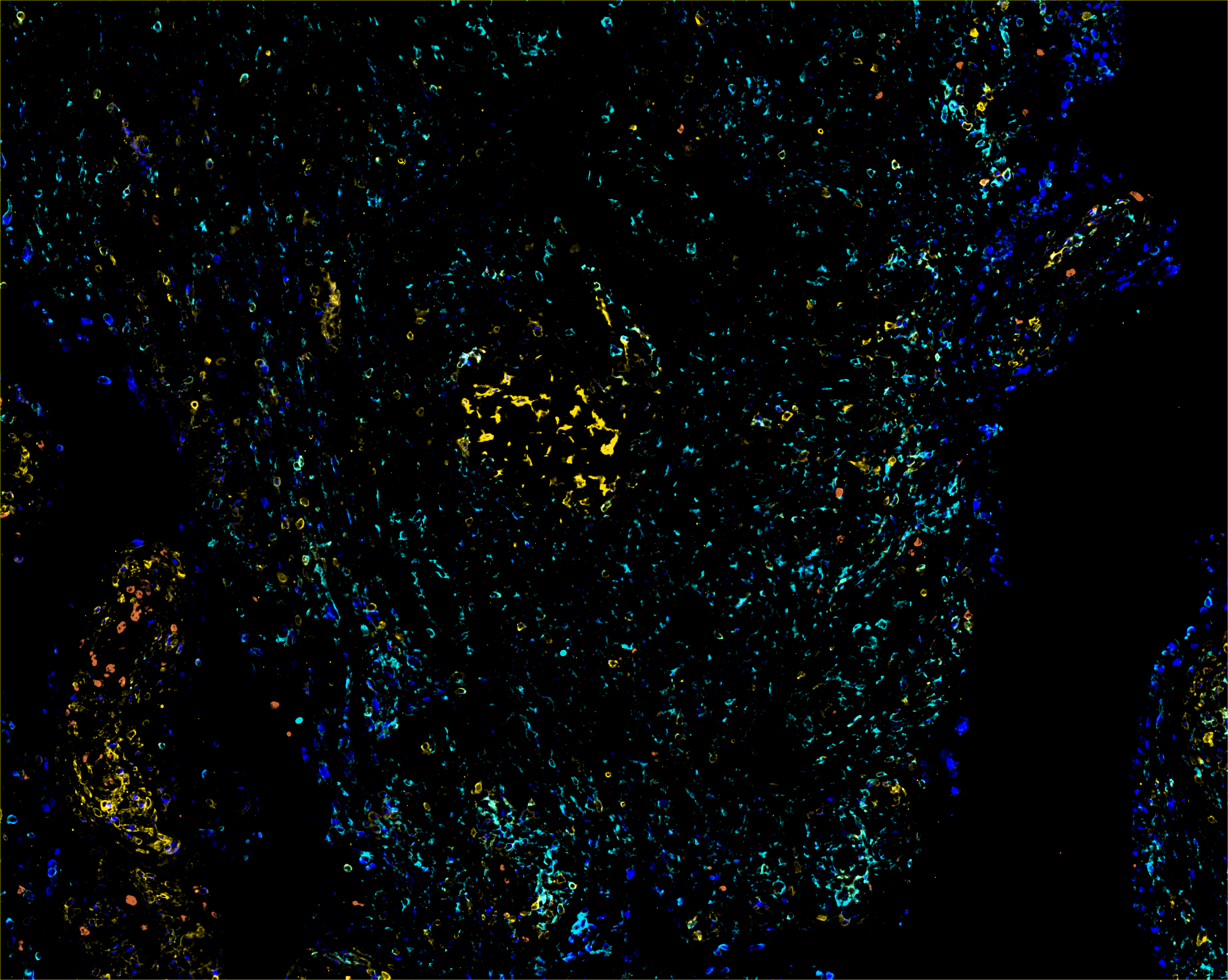

Supplement: Supplementary file 13 — Source data Fig. 6 [file 44320_2025_149_MOESM13_ESM.zip › Figure 6/6A-B/JRP141_RAMyeloid_Fig6 (1, x=32530, y=3615, w=4024, h=3212).tif]

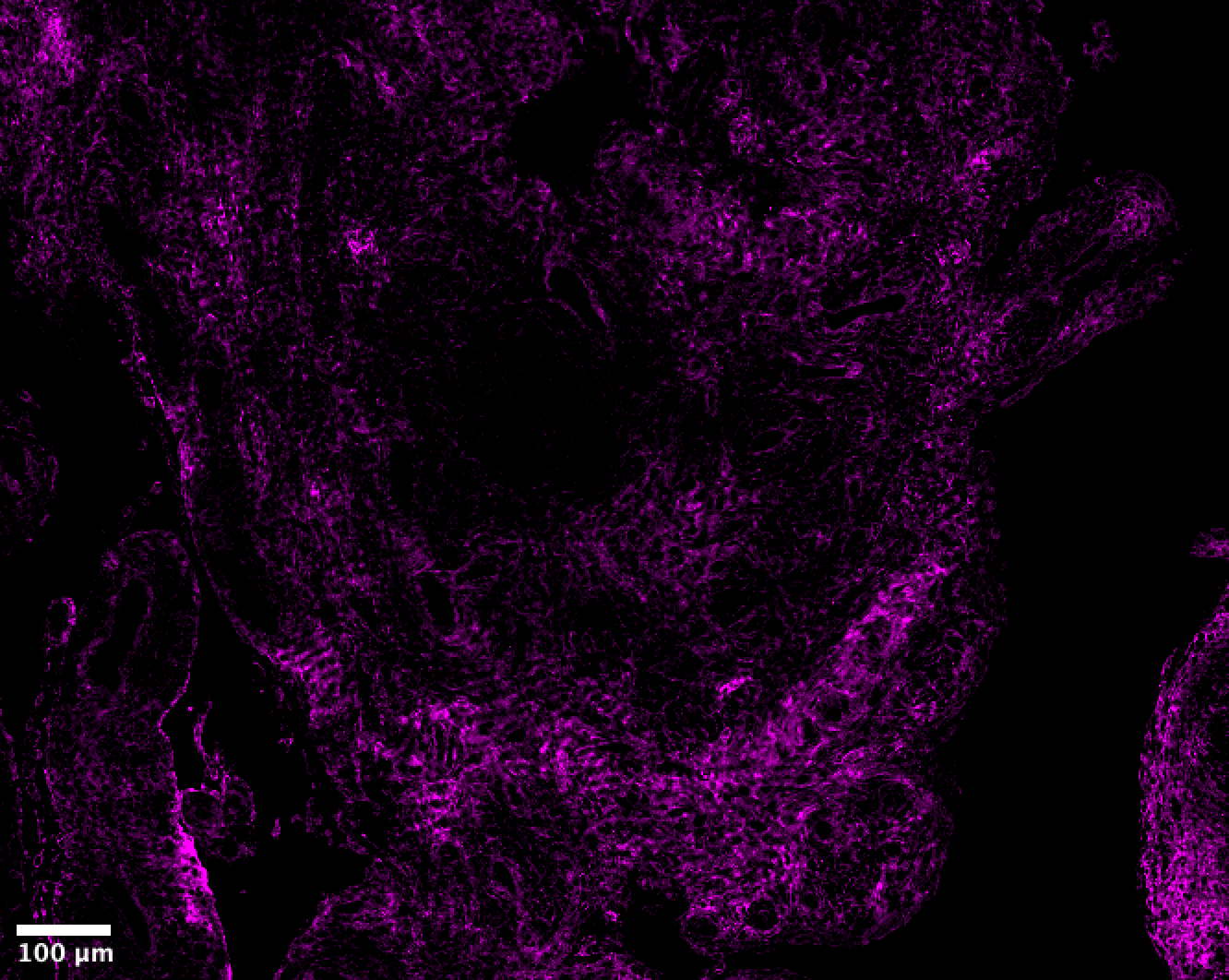

Supplement: Supplementary file 13 — Source data Fig. 6 [file 44320_2025_149_MOESM13_ESM.zip › Figure 6/6A-B/JRP141_RACOL6_Fig6 (1, x=32530, y=3615, w=4024, h=3212).png]

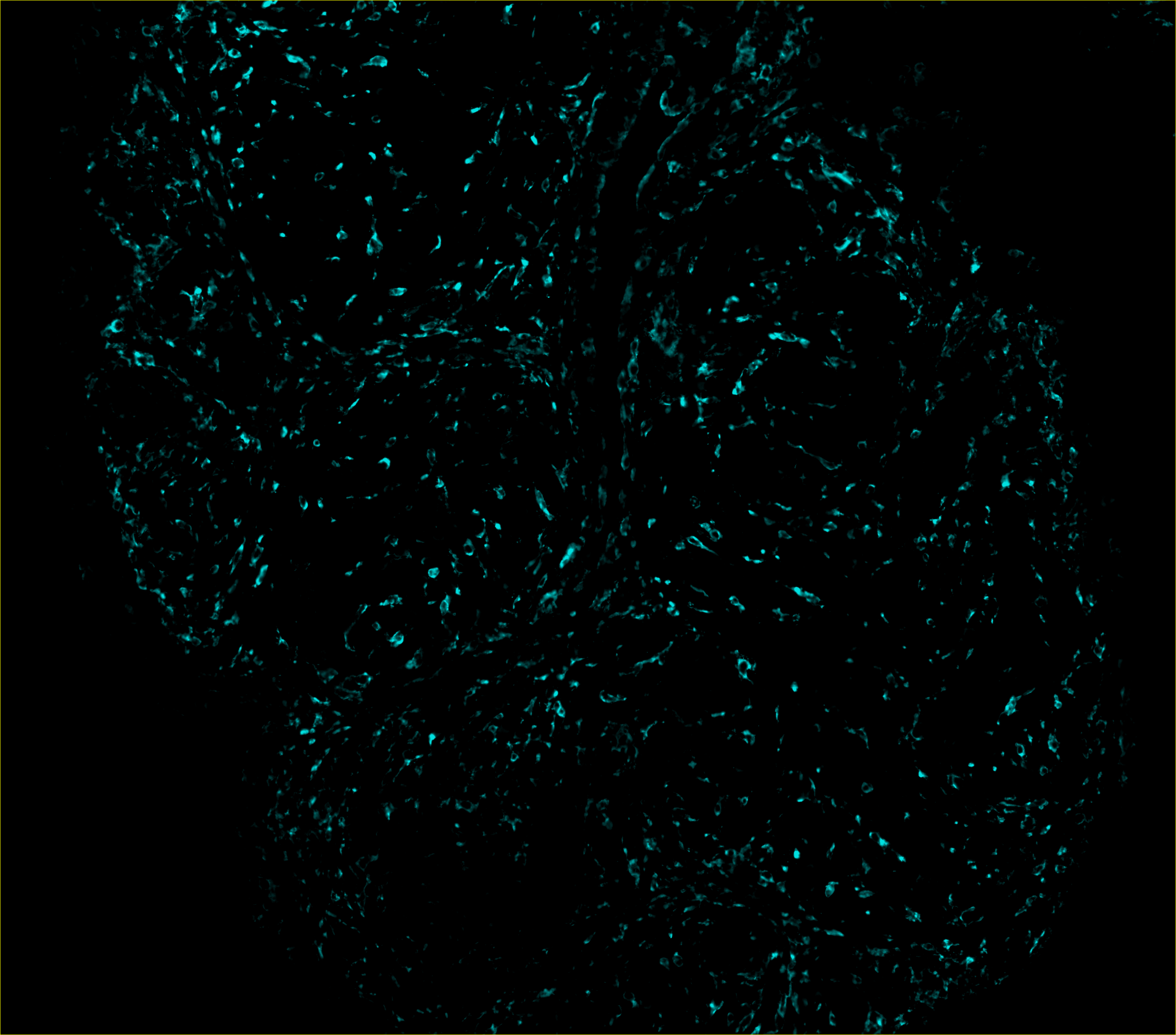

Supplement: Supplementary file 14 — Source data Fig. 7 [file 44320_2025_149_MOESM14_ESM.zip › Figure 7/7A/JRP122_CD14_Fig7 (1, x=1755, y=4032, w=2737, h=2410).tif]

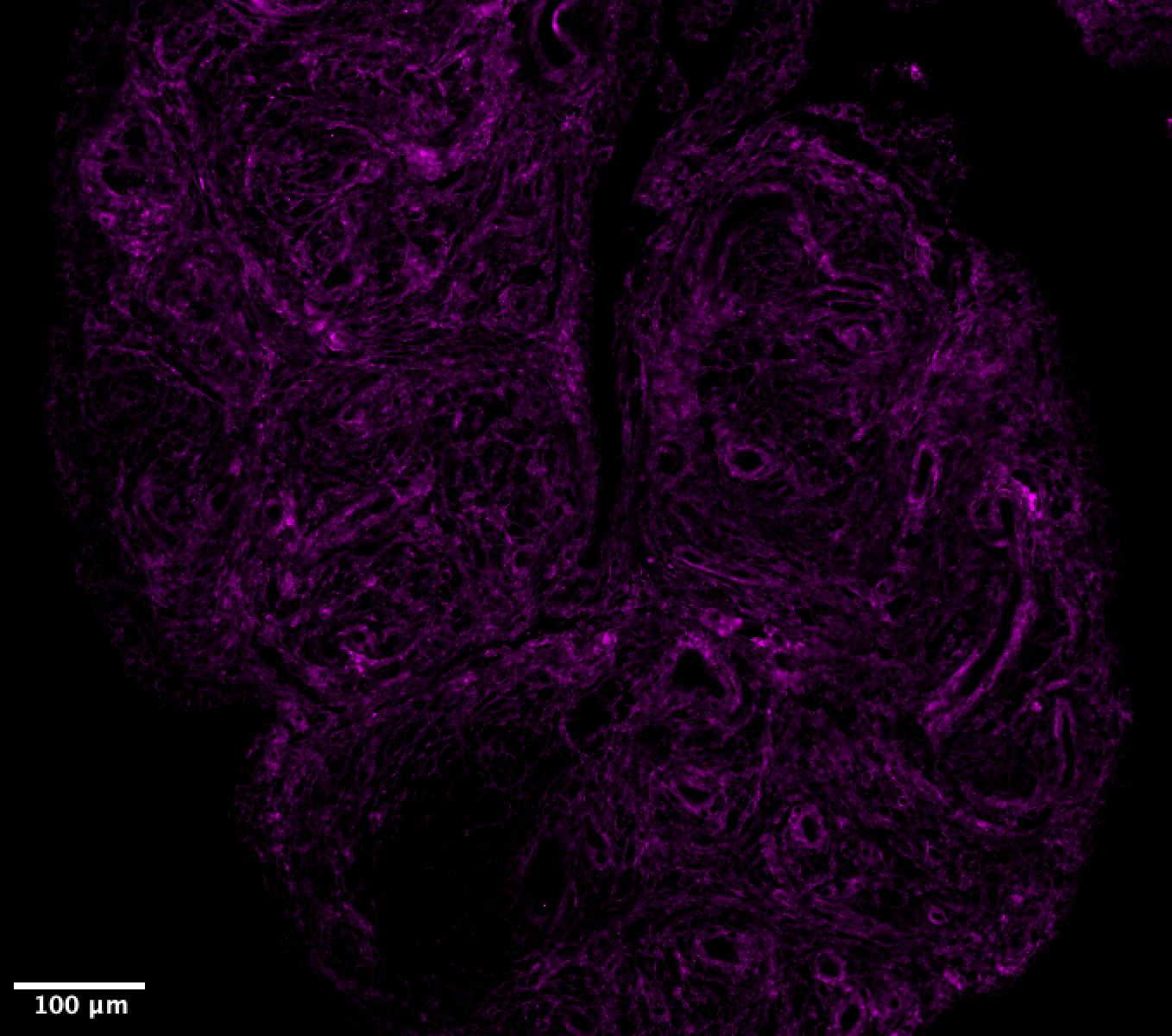

Supplement: Supplementary file 14 — Source data Fig. 7 [file 44320_2025_149_MOESM14_ESM.zip › Figure 7/7A/JRP122_COL6_Fig7 (1, x=1755, y=4032, w=2737, h=2410).png]

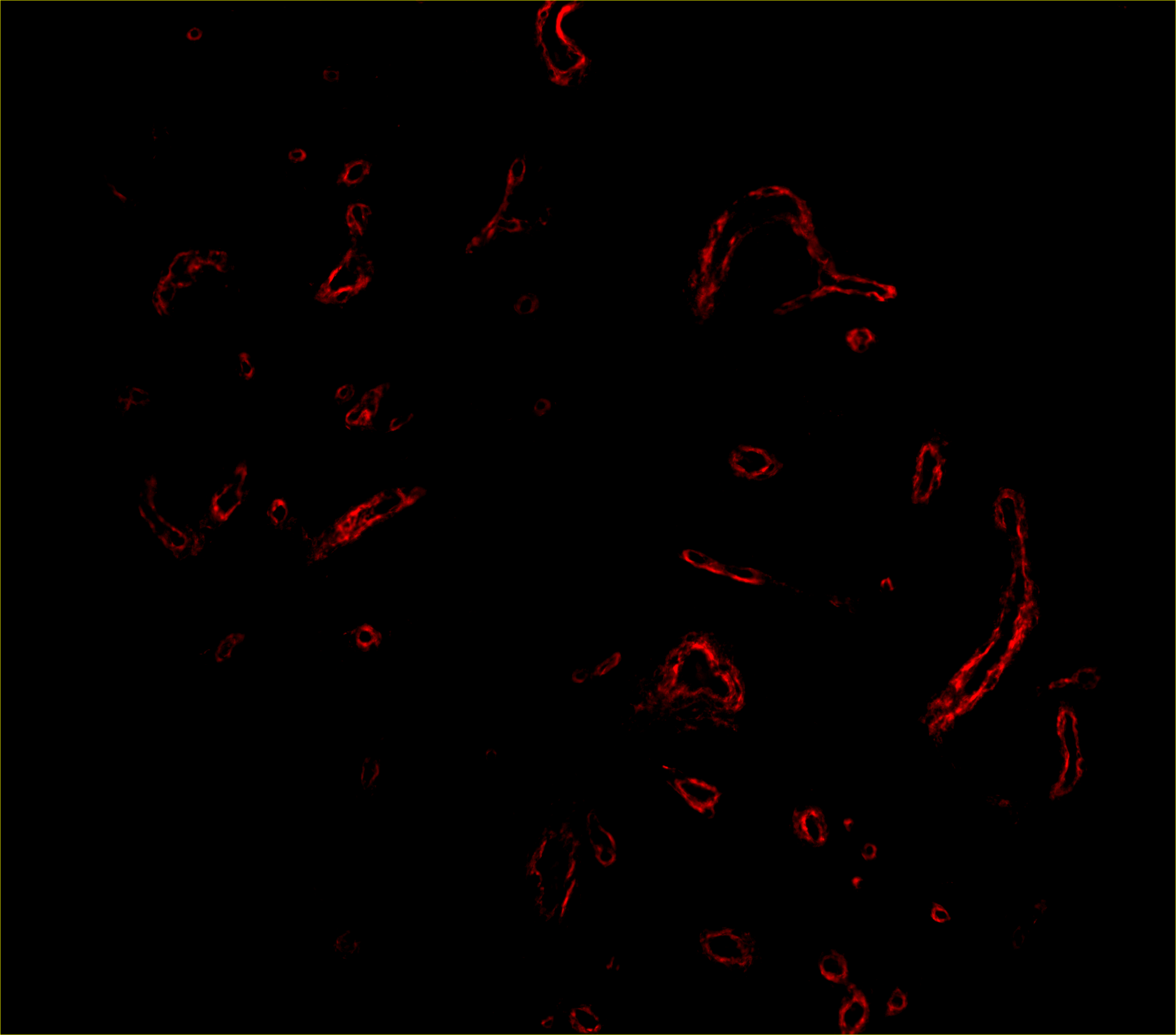

Supplement: Supplementary file 14 — Source data Fig. 7 [file 44320_2025_149_MOESM14_ESM.zip › Figure 7/7A/JRP122_COL4_Fig7 (1, x=1755, y=4032, w=2737, h=2410).tif]

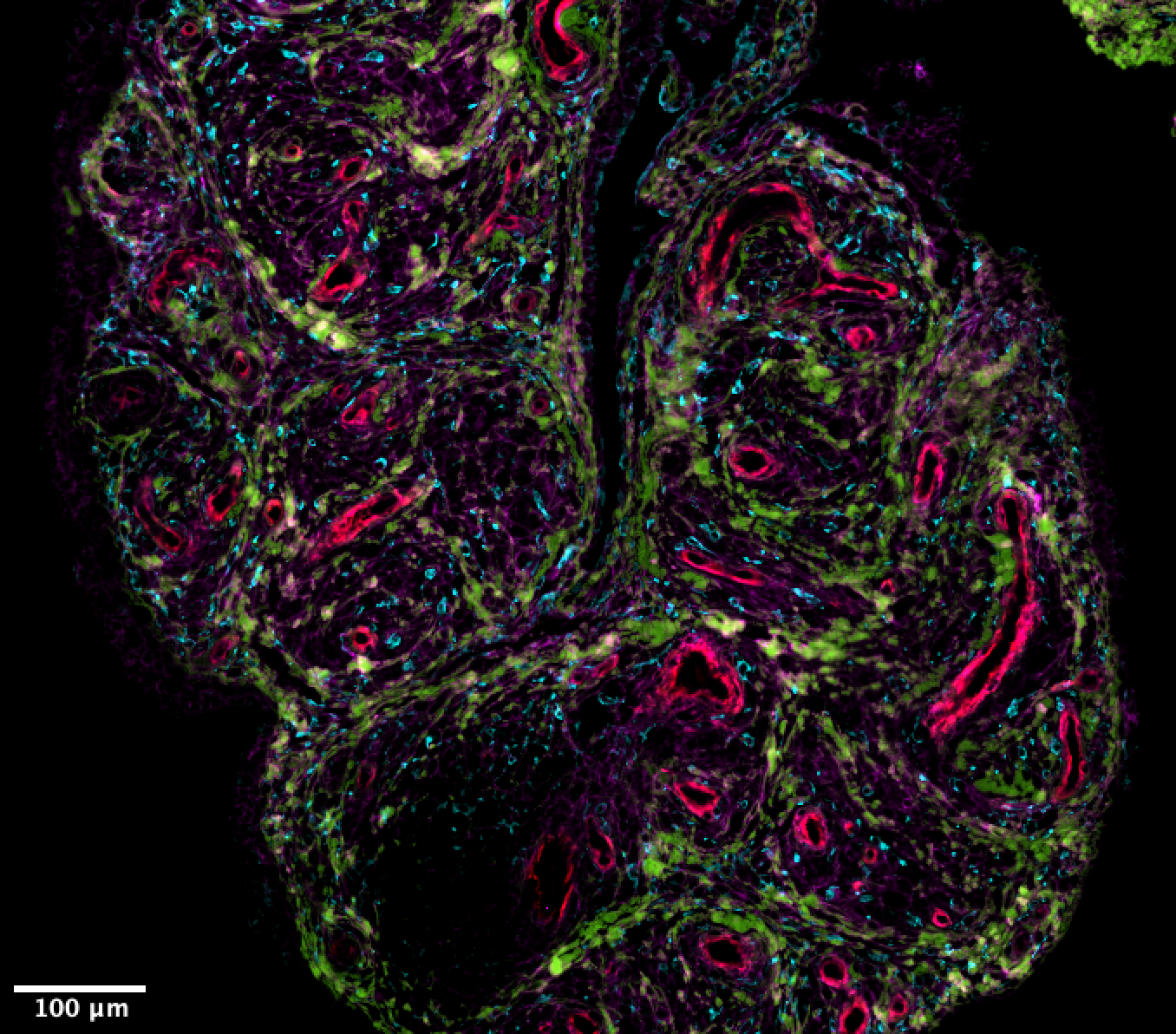

Supplement: Supplementary file 14 — Source data Fig. 7 [file 44320_2025_149_MOESM14_ESM.zip › Figure 7/7A/JRP122_Full_Fig7 (1, x=1755, y=4032, w=2737, h=2410).png]

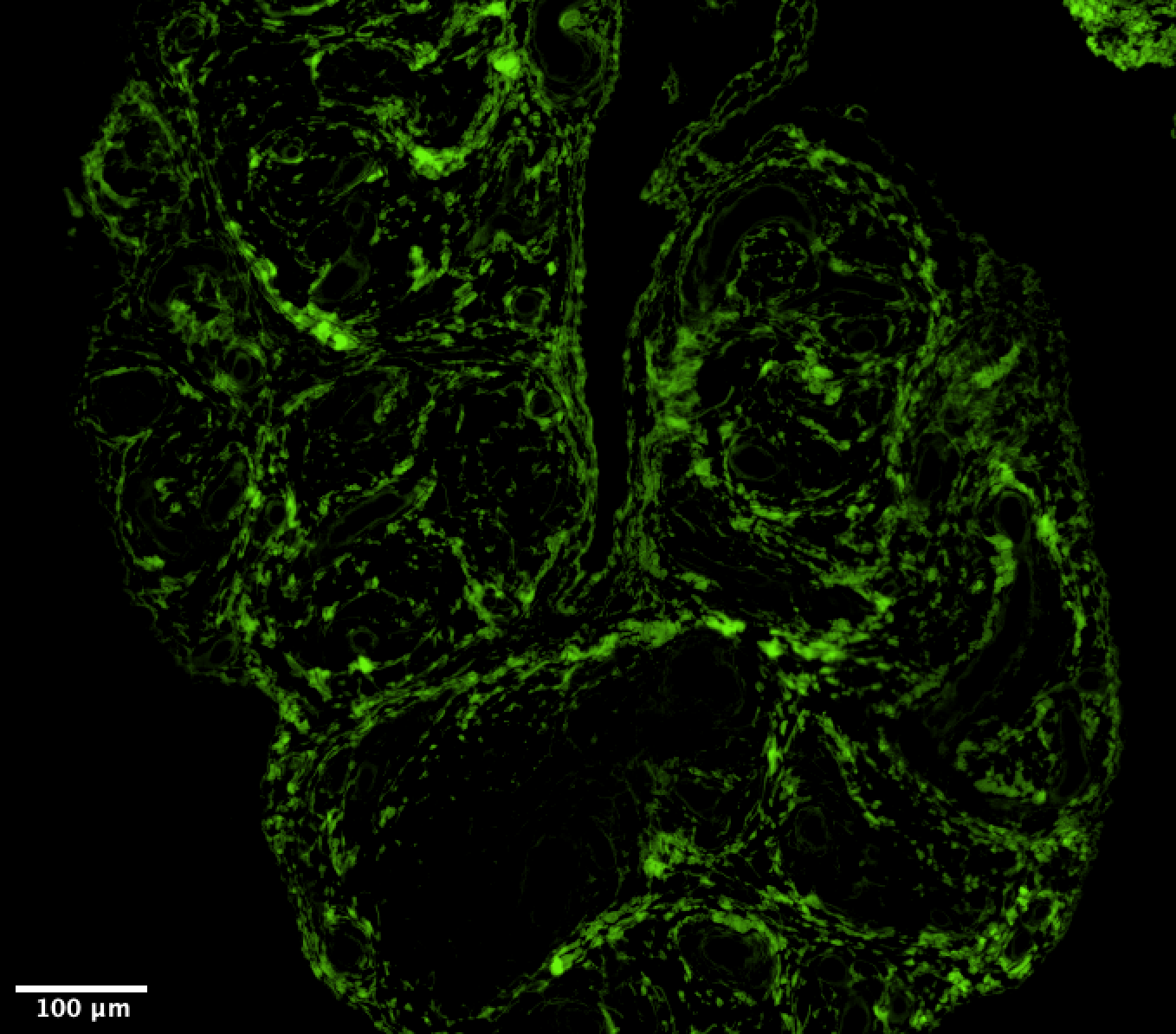

Supplement: Supplementary file 14 — Source data Fig. 7 [file 44320_2025_149_MOESM14_ESM.zip › Figure 7/7A/JRP122_CHP_Fig7 (1, x=1755, y=4032, w=2737, h=2410).png]

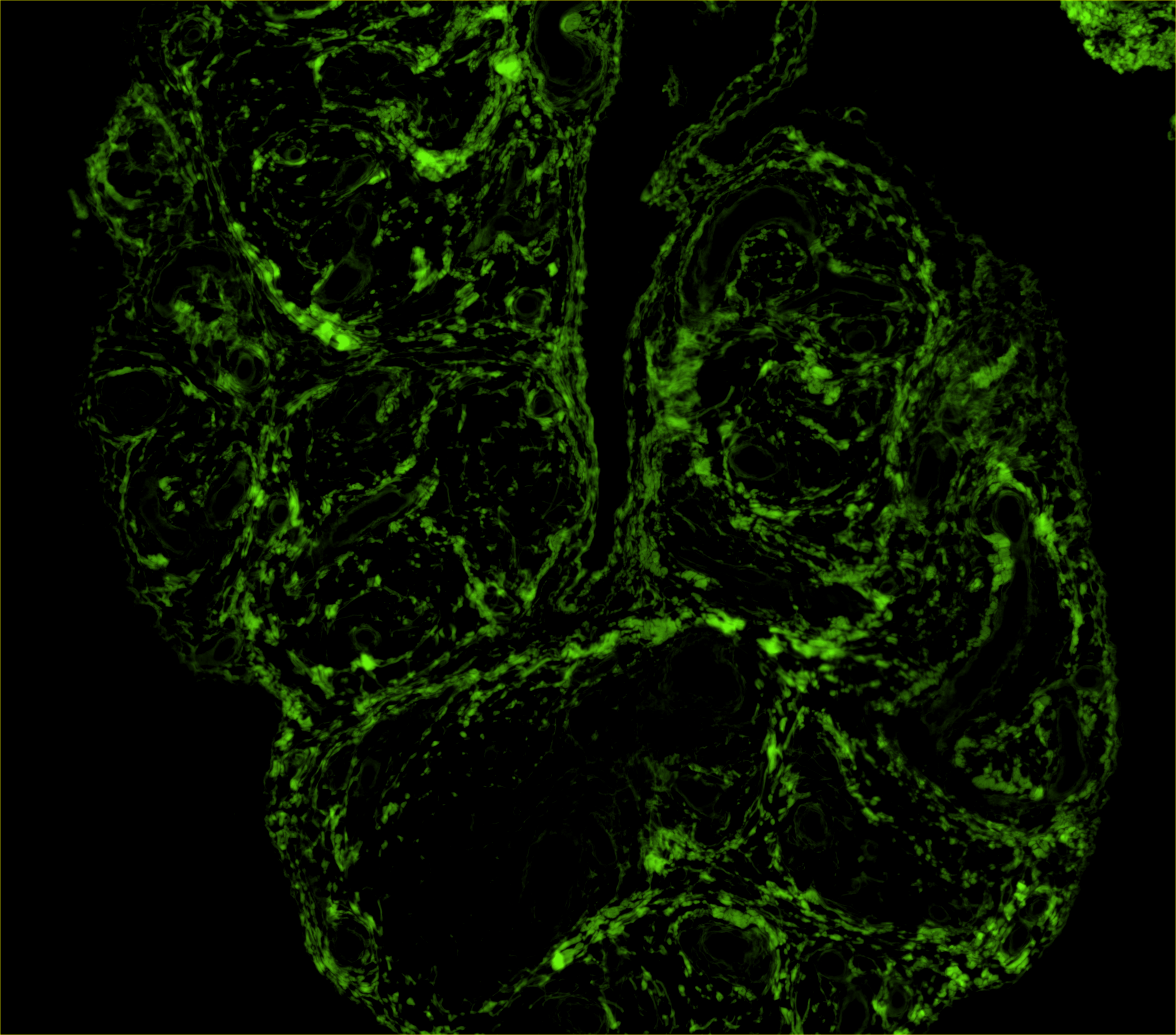

Supplement: Supplementary file 14 — Source data Fig. 7 [file 44320_2025_149_MOESM14_ESM.zip › Figure 7/7A/JRP122_CHP_Fig7 (1, x=1755, y=4032, w=2737, h=2410).tif]

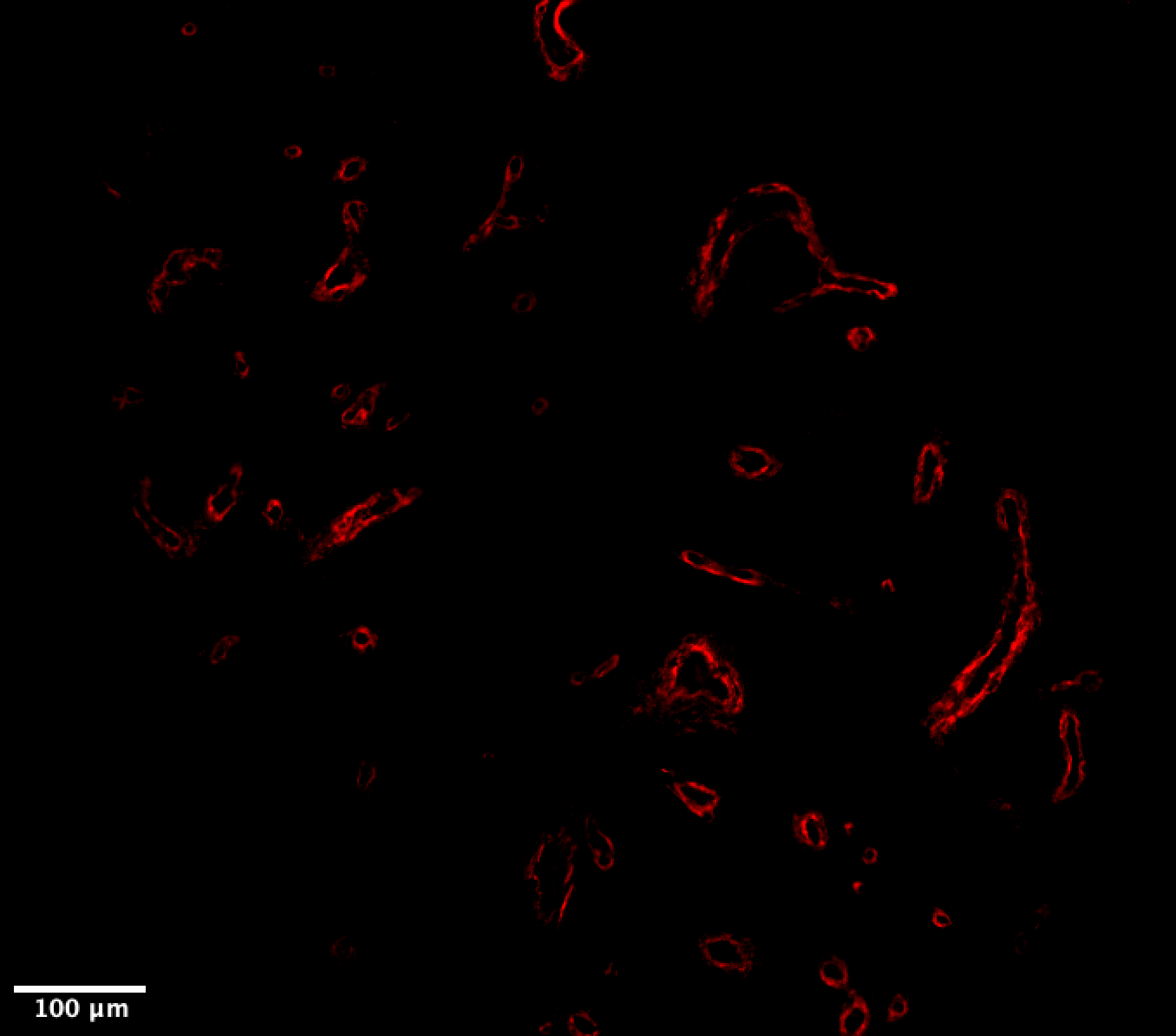

Supplement: Supplementary file 14 — Source data Fig. 7 [file 44320_2025_149_MOESM14_ESM.zip › Figure 7/7A/JRP122_COL4_Fig7 (1, x=1755, y=4032, w=2737, h=2410).png]

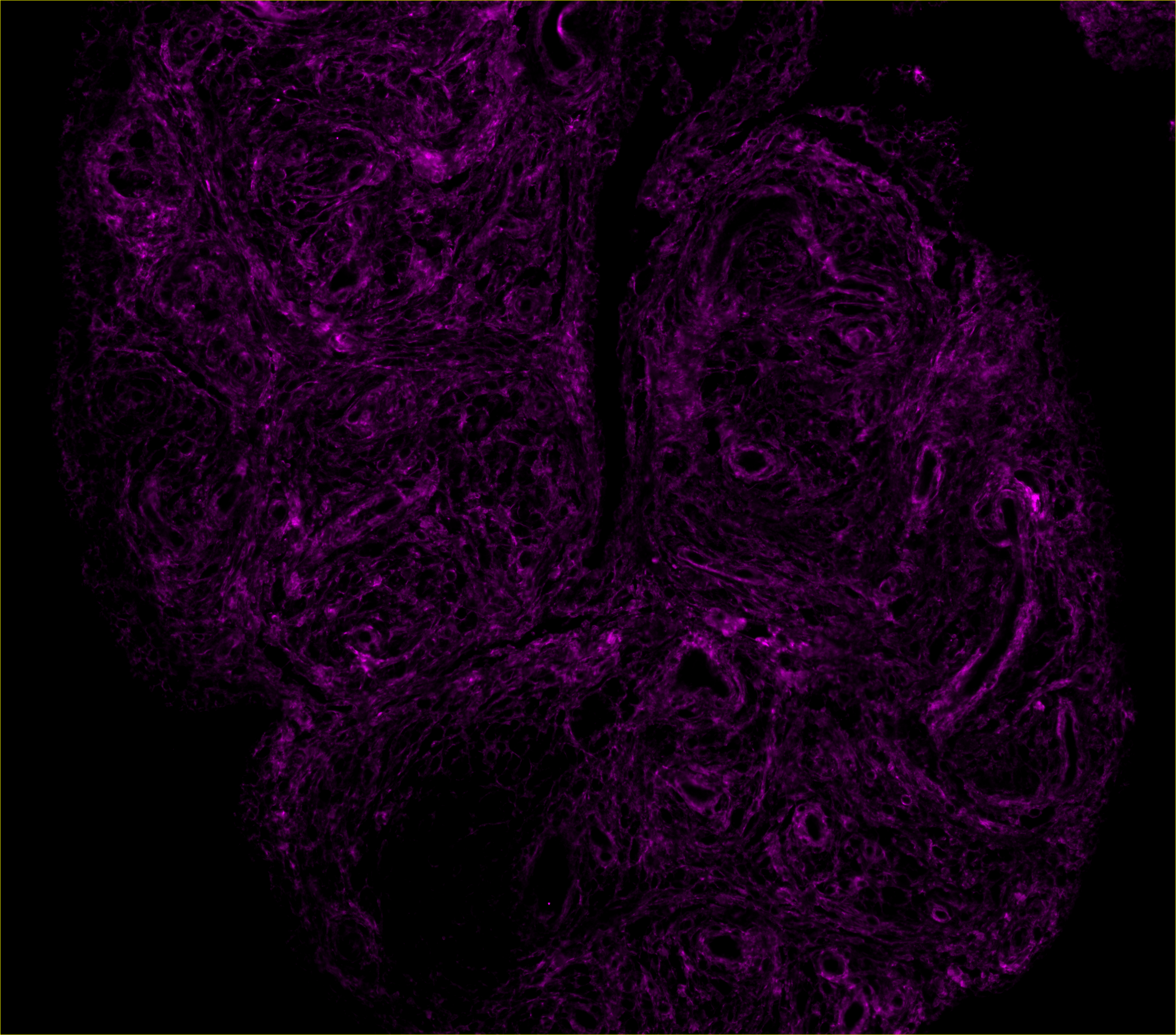

Supplement: Supplementary file 14 — Source data Fig. 7 [file 44320_2025_149_MOESM14_ESM.zip › Figure 7/7A/JRP122_COL6_Fig7 (1, x=1755, y=4032, w=2737, h=2410).tif]

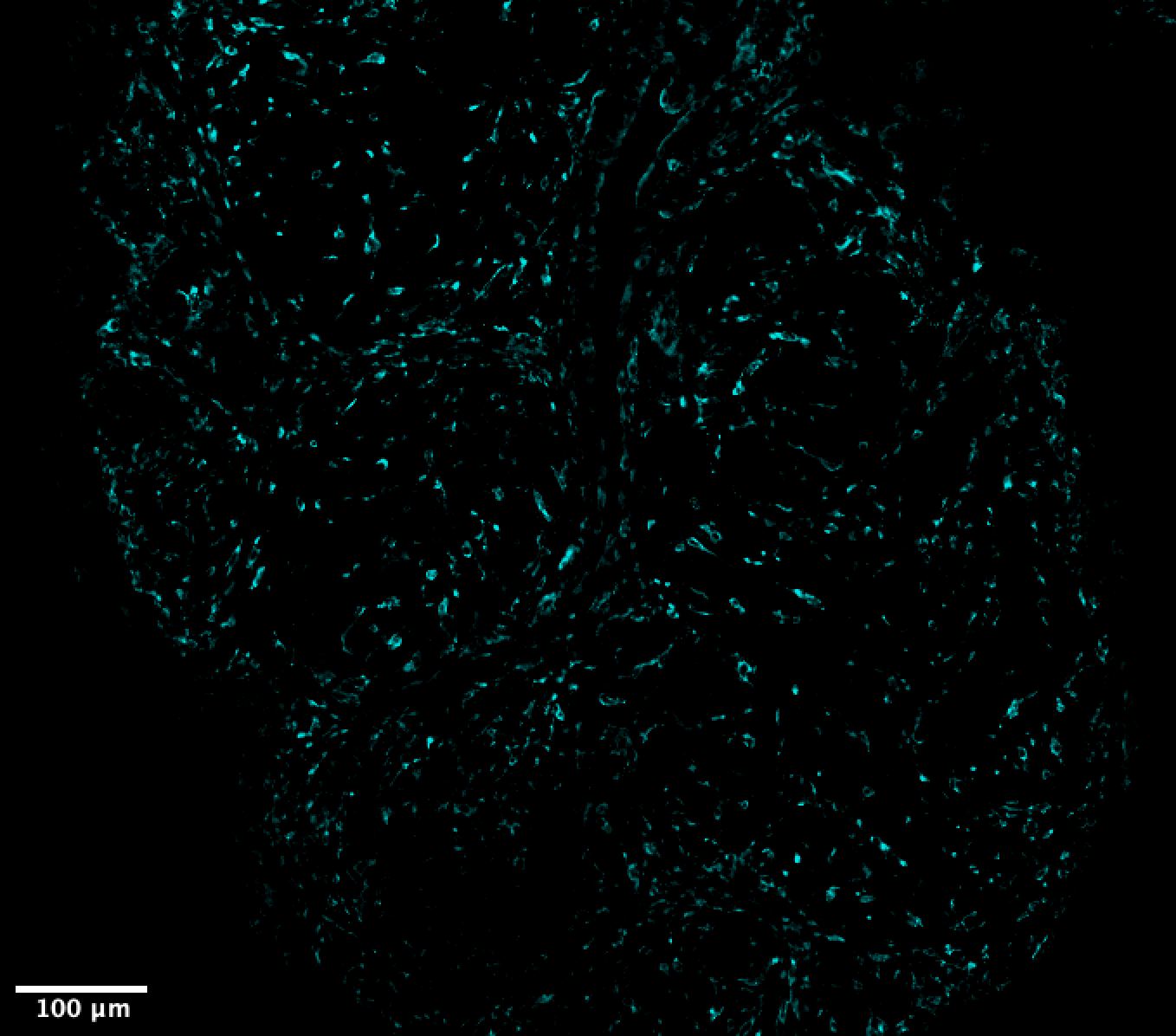

Supplement: Supplementary file 14 — Source data Fig. 7 [file 44320_2025_149_MOESM14_ESM.zip › Figure 7/7A/JRP122_CD14_Fig7 (1, x=1755, y=4032, w=2737, h=2410).png]

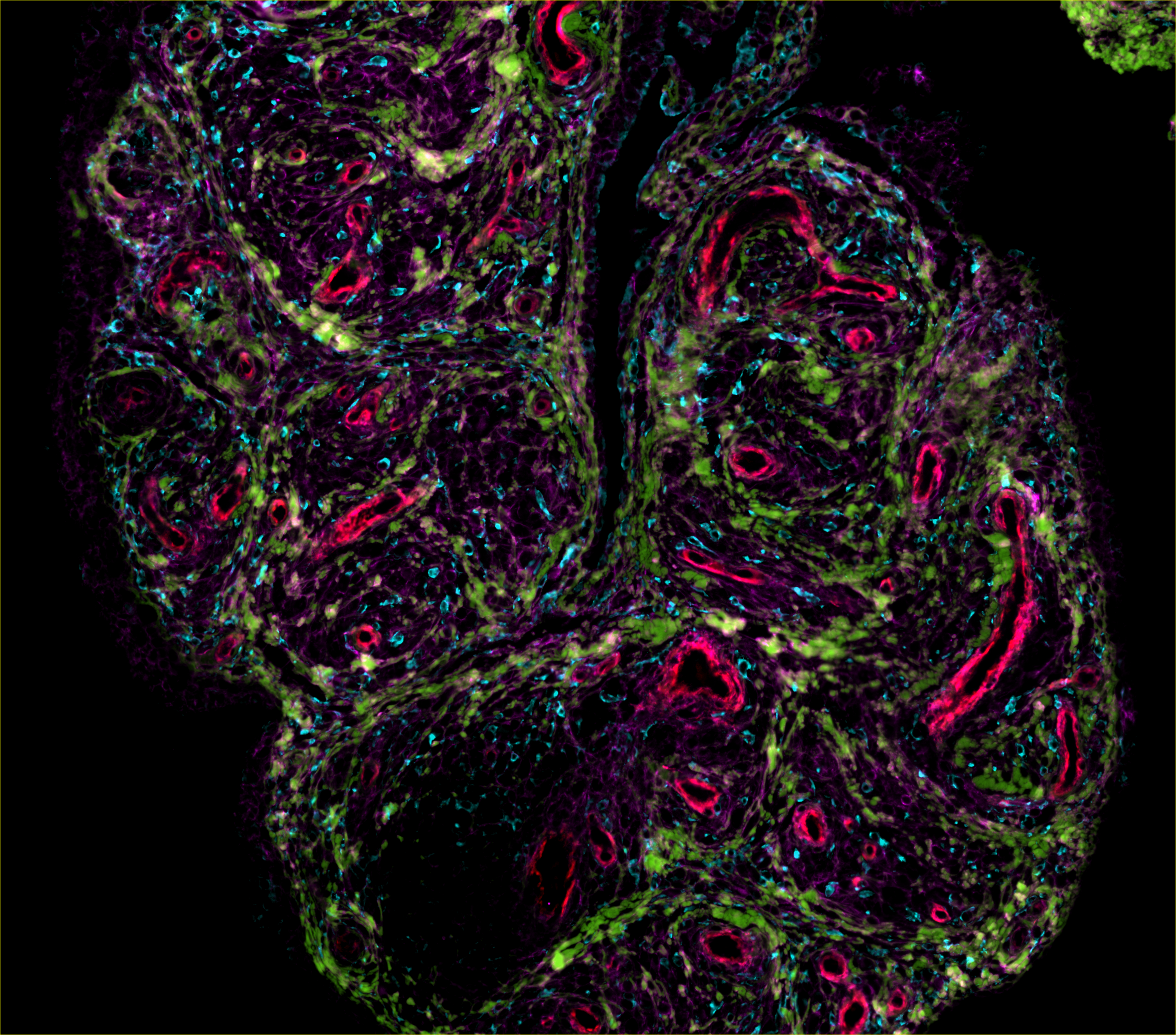

Supplement: Supplementary file 14 — Source data Fig. 7 [file 44320_2025_149_MOESM14_ESM.zip › Figure 7/7A/JRP122_Full_Fig7 (1, x=1755, y=4032, w=2737, h=2410).tif]
